# Genetic variation of the ABC transporter gene *ABCC1* (Multidrug resistance protein 1 – MRP1) in the Polish population

**DOI:** 10.1186/s12863-015-0271-3

**Published:** 2015-09-23

**Authors:** Marcin Słomka, Marta Sobalska-Kwapis, Małgorzata Korycka-Machała, Grzegorz Bartosz, Jarosław Dziadek, Dominik Strapagiel

**Affiliations:** Biobank Lab, Department of Molecular Biophysics, Faculty of Biology and Environmental Protection, University of Łódź, Pilarskiego 14/16, 90-231 Łódź, Poland; Institute of Medical Biology, Polish Academy of Sciences, Lodowa 106, 93-232 Łódź, Poland; Department of Molecular Biophysics, Faculty of Biology and Environmental Protection, University of Łódź, Pomorska 141/143, 90-236 Łódź, Poland

**Keywords:** High resolution melting, ABCC1, MRP, Gene scanning, Genotyping

## Abstract

**Background:**

Multidrug resistance-associated protein 1 (MRP1), encoded by the ABCC1 gene, is an ATP-binding cassette transporter mediating efflux of organic anions and xenobiotics; its overexpression leads to multidrug resistance. In this study, 30 exons (from 31 in total) of the ABCC1 gene as well as and their flanking intron sequences were screened for genetic variation, using the High Resolution Melting (HRM) method, for 190 healthy volunteers representing the Polish population. Polymorphism screening is an indispensable step in personalized patient therapy. An additional targeted SNP verification study for ten variants was performed to verify sensitivity of the scanning method.

**Results:**

During scanning, 46 polymorphisms, including seven novel ones, were found: one in 3’ UTR, 21 in exons (11 of them non-synonymous) and 24 in introns, including one deletion variant. These results revealed some ethnic differences in frequency of several polymorphisms when compared to literature data for other populations. Based on linkage disequilibrium analysis, 4 haplotype blocks were determined for 9 detected polymorphisms and 12 haplotypes were defined. To capture the common haplotypes, haplotype-tagging single nucleotide polymorphisms were identified.

**Conclusions:**

Targeted genotyping results correlated well with scanning results; thus, HRM is a suitable method to study genetic variation in this model. HRM is an efficient and sensitive method for scanning and genotyping polymorphic variants. Ethnic differences were found for frequency of some variants in the Polish population compared to others.

Thus, this study may be useful for pharmacogenetics of drugs affected by MRP1-mediated efflux.

**Electronic supplementary material:**

The online version of this article (doi:10.1186/s12863-015-0271-3) contains supplementary material, which is available to authorized users.

## Background

The human multidrug resistance-associated protein 1 (MRP1) is a member of the ATP-binding cassette (ABC) transporter superfamily and is encoded by the *ABCC1* gene [1]. MRP1 was first described by Cole et al. [2] who cloned the overexpressed transporter from lung cancer cell line H69AR. The gene is mapped to chromosome 16p13.1. Its length is around 200.000 base pairs; the gene contains 31 exons encoding a protein of 1,531 amino acids, with molecular weight of 190 kDa [3, 4]. The protein has 3 hydrophobic transmembrane domains (TMDs), also called membrane-spanning domains (MSDs), named TMD0 (N-terminal), TMD1 (middle) and TMD2 (C-terminal). They contain 17 transmembrane helices (TM), 5 in TMD0 and 6 in both TMD1 and TMD2. The intracellular region of the protein contains 2 hydrophilic nucleotide binding domains (NBDs), NBD1 associated with TMD1 and NBD2 associated with TMD2 [5, 6]. They are both involved in binding and hydrolyzing ATP, which is indispensable for substrate transport. Each of NBDs contains highly conserved motifs: Walker A motif which binds the β-phosphate of ATP and Walker B motif that interacts with Mg^2+^ ions. There is also a third, 13 amino acid sequence motif, between Walker A and Walker B called the ABC signature [1, 7].

MRP1 is ubiquitously expressed in many human tissues and physiological barriers like blood–brain or blood-testis barriers. High levels of MRP1 expression were detected in lung, kidney, heart, testis, placenta, adrenal glands and skeletal muscles, while lower levels of expression were found in intestine, colon, brain, peripheral blood mononuclear cells and liver [1, 3, 8]. With the exception of brain cells, MRP1, in contrast to other ABC transporters, is expressed at the basolateral membrane of polarized epithelial cells where it plays a protective role against xenobiotics and their metabolites [3, 9].

Many drugs are good substrates for MRP1, so its overexpression leads to multidrug resistance (MDR), especially during cancer chemotherapy. This effect can be observed in many types of cancer cells including solid tumors (lung cancer, breast cancer, gastric and colon carcinomas, melanoma, prostate cancer, neuroblastoma) as well as in various types of leukemias [3, 9]. MRP1-overexpressing cells are capable of transporting a large group of substrates, including anticancer drugs like folate-based antimetabolites, anthracyclines, *Vinca* alkaloids, antiandrogens and even some inorganic anions such as arsenite and arsenate. MRP1 is also an active transporter of a broad range of endogenous compounds including glutathione (GSH), glucuronate and sulfate-conjugates of organic anions, such as inflammatory mediator leukotriene C_4_ or estradiol-17-β-D-glucuronide (E_2_17βG). Therefore, MRP1 plays a crucial role in efflux of organic anions, anionic drugs, xenobiotic conjugates and additionally in the maintenance of redox balance by participation in cellular response to oxidative stress [3, 7, 9–11].

A large number of single nucleotide polymorphisms (SNPs) in *ABCC1* gene were detected so far in studies of various populations, especially Asian and Caucasian. Saito et al. [12] reported 779 genetic variations in eight ABC genes in Japanese population, 95 among them concerning *ABCC1*. Subsequently, Fukushima-Uesaka et al. [13], screening *ABCC1* in the same population, identified 86 genetic variations including 31 novel ones. Similar screening of Chinese population revealed 32 SNPs [14]. Leschziner et al. [15] screened five ABC transporter genes in Caucasian individuals and detected 221 variants, including 61 for *ABCC1*, among them 22 novel for this gene. They identified also large blocks of high linkage disequilibrium and low haplotype diversity across all the genes in the examined population. Comparative study including polymorphisms from four different populations: Chinese, Malay, Indian and Caucasian showed that apart from population-specific SNPs, only 6 of 71 detected SNPs altered amino acid sequence, so *ABCC1* is considered to be a highly conserved gene [16]. Majority of previously reported genetic variations were found in intron sequences. However, there is evidence in literature that some SNPs change protein functioning and effectiveness of cancer chemotherapy. Such cases were reported for neuroblastoma [17], breast cancer [18], ovarian cancer [19] and other serious diseases like: chronic obstructive pulmonary disease (COPD) [20, 21] cystic fibrosis [22] and major depression [23].

Previously mentioned studies have shown importance of *ABCC1* polymorphisms and its genetic variation. These data can have great relevance for pharmacogenetics in personalized patient therapy. Based on the data described above for several populations, there exist ethnic differences in the frequency of *ABCC1* polymorphic variants. Some of them are so strongly linked to each other that they are always inherited together, forming haplotypes. Haplotype is a combination of polymorphic variants inherited on the same chromosome and sometimes has stronger clinical relevance for drug response or adverse events than a single polymorphism [24]. Moreover, there is a large haplotype diversity across different populations [25]. Therefore, prior comprehensive analysis of the whole gene is required in the specific population before application of genetic variation data in pharmacotherapy.

Our previous studies performed during the project TESTOPLEK in which we have examined genetic variation of multidrug transporter genes in the Polish population indicated that *ABCC1* polymorphisms were present at significant level in a sample of Polish individuals. However, various SNPs in the *ABCC1* gene differed significantly in their frequency in tested DNA samples [26]. This prompted us to verify the whole coding sequence of *ABCC1* gene. We conducted high throughput analysis and screened 30 exons (from 31 in total) of *ABCC1* gene for polymorphism variants in the Polish population (which is exclusively Caucasians). This is the first study of genetic variation of *ABCC1* gene in the Polish population.

## Methods

### Materials and genomic DNA samples

Human genomic DNA samples were derived from anonymous Polish unrelated volunteers who declared to be healthy. Samples were randomly selected from the “normal Polish population” genetic collection at the Biobank Lab, Department of Molecular Biophysics, University of Lodz. Genetic material for this collection was sampled in 2011 – 2012 within the EU-funded TESTOPLEK project. All subjects gave their written informed consent to participate in the study. This study was approved by the relevant regional ethical committee (Research Bioethics Commission, University of Lodz - Decision no. 8/KBBN-UŁ/II/2014 and Statement of the Research Bioethics Commission, University of Lodz from 17th of June, 2010) and all procedures were performed in accordance with the Declaration of Helsinki.

Saliva was collected into Oragene OG-500 DNA collection/storage receptacles (DNA Genotek, Kanata, Canada) and genomic DNA was subsequently isolated by the MagNA Pure LC DNA Isolation Kit - Large Volume (Roche, Basel, Switzerland) with final concentration normalized to 200 pg/μl [27]. A total of 190 samples were enrolled in the scanning study and other 380 in genotyping.

### Screening of *ABCC1* by High Resolution Melting (HRM) Method

**Fig. 1:**
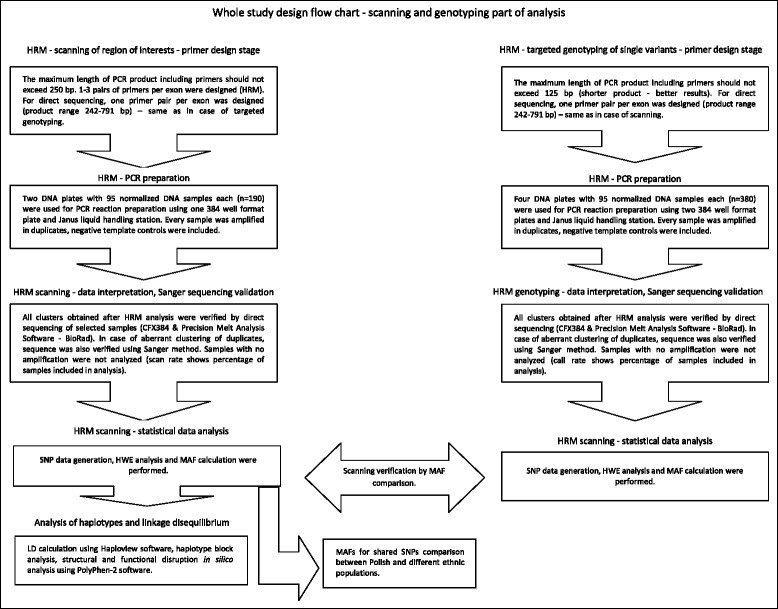
Study flow chart

Investigation of *ABCC1* genetic variation was conducted using High Resolution Melting method. The method is based on precise measurements of DNA melting profile. The dsDNA melting temperature depends on length and nucleotide composition of the PCR product. Even a single base variation generates a different melting curve, and many variants can be detected in this way. The list of primers used for HRM scanning of all the areas is presented in Table 1. The single reaction mixture (10 μl) was prepared using Janus® Automated Workstation (Perkin Elmer Inc., Waltham, USA) and composed of GoTaq® Colorless Master Mix (2×) (Promega, Madison, USA), LC Green Plus® dye (10x) (BioFire Defense Inc., Salt Lake City, USA), 0.5 μl of 10 μM primers mixture, 3 μl DNA, and filled up to the final volume with water. Reaction was performed on 384-micro well plates using CFX384™ real-time PCR system (Bio-Rad Laboratories Inc., Hercules, USA) on duplicate samples. The reaction conditions were as follows: initial denaturation at 95 °C for 3 min, 50 amplification cycles of denaturation at 95 °C for 30 s and annealing at specific temperature depending on the primers used, for 30 s. The plate was read after each cycle. Directly afterwards, melting curve was determined, the plate being incubated at 90 °C for 60 s, 40 °C for 60 s and from 65 °C to 95 °C with an increment of 0.2 °C for 10 s with plate reading. The obtained data were analysed with the Bio-Rad Precision Melt Analysis Software, version 1.2 (Bio-Rad Laboratories Inc., Hercules, USA). Based on HRM melting curve analyse, confirmation of genetic variation for every melting cluster was obtained by direct sequencing method for several samples selected from each cluster (or all samples from clusters with less than 4 samples). Study workflow design for all described methods is presented in Fig. 1.

**Table 1:** Pairs of primers used for HRM screening, sequencing and genotyping ABCC1

	Exon	Number of scanned bp upstream from the exon (without primer)	Forward primer (5’ → 3’)	Reverse primer (5’ → 3’)	Number of scanned bp downstream from the exon (without primer)	Size (bp) including primers
Scanning	1	104*	CAGCGCTAGCGCCAGCAG	CCCGGTGCTTTCCCTCCC	49	234
	2	31	GTCCTCTGGGGTGTGTCCT	TCTGAATGTAGCCTCGGTCA		201
			GCTTTCAGAACACGGTCCTC	TTATCACCAACCACCACTCC	52	203
	3	20	GGGCGGTCTGTTGTAGGATA	CCACTGAGCCCAGCAGAT	31	215
	4	129	AAGCTGAGGCAGGAGAATCA	AAGGTAGCAAGCAGCTGAGG		163
			AGCCTGGGTGACAAGAGTGA	TGGATCTCAGGATGGCTAGG		189
			ATGCTCACTTTCTGGCTGGT	GGCAACGCATGACTTCTACA	72	170
	5	27	CAGCCCCAGAATGTGATCTT	CACACACACGCACACACACT	19	212
	6	20	TTTCCCTCTTCCTCCCAAAC	AGCTGAGCATGTTCATTCGTT	59	182
	7	33	CCCTCCTCCTGTCATTGACTC	GAGTGGAAAGGAGGTGACCA		240
			GGAACAAGTCGTGCCTGTTT	ATCTTGCCCAGAACCACAAA	104	184
	8	74	TTCCCTGAAGGGTGACATTC	AGGGGTTCCACTCCTTCTGT		219
			GGAGGCTTTGATCGTCAAGT	AAAGCCAAGGAGGGAAAATG	29	199
	9	14	AGTCATTCCAGGCCCTCTCT	TGACGAAGCAGATGTGGAAG		165
			TACACCGTGCTGCTGTTTGT	CAGGAGGGGATGTGGAAGT	33	175
	10	27	TCTTCAGCTGCCACACTCAC	GCCACAGGAGGTAGAGAGCA		207
			TCTGTGGACGCTCAGAGGTT	CTGCCCACACGTAGAAGTCC	38	157
	11	21	TCTTCTGTCTGGTGAGTGATGAA	TGGACTAAAATCCTCATGGAGAG	49	209
	12	41	GTTGAGTGATGGGCTGATCC	GGCAGACTTCTTCAGCACCT		217
			GCATTCAAGGACAAGGTGCT	CCTGGGCAACATAGTGACCT	75	206
	13	62	GTCTCCAGGGCCTGTCACT	ACCGGAGGATGTTGAACAAG		184
			CCAGACAGCCTTCGTGTCTT	GCTTTCGTGGCCTAGAACC	42	149
	14	49	TTAACCTTGGTTGGTTTTGC	CAAGTAGGGAGGACCCAGTG	51	228
	15	41	GTTCTGTGCATGTGGAGTCG	GGCTCTCTCCACACCATCAC	22	179
	16	73	TTCTCTACTTGGGGTAAATTGAGG	CTGAGAGCAGGGACGACTTT		177
			ATCCCCGAAGGTGCTTTG	GCTTTTCCTCAGACCACCAG	37	171
	17	12	CTGTCTCACCTCGTTCTCCA	CTGACCTTCTCGCCAATCTC		214
			CTCCCAGACCTGGAAATCCT	CAGCCCACTGAGATTGTGAG	60	134
	18	20	ATTTCCCAGGAAACCCACTC	GGCCAATCACATTTTCAAAGA		185
			CTTCGATGATCCCCTCTCAG	CCTAAAGGGGACACGTTCTG	55	166
	19	26	CTCACACATGTGCACTCACG	TCTGTGCTGGCATAGGTACG		204
			GCCAAGCTAGGCAGTCTCAC	GGCAAGTAGCTCATGCTGTG		99
			TCTGAGATGGGCTCCTACCA	CAACCTCAAAGAGGCCAATG	56	182
	20	48	TTGTTGCCCTTGGTTTTAGC	CTCCTCTTGTCCTCCCACTG	44	223
	21	20	GCATCTGTACGGTTGACACC	TGGGCGCTCTATAAACTCCA	32	228
	22	25	GGTGCGTGCATGTGCTAAG	ATGGGGTCATCAGTCCAGAG		184
			GGCTTCCAACTATTGGCTCA	CAAAACGCTGAGGACTCTAAGG	72	204
	23	21	CCCTCTCTGCATTGTGGAGT	GTCCACTGTGTCCAGCTCCT		214
			ATGAGCTTCTTTGAGCGGAC	GGCTTGTCCCGAACACTAAG	14	244
	24	22	GAGGGAACCTTCATCAACTCC	TCTGGTTCTCGTCCACCTTC		212
			CCCATTTCAACGAGACCTTG	GGAAAAGATCGAGGCAAAGA	55	208
	25	26	AGAGCTGACTCCATGCCTGT	GGCTCCCCCATTAACAGACT	32	225
	26	24	CGCCCGCTTACTCTAGAAAT	CCAAAGACCAAGAGGTCCAG	18	184
	27	45	GGGGAGTCACAGCTTTACCA	GGGAATGGGTGAGGGAAT	18	248
	28	6	ACACCTGGGCCCTTCTGTC	TGATCTCTCCTTCGGCAGAC		113
			CTGGGCTTATTTCGGATCAA	CAGTGCAATCATAGGGCTTG	40	174
	29	55	AGTCCTTAGGTCGCCTCCAT	CCTTCTGCACATTCATGGTC		227
			CCCACCTGAAGGACTTCGT	CAAACACCCCTACCGAGATG	47	143
	30	74	GTGTCTCCTTTCGCTTCTCC	ATGGTGGACTGGATGAGGTC		217
			CCTGCTGAGGAAGACGAAGA	AGGCAAACTCCCAAAGCCTA	25	204
	31	15	CCCTTCCCCTCATGTCTGTA	ATAGGCCCTGCAGTTCTGAC	21**	185
Sequencing	1		-	-		-
	2		TCTCGAACTCCCAGCCTAAA	TAAGCGGCAGAGCAAAGATT		749
	3		GAGCATGGTGACCAGACAAA	CTCCTGACCTCAGGTGATCC		638
	4		GTGGTGAAACCCCGTCTTTA	CCTTGGAGCAACACAGACAA		604
	5		CCCGAGTAGCTGGGATTACA	ACCATGCCAAGTGAGAAACC		606
	6		GGGTTGTTGTGGGGATAGAA	AAGCAAAAGCAGTCCAGCAT		622
	7		CTGCATGACCCAACAGAATG	CAAACTCCCGACCAAGTGAT		651
	8		CTTTGACTCTGCCTTCCCTG	TAGGGGAGCCTAGTGGGTTT		691
	9		AGCAATTAGTGCACAACCCC	AAGACATCTACACGCCCACC		613
	10		ACAGCCTGGAAGCGTAGAAA	AACTGCCAGGGAGCTCTACA		664
	11		GAGCGTGGACCTGCTTATTC	CACCGGCCTACAAGGTCTAA		608
	12		GATGTTGAGTGATGGGCTGA	CCTGGGCAACATAGTGACCT		363
	13		ATACTGCCCCAGGTTTTTCC	AAAAAGGATAGGAATGGCGG		611
	14		CTGGGGAAACCCTTGAAAGT	CCAAGGGAAAGAAATGCAAG		263
	15		CCCAAGATGATCGTCCAGAT	TACGCAAGCCAAGAATCTCC		632
	16		TCTCCTTTCAGACCTTGCGT	CCTGCCTTCTAGGACAGCAC		676
	17		ACTCTTTGGAAGCAGGGGTT	AGTGAGACCTGAGCCACACC		693
	18		CTCAAGCCATCTTCCCACTC	GAAGCCAGCCCTGTGACTTA		717
	19		CATGTCCCACCTTCAGACCT	CCAGCTTAACTCCGTGCTTC		748
	20		CTGCACAGTTGCAAAGCACT	GCCAAGAGACCTGAGCAAAG		620
	21		CCGTCTCTTATGCCATGGTT	GGTTCAAATCCCAGTTCTGC		461
	22		GCATGTGCTAAGCTGCCTTAT	TGTAAAATGGGCACACTGGA		485
	23		CTCAAGTGATCCACCCACCT	TGCTTCAAAAGCACCACACT		617
	24		GAGGTTAGCACTTTGCAGCC	ATGATTCATCTGTCCTCCGC		791
	25		AAGGCAAGCTTCAGATTCCA	AAAGCATTCCCCTCGTACCT		674
	26		AAATGCCACGTGACTCTTCC	CCTGGTGAGGTATCCAGCTC		242
	27		AGGGGACAGAGGGACACAG	AAATCTGTGGGGCTCATTTG		618
	28		TCATGATGGGAAACTCACCA	GAACGATGAAGTAGGGCCAA		669
	29		AGACAGGGTGTTGCCATGTT	TCAACTGAATGGAGCTGGTG		689
	30		GGTGTGAGCTGCTGTTTTGA	AAGAGAGGATCCACCCACTG		556
	31		CCATGATTGATGTGGGGTAG	AAGCACCAGGAAACCACTTG		640
Genotyping	SNP		Forward primer (5’ → 3’)	Reverse primer (5’ → 3’)		Size (bp) including primers
	c.128G > C		TCAGAACACGGTCCTCG	ACAGGCCCAGAGGTAAAA		49
	c.616-7942A > G		AGCAACAGGGCAAACAAATC	GACGGAGCCTAAATGTCCAG		76
	**c.816G > A**		**CTTGTGTTCCAGGCAGC**	**GCAGGATCCTTGGAGGAGTA**		**51**
	**c.825 T > C**					
	c.1057G > A		GCTCCTTTGCAGGTTGCTCATC	GGGGCCTTCGTGTCATTCA		48
	**c.1062 T > C**		**GGTTGCTCATCAAGTTCGTGA**	**GTCTGGGGCCTTCGTGTC**		**41**
	**c.1218 + 8A > G**		**CATGAGGATCAAGACCGCTG**	**GTGGAAGTCGGGCCACAT**		**84**
	**c.1299G > T**		**CCAGAAAATCCTCCACGGT**	**GTACGTGGCCAAGTCCATGA**		**80**
	**c.1684 T > C**		**GTTCTGCTTGCAGGAGGCC**	**TTCAGGGGAAACCGGAGGAT**		**125**
	**c.1704C > T**					
	**c.2012G > T**		**TCACCTTCTCCATCCCCG**	**CACTTTGTCCATCTCAGCCA**		**104**
	**c.2168G > A**		**CAGGCCTGGATTCAGAATGA**	**ATGGTTCCTCCAGCTGACAT**		**67**
	c.2581G > A		CAGGAGCTGCTGGCTCG	TCTGTGCTGGCATAGGTACG		62
	c.2965G > A		TCCTTTTCATGTGTAACCATGTG	CAATAGTTGGAAGCCAGCG		46
	c.3140G > C		CTACTCCATGGCCGTGTCC	AGGATGCTGTGCAGCAGGT		75
	c.3436G > A		GAGGTTCTACGTGGCTTCC	GAATAGACCGGGGAGCG		69
	**c.4002G > A**		**GACAAGTCCGGATGCCAG**	**CGAAATAAGCCCAGGGTC**		**112**
	c.4009A > G		AGCTGGGAAGTCGTCCCT	TGATCTCTCCTTCGGCAG		65

104* - upstream from the START codon. 21** - downstream from the STOP codon. Bold variants means these ones which genetic variation was detected during genotyping method

### HRM genotyping

Independently of the scanning study, we performed an additional genotyping study. We focused on highly polymorphic regions of the *ABCC1* gene existing in databases, often examined in recent publications. We also tested some interesting variants observed in literature that may have clinical significance. The selection provided us with 17 SNPs to check. To identify common genetic variation of selected SNPs in the *ABCC1* gene, we used the HRM analysis method according to conditions described above. Sequences of primers used for genotyping are described in Table 1. Based on HRM melting curve analysis, confirmation of genetic variation for every melting cluster was obtained by direct sequencing method for several samples selected from each cluster (or all samples from clusters less than 3 samples). We used the data obtained from genotyping to compare them with HRM scanning results (comparison of minor allele frequencies - MAFs) and thereby to validate the accuracy of the screening method.

### DNA sequencing

For sequencing, selected samples were prepared by PCR. Reaction mixture filled with water to a final volume of 50 μl per sample was composed using GoTaq® Green Master Mix (2×) (Promega, Madison, USA), 5 μl of 10 μM primers mix and 5 μl DNA. Reaction conditions were as follows: an initial denaturation at 95 °C for 3 min, 45 amplification cycles of denaturation at 95 °C for 30 s, annealing at specific temperature depending on the primers used for 30 s and extension at 72 °C for 45 s, followed by final extension at 72 °C for 5 min (list of primers for exon sequencing is shown in Table 1). Afterwards, PCR products were purified using NucleoSpin® Gel and PCR Clean-up kit (Macherey-Nagel GmbH & Co. KG, Düren, Germany). DNA concentration and size of PCR product was determined by agarose electrophoresis of 2 μl of each sample. Based on the intensity of bands the samples were diluted from 5 to 50 times and applied for sequencing reaction using BigDye Terminator V3.1 (Applied Biosystems, USA) according to the manufacturer's protocol. The PCR-sequencing product was purified using BigDye X-Terminator kit following the manufacturer's protocol (Applied Biosystems, USA). Further, 30 μl of each purified sample was applied to 96 wells of titration plates and analyzed in 3500 Genetic Analyzer (Applied Biosystems, USA).

### Polymorphism detection

The *ABCC1* genomic DNA sequence (NG_028268.1) was obtained from GenBank (http://www.ncbi.nlm.nih.gov) and used as a reference sequence during analysis of sequencing results by CodonCode Aligner software (CodonCode Corporation, Centerville, USA). Sequencing results of selected samples were compared with respective HRM clusters. Based on this, genotype for each melting cluster was established and genetic variation for each sample was verified.

### Availability of supporting data

For each of the polymorphisms detected in this study in *ABCC1* gene, the following parameters were assigned: dbSNP IDs (rs numbers) (http://www.ncbi.nlm.nih.gov/SNP/), nucleotide position within or relative to the coding sequence based on the NM_004996.3, and amino acid position in the protein for SNPs in the coding sequence based on the reference sequence NP_004987.2. These data were obtained from GenBank (http://www.ncbi.nlm.nih.gov) and were used in this paper as the nomenclature of variants.

The obtained data as supporting materials are presented in details in Additional files 1, 2, 3 and 4.

### Prediction of impact of SNPs upon protein functionality

Using PolyPhen-2 v2.2.2r398 tool (http://genetics.bwh.harvard.edu/pph2/), we performed automatic prediction of effects of missense SNPs and amino acid substitutions for protein functionality and structure. PolyPhen-2 uses damaging alleles datasets, like HumDiv or HumVar, to calculate naïve Bayes posterior probability and to classify a mutation deleterious effect as benign, possibly damaging or probably damaging. This ternary classification depends on thresholds of false positive rate (FPR) denoting the chance that mutation would be falsely qualified as deleterious. For HumDiv-trained model, the thresholds are 5 % and 10 % FPR while for HumVar-trained model they are 10 % and 20 % FPR, respectively [28].

### Linkage disequilibrium and haplotype blocks analysis

The observed genotype distribution was determined for all the detected polymorphisms by performing the Hardy-Weinberg equilibrium (HWE) exact test assuming consistency for P-value higher than 0.001. Linkage disequilibrium (LD) and haplotype block analysis was performed by Haploview 4.2 software (http://www.broad.mit.edu/mpg/haploview/). The LD analysis was made using |D’| and r^2^ parameters for each variation pair. |D’| coefficient was preferentially used to model recombination rates in examined population, and r^2^ parameter to model association power [29, 30].

Studying haplotypes in the human genome requires definition of the location of haplotype blocks, which are regions with little evidence for historical recombination and within which only a few common haplotypes are observed [31]. To determine haplotype blocks in *ABCC1* for the Polish population, we used the method described previously by Gabriel et al. [31] based on confidence bounds on D’. For pairs of SNPs being in strong linkage, 95 % upper confidence bound D’ was higher than 0.98 and the lower bound was above 0.7 while for pairs of SNPs with strong evidences of historical recombination upper bound on D’ is lower than 0.9. According to this model, polymorphisms with MAF [22] lower than 0.05 were excluded during the creation of blocks and 16 polymorphic variants with higher MAF were included. Haplotypes with frequency lower than 1 % were grouped as “others”.

## Results

### ABCC1 genetic variation detected in this study

We screened coding regions and exon/intron boundaries as the most important gene areas (Additional file 1). To assure optimal detection level, the maximal length of tested PCR product was never longer than 250 and 125 base pairs for scanning and genotyping, respectively. For scanning, most of exons had to be divided into separate scanned areas, which provided 51 fragments for analysis. We performed complete polymorphism screening in 30 exons in total. We were unable to scan exon 1 because of problems with amplification during PCR, caused by extremely GC-rich sequence region.

In the scanning study on 190 Polish volunteers, we found 46 different SNPs in the scanned areas, and seven of them being novel and not reported yet (Table 2). 21 SNPs were located in exons and 11 of them change amino acid sequence as non-synonymous variants, including one novel polymorphic variant detected in this study: c.596C > T (p.Ser199Leu) with MAF = 0.003. Among all detected non-synonymous variants, only one, c.2012G > T (p.Gly671Val), occurred as a homozygote with estimated MAF = 0.077. The rest of SNPs found homozygously in exons were synonymous variants which resided at: c.825 T > C (p.Val275=), c.1062 T > C (p.Asn354=), c.1684 T > C (p.Leu562=), c.1704C > T (p.Tyr568=) and c.4002G > A (p.Ser1334=) with MAF values equal to 0.309, 0.332, 0.130. 0.088 and 0.277, respectively. Apart from polymorphisms in coding regions, 23 SNPs were located in introns and a last one (c.*3C > G) in the 3’ UTR. Furthermore, one intron deletion variant c.2461-39_2461-38delAT was found during the scanning. Two polymorphic variants located in introns, c.2871 + 26C > T and c.3079 + 10G > A, occurred only as non-reference homozygotes in all subjects.

**Table 2:** Summary of ABCC1 variants detected during scanning by HRM

Exon scanned by HRM	dbSNP ID	Variant position NM_004996.3:	Intron/amino acid residue NP_004987.2:	Observed genotypes^a, b^ (n)			HWE exact test P-value^c^	MAF^d^
				R/R	R/V	V/V		
2	rs8187843	c.225 + 26G > A	Intron	164	25	0	1	(A) 0.066
4	rs587783373*	c.352-79G > A	Intron	185	1	0	1	(A) 0.003
4	rs4148337	c.352-66 T > C	Intron	15	80	91	0.727	(T) 0.296
5	rs483352860*	c.596C > T	p.Ser199Leu	186	1	0	1	(T) 0.003
6	rs8187846	c.677 + 17C > T	Intron	188	1	0	1	(T) 0.003
7	rs483352864*	c.809 + 16C > T	Intron	188	1	0	1	(T) 0.003
7	rs45609533	c.809 + 31G > T	Intron	183	5	0	1	(T) 0.013
7	rs903880	c.809 + 54C > A	Intron	112	65	11	0.684	(A) 0.231
7	rs246232	c.809 + 64C > G	Intron	84	90	14	0.174	(G) 0.314
8	rs546943313	c.810-73C > T	Intron	187	1	0	1	(T) 0.003
8	rs200194736	c.814C > T	p.Pro272Ser	187	1	0	1	(T) 0.003
**8**	**rs2230669**	**c.816G > A**	**p.Pro272=**	**172**	**16**	**0**	**1**	**(A) 0.043**
**8**	**rs246221**	**c.825 T > C**	**p.Val275=**	**84**	**92**	**12**	**0.059**	**(C) 0.309**
8	rs587783372*	c.855G > A	p.Pro285=	187	1	0	1	(A) 0.003
**9**	**rs35587**	**c.1062 T > C**	**p.Asn354=**	**78**	**91**	**16**	**0.185**	**(C) 0.332**
**9**	**rs35588**	**c.1218 + 8A > G**	**Intron**	**82**	**91**	**16**	**0.245**	**(G) 0.327**
9	rs483352877*	c.1218 + 9C > T	Intron	188	1	0	1	(T) 0.003
**10**	**rs60782127**	**c.1299G > T**	**p.Arg433Ser**	**186**	**2**	**0**	**1**	**(T) 0.005**
12	rs17265551	c.1677 + 56C > T	Intron	162	27	0	0.604	(T) 0.072
13	rs35604	c.1678-37G > A	Intron	2	45	142	0.745	(G) 0.130
13	rs483352863*	c.1678-34G > A	Intron	188	1	0	1	(A) 0.003
**13**	**rs35605**	**c.1684 T > C**	**p.Leu562=**	**2**	**45**	**142**	**0.745**	**(T) 0.130**
**13**	**rs8187858**	**c.1704C > T**	**p.Tyr568=**	**157**	**31**	**1**	**1**	**(T) 0.088**
14	rs112282109	c.1898G > A	p.Arg633Gln	187	1	0	1	(A) 0.003
16	rs8187863	c.2001C > T	p.Ser667=	187	1	0	1	(T) 0.003
**16**	**rs45511401**	**c.2012G > T**	**p.Gly671Val**	**161**	**25**	**2**	**0.296**	**(T) 0.077**
**17**	**rs4148356**	**c.2168G > A**	**p.Arg723Gln**	**181**	**9**	**0**	**1**	**(A) 0.024**
19	rs45607032	c.2461-39_2461-38delAT	Intron	179	9	0	1	(delAT) 0.024
19	rs2074087	c.2461-30C > G	Intron	0	44	144	0.083	(C) 0.117
19	rs45492500	c.2461-27G > A	Intron	172	14	2	0.056	(A) 0.048
21	rs11075296	c.2871 + 26C > T	Intron	0	0	189	1	-
22	rs768191257	c.2876A > G	p.Lys959Arg	187	1	0	1	(G) 0.003
22	rs3851716	c.3079 + 10G > A	Intron	0	0	188	1	-
22	rs34794353	c.3079 + 24C > T	Intron	187	1	0	1	(T) 0.003
22	rs3887893	c.3079 + 62 T > C	Intron	67	96	25	0.358	(C) 0.388
23	rs191017838	c.3171G > A	p.Leu1057=	187	2	0	1	(A) 0.005
23	rs199773531	c.3196C > T	p.Arg1066Trp	188	1	0	1	(T) 0.003
25	rs41278168	c.3591-5C > T	Intron	187	1	0	1	(T) 0.003
27	rs200922662	c.3886C > T	p.Arg1296Trp	187	1	0	1	(T) 0.003
27	rs201533167	c.3901C > T	p.Arg1301Cys	187	1	0	1	(T) 0.003
**28**	**rs2230671**	**c.4002G > A**	**p.Ser1334=**	**102**	**68**	**18**	**0.202**	**(A) 0.277**
28	rs188980645	c.4093G > A	p.Asp1365Asn	187	1	0	1	(A) 0.003
29	rs212087	c.4126-45G > A	Intron	62	85	42	0.239	(A) 0.447
30	rs212088	c.4487 + 18G > A	Intron	136	47	5	0.775	(A) 0.148
31	rs587783374*	c.4551G > A	p.Gln1517=	186	1	0	1	(A) 0.003
31	rs373453875	c.*3C > G	3’ UTR	186	1	0	1	(G) 0.003

*Novel polymorphic variants detected in this study. ^a^Number of genotypes detected during this study, R – reference allele, V – variant allele. ^b^Total number of examined samples was 190, however during scanning single samples were excluded because of accidental problems with amplification during PCR and hence, total number of genotypes is not equal precisely 190. ^c^P-value is consistent with Hardy-Weinberg equilibrium if P > 0.001. ^d^Minor allele shown in brackets with its frequency. Bold variants signifies the ones which were validated by genotyping results

We used the data from genotyping study to validate our scanning results and compare accuracy of HRM methods (Additional file 2). For genotyping, we tested a subset of 17 SNPs, selected according to the literature or detected during scanning. Seven of them have not been found during scanning in examined samples and also did not show any genetic variation by genotyping method. Genotype validation by sequencing was performed for all of examined SNPs (Table 3). We calculated MAFs to enable us to assess the accuracy of scanning method compared to genotyping. Results obtained for eight of SNPs were quite similar and the differences were not statistically significant (Table 3). We found statistically significant difference in MAF values obtained in the both our studies for only two *loci* – c.1062 T > C (p.Asn354+) and c.2012G > T (p.Gly671Val).

**Table 3:** Summary of ABCC1 selected SNPs genotyping by HRM and comparing them with scanning results

dbSNP ID	Variant residue NM_004996.3:	Intron/amino acid residue NP_004987.2:	Observed genotypes^a^ (n)			HWE exact test *P*-value^b^	MAF^*c*^ (genotyping)	MAF^c^ (scanning)	Chi-square test *P*-value^d^
			R/R	R/V	V/V				
rs41395947	c.128G > C	p.Cys43Se	380	0	0	1	--	--	--
rs2230669	c.816G > A	p.Pro272=	362	18	0	1	(A) 0.024	(A) 0.043	0.079
rs246221	c.825 T > C	p.Val275	197	160	23	0.243	(C) 0.271	(C) 0.309	0.187
rs8187852	c.1057G > A	p.Val353Met	379	0	0	1	--	--	--
rs35587	c.1062 T > C	p.Asn354=	204	142	33	0.247	(C) 0.274	(C) 0.332	0.044
rs35588	c.1218 + 8A > G	Intron	190	160	30	0.709	(G) 0.289	(G) 0.325	0.214
rs60782127	c.1299G > T	p.Arg433Ser	373	6	0	1	(T) 0.008	(T) 0.005	0.623
rs35605	c.1684 T > C	p.Leu562=	13	105	262	0.588	(T) 0.172	(T) 0.130	0.063
rs8187858	c.1704C > T	p.Tyr568=	325	55	0	0.242	(T) 0.072	(T) 0.087	0.374
rs45511401	c.2012G > T	p.Gly671Val	346	28	3	0.007	(T) 0.045	(T) 0.077	0.038
rs4148356	c.2168G > A	p.Arg723Gln	360	19	0	1	(A) 0.025	(A) 0.024	0.888
rs45517537	c.2581G > A	p.Ala861Thr	380	0	0	1	--	--	--
rs35529209	c.2965G > A	p.Ala989Thr	378	0	0	1	--	--	--
rs13337489	c.3140G > C	p.Cys1047Ser	380	0	0	1	--	--	--
rs28706727	c.3436G > A	p.Val1146Ile	380	0	0	1	--	--	--
rs2230671	c.4002G > A	p.Ser1334=	204	140	32	0.296	(A) 0.271	(A) 0.277	0.850
rs28364006	c.4009A > G	p.Thr1337Ala	380	0	0	1	--	--	--

^a^Number of genotypes detected during this study, R – reference allele, V – variant allele. ^b^P-value is consistent with Hardy-Weinberg equilibrium if *P* > 0.001. ^c^Minor allele shown in brackets with its frequency. ^d^P-value of Chi-square test with Yates’ correction, no significant difference between MAFs if *P* > 0.05

All the amino acids altered by non-synonymous variants were located in cytoplasmic domains of the protein. Variants c.596C > T (p.Ser199Leu) and c.814C > T (p.Pro272Ser) were located in the third intracellular loop (between TM5 and TM6), variant c.1299G > T (p.Arg433Ser) in the fourth intracellular loop, variant c.3196C > T (p.Arg1066Trp) in the seventh intracellular loop (between TM7 and TM8). Additional four variants located in the loop containing NBD1 alter amino acids sequence: c.1898G > A (p.Arg633Gln) and c.2012G > T (p.Gly671Val) are located 44 and 6 amino acids upstream of the Walker A motif, respectively, while c.2168G > A (p.Arg723Gln) and c.2876A > G (p.Lys959Arg) are located 37 amino acids downstream of this motif, respectively. Similarly, three variants changing amino acids in the loop containing NBD2 were detected: c.3886C > T (p.Arg1296Trp) and c.3901C > T (p.Arg1301Cys) are located 30 and 25 amino acids upstream of the Walker A motif, respectively, while c.4093G > A (p.Asp1365Asn) is located 30 amino acids downstream of the motif. Detected variant c.4002G > A (p.Ser1334=) occurred in the last position of Walker A motif in NBD2, however it does not change the amino acid sequence.

Analysis of all the non-synonymous variants detected in this study by the PolyPhen-2 tool (data in Additional file 3) showed for HumDiv-trained model that five of them: c.814C > T (p.Pro272Ser), c.1898G > A (p.Arg633Gln), c.2168G > A (p.Arg723Gln), c.2876A > G (p.Lys959Arg), the novel one c.4093G > A (p.Asp1365Asn), probably have benign influence on the functioning of the protein. For one polymorphism, c.3196C > T (p.Arg1066Trp) a possibly damaging effect on protein activity was expected. The novel variant c.596C > T (p.Ser199Leu) was estimated as a probably damaging substitution, likewise as four others: c.1299G > T (Arg433Ser), c.2012G > T (p.Gly671Val), c.3886C > T (p.Arg1296Trp) and c.3901C > T (p.Arg1301Cys). On the other hand, analysis for HumVar-trained model indicated that three polymorphisms: c.1299G > T (Arg433Ser), c.2012G > T (p.Gly671Val), c.3901C > T (p.Arg1301Cys), lead to probably damaging substitutions and two others, c.596C > T (p.Ser199Leu) and c.3886C > T (p.Arg1296Cys), are possibly damaging variants.

### Linkage disequilibrium analysis

Based on full genotype sets of 44 polymorphic variants confirmed by Hardy-Weinberg equilibrium exact test (Table 2), linkage disequilibrium analysis using r^2^ and |D’| statistics was performed (Additional file 4). Accordingly to the r^2^ parameter, perfect linkage (r^2^ = 1) was detected between the new variant c.809 + 16C > T and c.3196C > T, between c.1678-37G > A and c.1684 T > C, between c.2168G > A and c.2461-39_2461-38delAT. Almost perfect linkage was observed between c.1062 T > C and c.1218 + 8A > G (r^2^ = 0.928), between c.825 T > C (p.Val275=) and c.1062 T > C (p.Asn354=) (r^2^ = 0.822) and between c.825 T > C (p.Val275=) and c.1218 + 8A > G (r^2^ = 0.894). Apart from perfect linkage between variants c.1678-37G > A and c.1684 T > C, they were both strongly linked (r^2^ = 0.705) with c.2461-30C > G. Other linkages (r^2^ = 0.624 and r^2^ = 0.658, respectively) were found between c.225 + 26G > A and c.816G > A, and between c.809 + 54C > A and c.809 + 64C > G.

For the |D’| values, perfect linkage (|D’| = 1) was observed for the great majority of variants pairs (760 for 946). Strong linkage (1 > |D’| ≥ 0.8) was detected for other 17 pairs. Small areas of low |D’| values, and hence weak or lacking of linkage, were defined between polymorphisms from group: c.225 + 26G > A, c.352-66 T > C, c.809 + 31G > T, c.809 + 54C > A, c.809 + 64C > G, c.816G > A, c.825 T > C, c.1062 T > C, c.1218 + 8A > G, c.1677 + 56C > T, and those from group: c.1678-37G > A, c.1684 T > C, c.1704C > T, c.2012G > T, c.2168G > A, c.2461-39_2461-38delAT, c.2461-30C > G, c.2461-27G > A, c.3079 + 62 T > C, c.4002G > A, c.4126-45G > A, c.4487 + 18G > A.

### Haplotype block analysis

Based on the model of Gabriel et al. [31] a total of 12 common haplotypes were identified and their frequencies were defined in four blocks (Table 4). In block 1, variants c.809 + 54C > A and c.809 + 64C > G were included, and the most frequent haplotype in this block was that containing major alleles (frequency: 0.686), the next most frequent one was the haplotype with both minor alleles (frequency: 0.231). Block 2 spanned SNPs c.825 T > C (p.Val275=), c.1062 T > C (p.Asn354=) and c.1218 + 8A > G at a distance of 1 kb, with the most frequent haplotype containing all major alleles (frequency: 0.659), subsequently the haplotype with all minor alleles (frequency: 0.300), two other ones had low frequency (set alleles TCG: 0.021 and set alleles TCA: 0.011). Block 3 had two haplotypes composed of SNPs c.1678-37G > A and c.1684 T > C (p.Leu562=). The first haplotype was the set with major alleles (frequency: 0.870) and the second one the set with minor alleles (frequency: 0.130). Block 4 was created of SNPs c.4126-45G > A and c.4487 + 18G > A at a distance of around 2 kb. The most frequent haplotype was that of alleles combination AG (frequency: 0.447), the next ones with major alleles (frequency: 0.405) and allele combination GA (frequency: 0.148). The frequency of recombination was also calculated between blocks as a value of multiallelic D’ coefficient, and equaled to 0.65 between block 1 and 2, 0.55 between block 2 and 3 and 0.27 between block 3 and 4. HaploView predicted 51 possible connections of haplotypes for recombination between blocks higher than 1 %. However, recombination between blocks higher than 10 % occurred only between the most frequent haplotype from block 1 and the most frequent one from block 2, between three most frequent haplotypes from block 2 and the most frequent one from block 3, and between the most frequent one from block 3 and three haplotypes from block 4. Apart from c.1684 T > C (p.Leu562=), the remaining SNPs in blocks were tagged *in silico* as haplotype tag SNPs (htSNPs).

**Table 4:** Haplotype block analysis according to Gabriel et al. [31]

	Block 1			Recombination between blocks^d^	Block 2				Recombination between blocks^d^	Block 3			Recombination between blocks^d^	Block 4		
Variant position^a,b^	**c.809 + 54C >A**	**c.809 + 64C>G**	Haplotype frequency		**c.825 T>C p.Val275=**	**c.1062 T>C p.Asn354=**	**c.1218 + 8A>G**	Haplotype frequency		**c.1678-37G > A**	c.1684 T > C p.Leu562=	Haplotype frequency		**c.4126-45G > A**	**c.4487 + 18G N A**	Haplotype frequency
Haplotypes^c^	C	C	0.686	0.65	T	T	A	0.659	0.45	A	C	0.870	0.27	A	G	0.447
	A	G	0.231		C	C	G	0.300		G	T	0.130		G	G	0.405
	C	G	0.082		T	C	G	0.021						G	A	0.148
	others		0.001		T	C	A	0.011								
					others			0.009								

^a^Position according to reference sequences for coding nucleotides NM_004996.3 and amino acid residues NP_004987.2. ^b^Bold variants signify haplotype tag SNPs (htSNPs). ^c^Major alleles in white boxes, minor alleles in shaded boxes; all the haplotypes with their frequencies in population; haplotypes below 1 % grouped as “others”. ^d^Level of recombination between blocks as a value of multiallelic D’

## Discussion

In this paper we highlighted the fact that the High Resolution Melting method is a powerful technique both for targeted genotyping of selected SNPs and for scanning of long parts of gene sequences. The obtained results for targeted genotyping and scanning of *ABCC1* gene regions did not show statistically significant differences in MAFs values for most SNPs. Marginal statistical significance (0.05 > P > 0.01) of difference between MAF values for scanning and targeted genotyping methods for two SNPs c.1062 T > C (p.Asn354=) and c.2012G > T (p.Gly671Val) is probably caused by sample size effect (there was no overlap between groups of individuals selected for both stages of the study). Seven SNPs from a subset of 17 individually genotyped ones were not detected during the scanning and did not show any genetic variation during genotyping either. Although these variants were previously indicated to may have clinical significance and were therefore included in our study, they were only identified during *in vitro* studies or in non Caucasian populations (Exome Aggregation Consortium (ExAC), Cambridge, MA (http://exac.broadinstitute.org), date accessed: Aug 2015). Thus, it was expected not to find them in our study for 380 genotyped samples, where technical or methodological problems with detection should not have caused any discrepancy.

During the scanning study we identified 46 polymorphic variants by screening 30 exons of the *ABCC1* gene with adjacent intronic fragments in 190 unrelated Polish individuals. A large fraction of variants represented rare or even completely novel (not previously published) ones. We were unable to compare them with the ones from the only other publication on *ABCC1* variant distribution in Caucasians because of unavailability of the raw data from that study [15].

Comparing our genetic variation results obtained using the scanning method with data for Asian populations, 18 variants were detected in the Polish population which were present also in the Japanese one (*n* = 153) [13], while 13 variants from the Polish population were also detected in the Chinese one (*n* = 27) [14]. Some of these variants exhibited significant differences in MAF values, confirmed by chi-square test for alleles (with Yates’ correction if any allele quantity was below 5) and showed as their P-values, assumed significant if <0.05. Higher MAF for the Polish population was detected for variant c.809 + 54C > A (MAF = 0.231, compared to 0.059 for Japanese (*P* < 0.001) and 0.093 for Chinese (*P* = 0.020) populations), synonymous SNP c.4002G > A (MAF = 0.277, compared to 0.196 for Japanese (*P* = 0.014) and only 0.111 for Chinese (*P* = 0.009) populations) and for variant c.4126-45G > A between Polish (MAF = 0.447) and Japanese (MAF = 0.304) populations (P < 0.001). On the other hand, lower MAF for Polish population was noticed for variants: c.809 + 64C > A (0.313 for Polish, compared 0.418 (*P* = 0.004) for Japanese and 0.481 (*P* = 0.014) for Chinese populations), c.1678-37G > A and synonymous SNP c.1684 T > C (for both SNPs, MAF = 0.130 for Polish, 0.245 (P < 0.001) for Japanese but not significant for Chinese 0.204 (P = 0.141)), non-synonymous SNP c.2168G > A and deletion variant c.2461-39_2461-38delAT (for both variants, MAF = 0.024 for Polish, 0.065 (*P* = 0.007) for Japanese and not significant for Chinese 0.065 (*P* = 0.108)), c.2461-30C > G (for Polish MAF = 0.117, not significant for Chinese 0.167 (*P* = 0.299) but for Japanese 0.245 (*P* < 0.001)) and c.4487 + 18G > A between Polish (MAF = 0.148) and Japanese (MAF = 0.291, *P* < 0.001) populations. These results suggest that although a wide group of common variants for different populations have had similar MAF, there are also other polymorphisms which MAF values were significantly different. Since differences between Asian populations were consistently smaller than between them and the Polish population, we can conclude that ethnic ancestry is probable underlying cause of these variations. Similar conclusions were described previously during research of four polymorphisms in *ABCC1* gene in Caucasians: c.816G > A, c.825 T > C, c.1684 T > C, c.4002G > A [32]. All these results correspond to those obtained by Wang et al. [16] who studied 60 SNPs and 10 deletion variants for four populations. During our *ABCC1* scanning, we detected 25 common variants and obtained similar results for Polish population as those authors for Caucasian representatives. A slight difference in MAF was observed for few polymorphisms: the highest were seen for c.809 + 64C > G (MAF = 0.313 for Polish and 0.200 (*P* = 0.043) for Caucasians) and for c.825 T > C (p.Val275=) (MAF = 0.309 for Polish population and 0.139 (*P* = 0.003) for Caucasians), while the difference for c.4002G > A (MAF = 0.277 and 0.375 (*P* = 0.093) for Polish and Caucasians, respectively) was not significant. Furthermore, their results confirmed our previous observations concerning ethnic differences in occurrence of the same polymorphic variants between Caucasian and Asian populations. These differences relate mainly to the SNPs mentioned previously as most ethnically significant: c.809 + 54C > A, c.809 + 64C > A, c.3079 + 62 T > C, c.4002G > A and c.4126-45G > A.

We performed analysis of linkage disequilibrium (which is defined as non-random allelic association of different *loci*) to summarize our results for those 44 polymorphisms which were found to be consistent with the Hardy-Weinberg model. It turned out that, as expected, the r^2^ coefficient was most useful for analysis, and we detected polymorphic variant pairs with strong LD: our results are very similar even to those obtained for Japanese population [13]. It can be concluded on this basis that the specific linkages between particular variants are highly evolutionary conserved and occur in every population. Human genome is organized in haplotype blocks with high LD inside them, separated one from another by regions of low LD. Blocks show no evidence of historical recombination inside. This definition of haplotype blocks could be applied to 17 SNPs which had MAF higher than 0.05, which was the lower threshold in this analysis. We found four blocks and 12 common haplotypes including nine SNPs detected during this study. We determined htSNPs which should be sufficient to identify common haplotypes in population. Low haplotype diversity of *ABCC1* in Polish population is consistent with the conclusion of another study on Caucasians [15]. Although blocks for our results are small and the number of SNPs used for haplotype construction is low, the main core of blocks and their borders are retained across populations regardless of the blocks definitions used. Our blocks 1 and 2, usually connected as one in other studies, extend from intron 7 to intron 9. Our block 3 include SNPs from vicinity of exon 13 and block 4 contains SNPs from intron 28 to intron 30. It corresponds to block borders detected in other studies and suggest points of recombination which had taken place in intron 12 and in intron 27 or 28 [13–15].

Analysis using PolyPhen-2 which predicts the influence of detected non-synonymous SNPs on protein functionality suggested that six of them are suspect. However, each analysis is only a statistical calculation based on a full dataset and some experimental data do not support these results. Létourneau et al. [33] examined 10 missense SNPs of *ABCC1* and almost all of them, classified by PolyPhen-2 software as deleterious, affected neither expression nor functionality of the protein. A similar observation was also reported previously for the SNP c.2012G > T (p.Gly671Val), detected also by us. Although this substitution affects a residue near the highly conserved motif Walker A, it reduced mRNA expression but did not change transport properties of the protein, although PolyPhen-2 analysis clearly showed its damaging impact [34]. Recently, other data confirmed these observations and none of the amino acid substitutions: p.Arg633Gln, p.Gly671Val, p.Arg723Gln, detected also in our study, was found to change functionality of MRP1 transporter. Two substitutions altered drug resistance and inhibitor sensitivity and we found one of them, c.4002G > A (p.Ser1334=) [35].

On the other hand, there are other papers showing contrasting data for above-mentioned variants, proving their clinical association or experimental impact on protein functionality. Thus, it was demonstrated that variant c.2168G > A (p.Arg723Gln) reduced resistance activity of MRP1-overexpressing cells to drugs like daunorubicin, doxorubicin, etoposide, vinblastine and vincristine *in vitro* [36]. Although correlation between carrying this SNP and chemotherapy response was not observed in lung cancer patients [37], this variant was linked to the response to chemotherapy in patients with ovarian cancer [19]. Two other polymorphic variants detected in this study c.825 T > C (p.Val275=) and c.2012G > T (p.Gly671Val), were correlated with febrile neutropenia as an effect of FEC-induced hematological toxicity in breast cancer patients [38]. The major allele variant c.825 T > C (p.Val275=), in combination with another one c.*543C > T (beyond our scanning), was also associated with anthracycline-induced cardiotoxicity after chemotherapy in children with acute lymphoblastic leukemia [39]. The discrepancy observed in different studies on clinical significance of c.2012G > T (p.Gly671Val) and c.2168G > A (p.Arg723Gln) is of unclear origin. Cellular conditions *in vitro* in which mutants were expressed could be inadequate to reveal all their real functions, e.g. a change in mRNA structure could be more essential than the amino acid change caused by SNP [34]. Conseil and Cole [35] concluded from this discordance that comprehensive biochemical and pharmacological characterization of the respective MRP1 mutants should be performed to identify consequences of *ABCC1* SNPs on phenotype because these effects might be quite selective. Additional nucleic acid-based methods should be also used to clarify the impact of individual SNPs.

These examples show that only comprehensive examination of polymorphisms and studying their phenotypes can tell more about their real influence. Therefore, novel variants like c.596C > T (p.Ser199Leu), detected in this study and qualified *in silico* as damaging, need to be checked in an experimental investigation. Additionally, the fact that we found no novel mutation in important nucleotide binding motifs, nor any of the mutations identified previously *in vitro* as deleterious in NBD loops, proved that these highly conserved regions in the protein structure are unlikely to be affected by polymorphisms occurring in the healthy population.

## Conclusions

We scanned 30 exons and short flanking intron sequences of *ABCC1.* HRM is an efficient and sensitive method for scanning and genotyping polymorphic variants. We compared results of long-range scanning to targeted genotyping of selected SNPs showing that both methods are highly sensitive tools to study genetic variation. During our study we found 45 polymorphic variants, some of them rare and eight of them novel not previously reported. This is the first study about *ABCC1* genetic variation in the Polish population and we found some ethnic differences in frequency of genetic variants compared to other populations. These results need further complementation with a larger number of probands and new data including also introns and untranslated regions as sequences with polymorphic variants which may have clinical relevance as well.

## Additional files

Additional file 1:
**Supplemental materials for scanning study (include HRM plots and sequencing traces).** (DOCX 12174 kb) Additional file 2:
**Supplemental materials for targeted genotyping study (include HRM plots and sequencing traces).** (DOCX 5582 kb) Additional file 3:
**Supplemental materials for structural and functional disruption -** ***in silico*** **analysis using** **PolyPhen-2** **software.** (DOCX 13 kb) Additional file 4:
**Supplemental materials for linkage disequilibrium analysis (include pairwise plots with r** ^**2**^ **and |D'| parameters).** (DOCX 327 kb)

## Abbreviations

ABC transporters: ATP-binding cassette transporters
E_2_17βG: Estradiol-17-β-D-glucuronide
GSH: Glutathione
HRM: High resolution melting
htSNPs: Haplotype tag SNPs
HWE: Hardy Weinberg equilibrium
LD: Linkage disequilibrium
MAF: Minor allele frequency
MRP1: Multidrug resistance-associated protein 1
NBD: Nucleotide binding domain
TM: Transmembrane helice
TMD: Transmembrane domain
