# Supplementary material for: Genetic variation of the ABC transporter gene ABCC1 (Multidrug resistance protein 1 – MRP1) in the Polish population
Source: BMC Genet. 2015 Sep 23;16:114. doi: 10.1186/s12863-015-0271-3 (PMC4579605; doi:10.1186/s12863-015-0271-3)
Supplement: Additional file 1: — Supplemental materials for scanning study (include HRM plots and sequencing traces). (DOCX 12174 kb) [file 12863_2015_271_MOESM1_ESM.docx]

a) b)


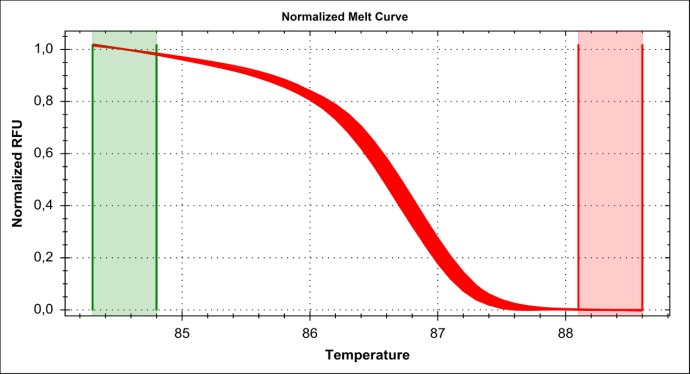

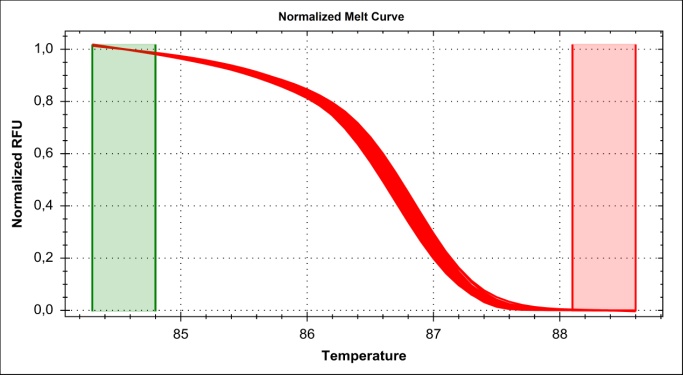


c)

**
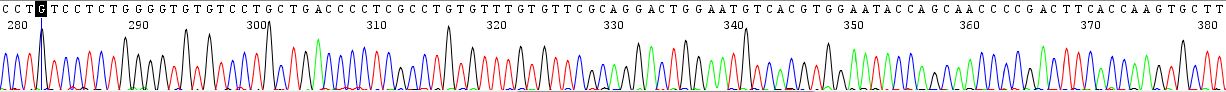
**

**
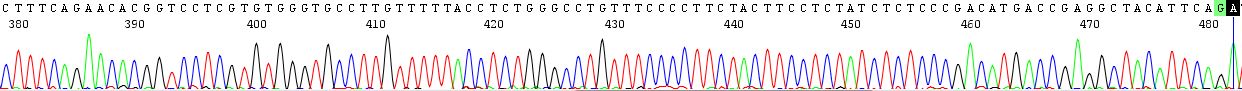
**

**Figure S1 Summary of *ABCC1* exon 2 scanning by HRM: a), b) melting plots for the first scanned area of the exon (NC_00016.10: 16007766 – 16007966, including primers). An example of sequencing trace for the samples: c) from the red clusters, no variants were detected (black boxes indicate the beginning and the end of the HRM product, including primers).**

**Table S1 Summary of *ABCC1* exon 2 (the first scanned area, NC_00016.10: 16007766 – 16007966) scanning by HRM. Scan rate is the ratio between positive clustered and verified samples to all the scanned samples.**

|  | Plate 1 | Plate 2 | Overall |
| --- | --- | --- | --- |
| Samples scanned | 95 | 95 | 190 |
| Melting clusters | 1 | 1 | 2 |
| Samples in cluster 1 (red) | 95 | 94 | 189 |
| Samples excluded | 0 | 1 | 1 |
| Samples verified by sequencing from cluster 1 | 3 | 3 | 6 |
| Scan rate | - | - | 0,9947 |

a) b)


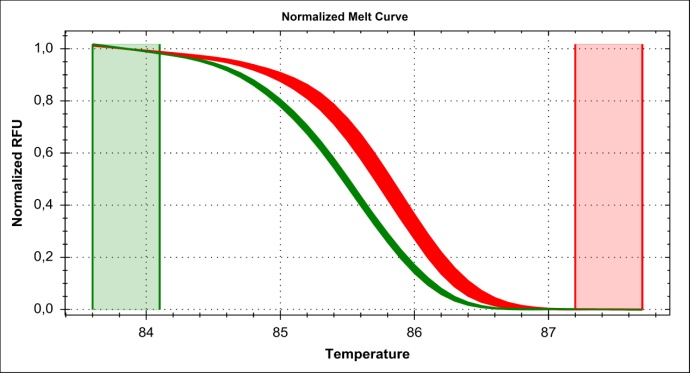

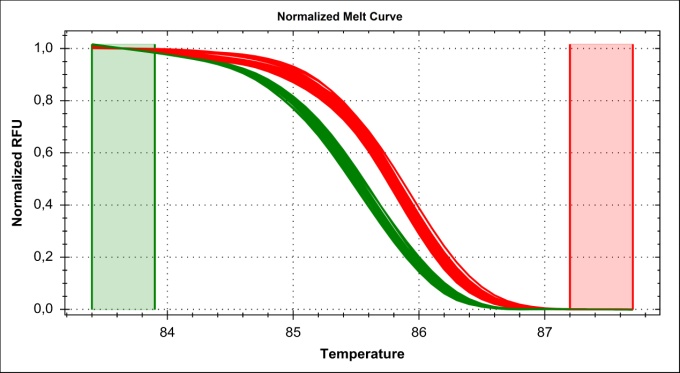


c)
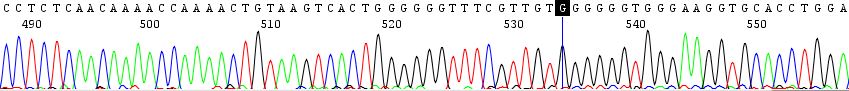


d)


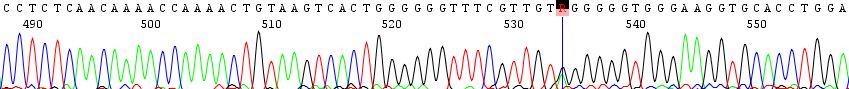


**Figure S2 Summary of *ABCC1* exon 2 scanning by HRM: a), b) melting plots for the second scanned area of the exon (NC_00016.10: 16007862 – 16008064, including primers). Examples of sequencing traces for the samples: c) from the red clusters (no variants), d) from the green clusters (heterozygous variant c.225+26G>A) (polymorphic base in black boxes).**

**Table S2 Summary of *ABCC1* exon 2 (the second scanned area, NC_00016.10: 16007862 – 16008064) scanning by HRM. Scan rate is the ratio between positive clustered and verified samples to all the scanned samples.**

|  | Plate 1 | Plate 2 | Overall |
| --- | --- | --- | --- |
| Samples scanned | 95 | 95 | 190 |
| Melting clusters | 2 | 2 | 4 |
| Samples in cluster 1 (red) | 86 | 78 | 164 |
| Samples in cluster 2 (green) | 9 | 16 | 25 |
| Samples excluded | 0 | 1 | 1 |
| Samples verified by sequencing from cluster 1 | 2 | 2 | 4 |
| Samples verified by sequencing from cluster 2 | 2 | 3 | 5 |
| Scan rate | - | - | 0,9947 |

1. b)

**
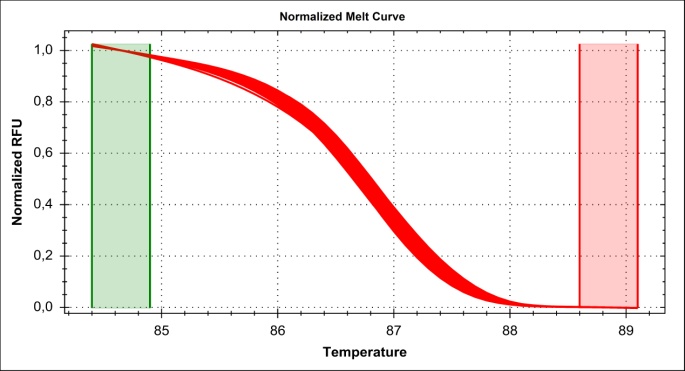

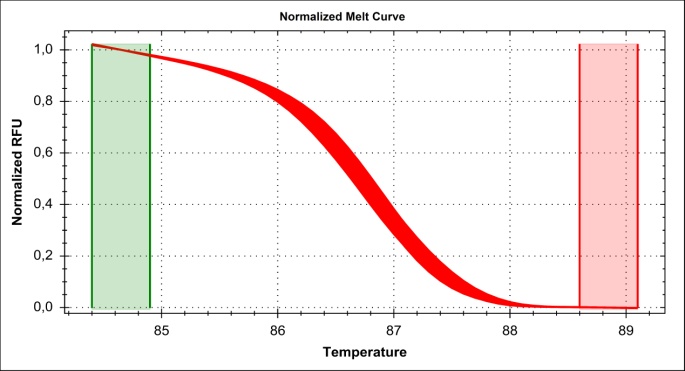
**

c)

**
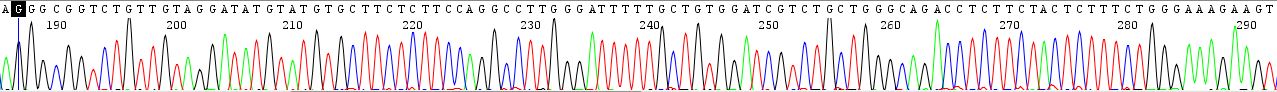
**

**
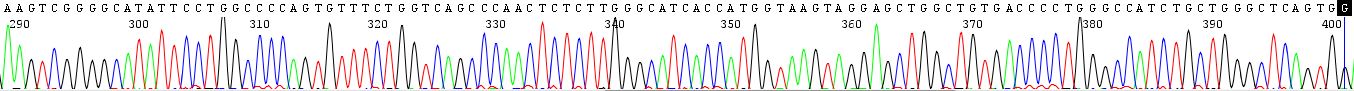
**

**Figure S3 Summary of *ABCC1* exon 3 scanning by HRM: a), b) melting plots for the whole exon scanned area (NC_00016.10: 16009736 – 16009950, including primers).An example of sequencing trace for the samples: c) from the red clusters, no variants were detected (black boxes indicate the beginning and the end of the HRM product, including primers).**

**Table S3 Summary of *ABCC1* exon 3 (NC_00016.10: 16009736 – 16009950) scanning by HRM. Scan rate is the ratio between positive clustered and verified samples to all the scanned samples.**

|  | Plate 1 | Plate 2 | Overall |
| --- | --- | --- | --- |
| Samples scanned | 95 | 95 | 190 |
| Melting clusters | 1 | 1 | 2 |
| Samples in cluster 1 (red) | 94 | 94 | 188 |
| Samples excluded | 1 | 1 | 2 |
| Samples verified by sequencing from cluster 1 | 3 | 3 | 6 |
| Scan rate | - | - | 0,9895 |

a) b)

**
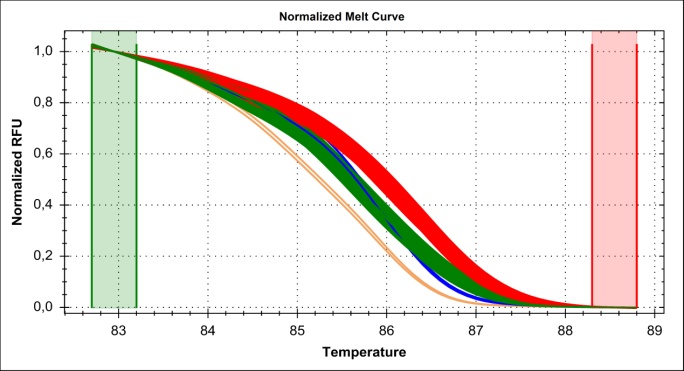

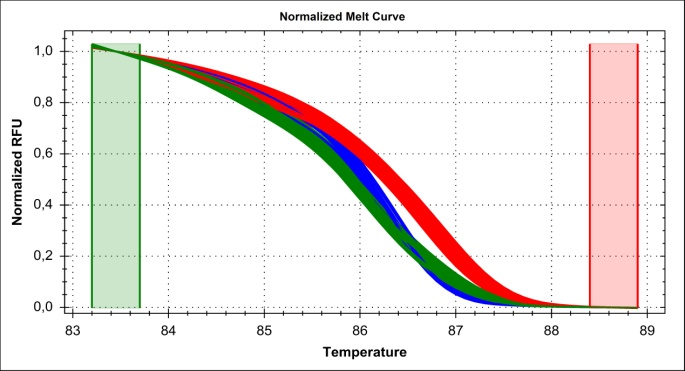
**

c)


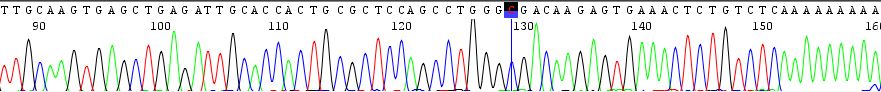


d)


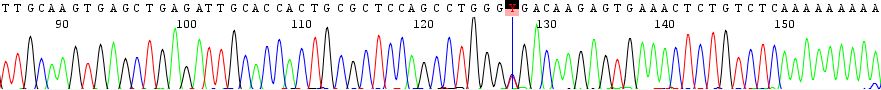


e)


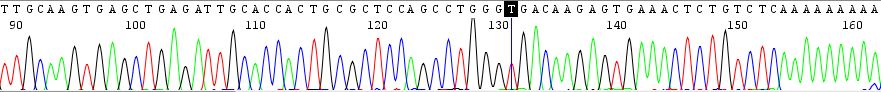


f)


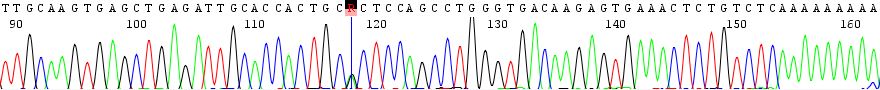


**Figure S4 Summary of *ABCC1* exon 4 scanning by HRM: a), b) melting plots for the first scanned area of the exon (NC_00016.10: 16014342 – 16014504, including primers). Examples of sequencing traces for the samples: c) from the red clusters (homozygous variant c.352-66T>C), d) from the green clusters (heterozygous variant c.352-66T>C), e) from the blue clusters (no variants), f) from the orange cluster (heterozygous variant c.352-79G>A, the novel one) (polymorphic bases in black boxes).**

**Table S4 Summary of *ABCC1* exon 4 (the first scanned area, NC_00016.10: 16014342 – 16014504) scanning by HRM. Scan rate is the ratio between positive clustered and verified samples to all the scanned samples.**

|  | Plate 1 | Plate 2 | Overall |
| --- | --- | --- | --- |
| Samples scanned | 95 | 95 | 190 |
| Melting clusters | 4 | 3 | 7 |
| Samples in cluster 1 (red) | 46 | 45 | 91 |
| Samples in cluster 2 (green) | 42 | 38 | 80 |
| Samples in cluster 3 (blue) | 3 | 11 | 14 |
| Samples in cluster 4 (orange) | 1 | - | 1 |
| Samples excluded | 3 | 1 | 4 |
| Samples verified by sequencing from cluster 1 | 2 | 2 | 4 |
| Samples verified by sequencing from cluster 2 | 2 | 2 | 4 |
| Samples verified by sequencing from cluster 3 | 3 | 2 | 5 |
| Samples verified by sequencing from cluster 4 | 1 | - | 1 |
| Scan rate | - | - | 0,9789 |

a) b)


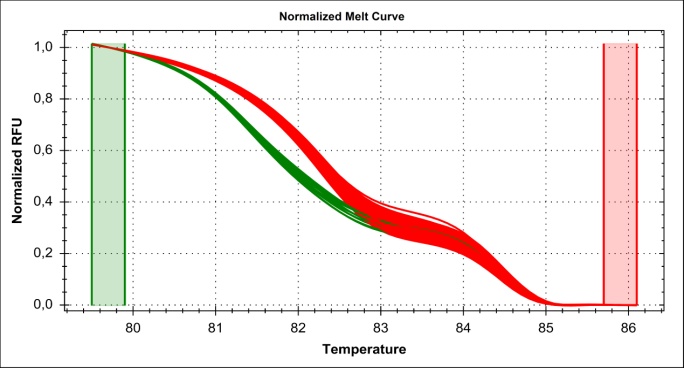

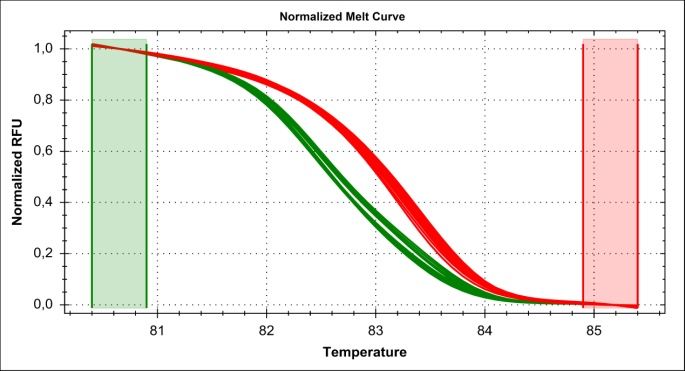


c)


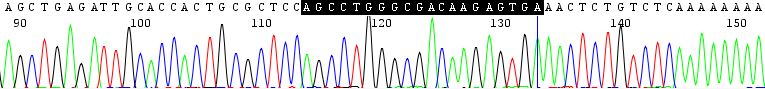


d)


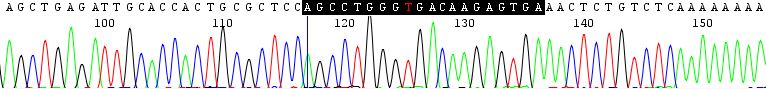


e)


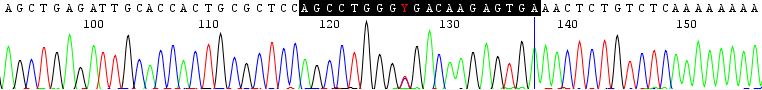


**Figure S5 Summary of *ABCC1* exon 4 scanning by HRM: a), b) melting plots for the second scanned area of the exon (NC_00016.10: 16014417 – 16014605, including primers), plots clustering inefficient with regard to the variant inside the forward primer region. Examples of sequencing traces for the samples: c) from the red clusters (homozygous variant detected in the forward primer region), d) from the green clusters (no variants), e) from the green clusters (heterozygous variant detected in the forward primer region) (sequence highlighted in black indicates the primer region, variants inside) - additional scanning area flanking this region was created (see above, Figure S4 and Table S4), no additional variants differentiating clusters were detected for this area.**

**Table S5 Summary of *ABCC1* exon 4 (the second scanned area, NC_00016.10: 16014417 – 16014605) scanning by HRM. Scan rate is the ratio between positive clustered samples to all the scanned samples.**

|  | Plate 1 | Plate 2 | Overall |
| --- | --- | --- | --- |
| Samples scanned | 95 | 95 | 190 |
| Melting clusters | 2 | 2 | 4 |
| Samples in cluster 1 (red) | 88 | 88 | 176 |
| Samples in cluster 2 (green) | 6 | 7 | 13 |
| Samples excluded | 1 | 0 | 1 |
| Samples verified by sequencing from cluster 1 | 2 | 2 | 4 |
| Samples verified by sequencing from cluster 2 | 2 | 5 | 7 |
| Scan rate | - | - | 0,9947 |

a) b)


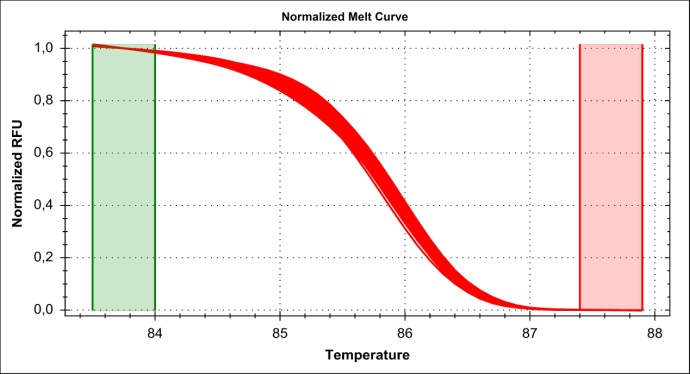

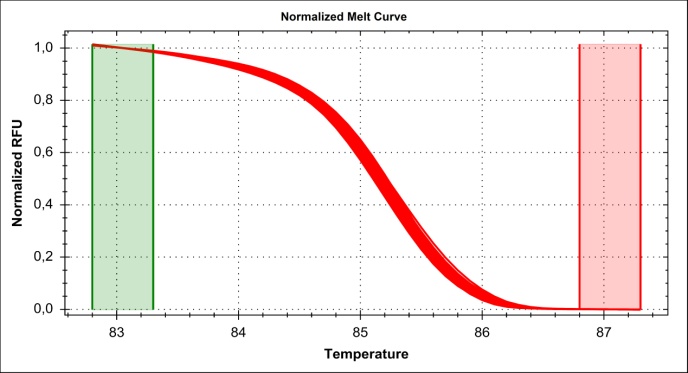


c)


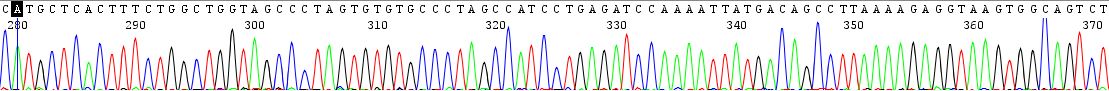


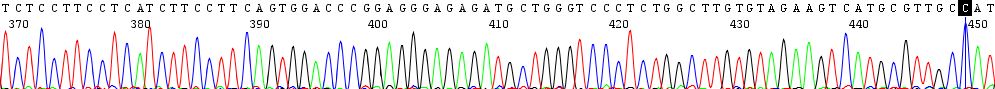


**Figure S6 Summary of *ABCC1* exon 4 scanning by HRM: a), b) melting plots for the third scanned area of the exon (NC_00016.10: 16014551 – 16014720, including primers). An example of sequencing trace for the samples: c) from the red clusters, no variants were detected (black boxes indicate the beginning and the end of the HRM product, including primers).**

**Table S6 Summary of *ABCC1* exon 4 (the third scanned area, NC_00016.10: 16014551 – 16014720) scanning by HRM. Scan rate is the ratio between positive clustered and verified samples to all the scanned samples.**

|  | Plate 1 | Plate 2 | Overall |
| --- | --- | --- | --- |
| Samples scanned | 95 | 95 | 190 |
| Melting clusters | 1 | 1 | 2 |
| Samples in cluster 1 (red) | 94 | 94 | 188 |
| Samples excluded | 1 | 1 | 2 |
| Samples verified by sequencing from cluster 1 | 3 | 3 | 6 |
| Scan rate | - | - | 0,9895 |

a) b)


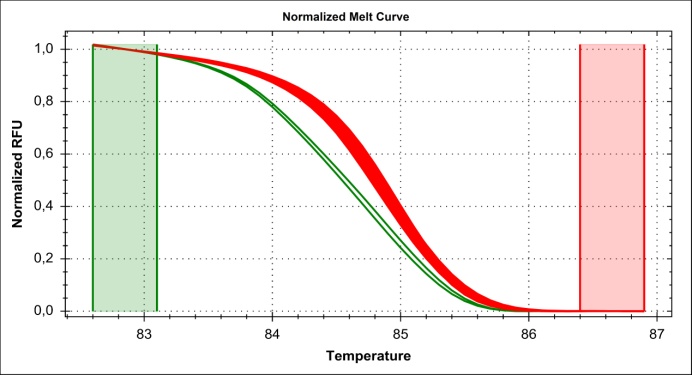

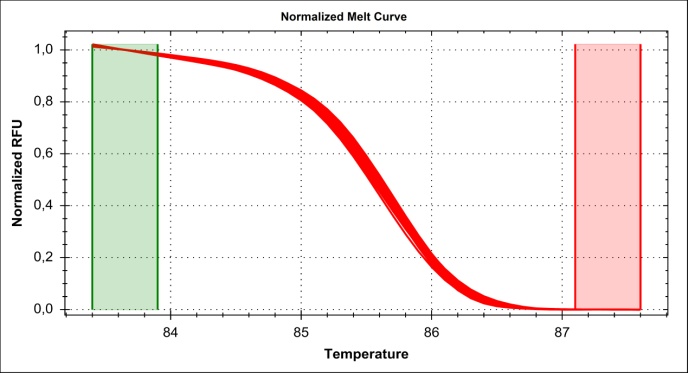


c)


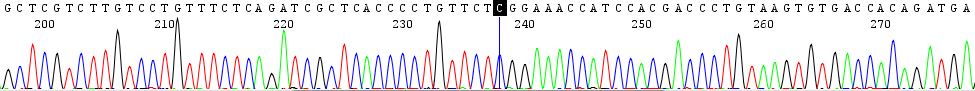


d)


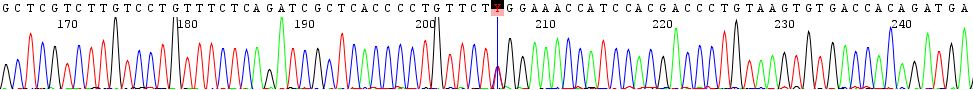


**Figure S7 Summary of *ABCC1* exon 5 scanning by HRM: a), b) melting plots for the whole exon scanned area (NC_00016.10: 16016449 – 16016660, including primers). Examples of sequencing traces for the samples: c) from the red clusters (no variants), d) from the green cluster (heterozygous variant c.596C>T, the novel one) (polymorphic base in black boxes).**

**Table S7 Summary of *ABCC1* exon 5 (NC_00016.10: 16016449 – 16016660) scanning by HRM. Scan rate is the ratio between positive clustered and verified samples to all the scanned samples.**

|  | Plate 1 | Plate 2 | Overall |
| --- | --- | --- | --- |
| Samples scanned | 95 | 95 | 190 |
| Melting clusters | 2 | 1 | 3 |
| Samples in cluster 1 (red) | 93 | 93 | 186 |
| Samples in cluster 2 (green) | 1 | - | 1 |
| Samples excluded | 1 | 2 | 3 |
| Samples verified by sequencing from cluster 1 | 2 | 3 | 5 |
| Samples verified by sequencing from cluster 2 | 1 | - | 1 |
| Scan rate | - | - | 0,9842 |

a) b)


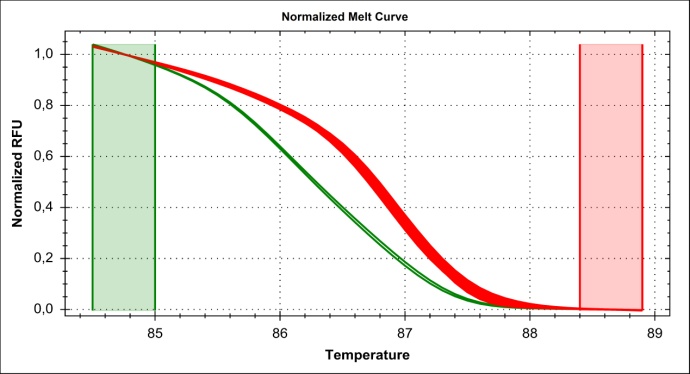

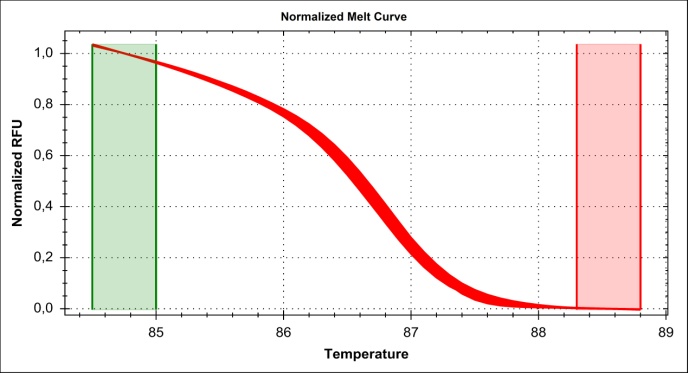


c)


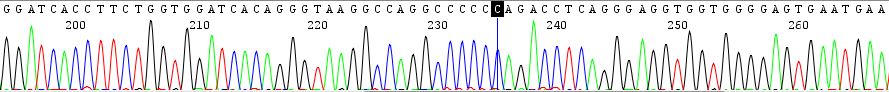


d)


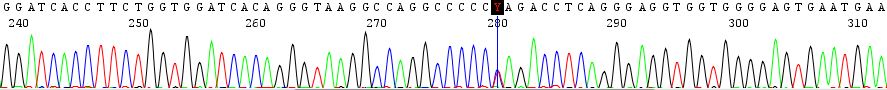


**Figure S8 Summary of *ABCC1* exon 6 scanning by HRM: a), b) melting plots for the whole exon scanned area (NC_00016.10: 16033069 – 16033220, including primers). Examples of sequencing traces for the samples: c) from the red clusters (no variants), d) from the green cluster (heterozygous variant c.677+17C>T) (polymorphic base in black boxes).**

**Table S8 Summary of *ABCC1* exon 6 (NC_00016.10: 16033069 – 16033220) scanning by HRM. Scan rate is the ratio between positive clustered and verified samples to all the scanned samples.**

|  | Plate 1 | Plate 2 | Overall |
| --- | --- | --- | --- |
| Samples scanned | 95 | 95 | 190 |
| Melting clusters | 2 | 1 | 3 |
| Samples in cluster 1 (red) | 93 | 94 | 187 |
| Samples in cluster 2 (green) | 1 | - | 1 |
| Samples excluded | 1 | 1 | 2 |
| Samples verified by sequencing from cluster 1 | 2 | 3 | 5 |
| Samples verified by sequencing from cluster 2 | 1 | - | 1 |
| Scan rate | - | - | 0,9895 |

a) b)


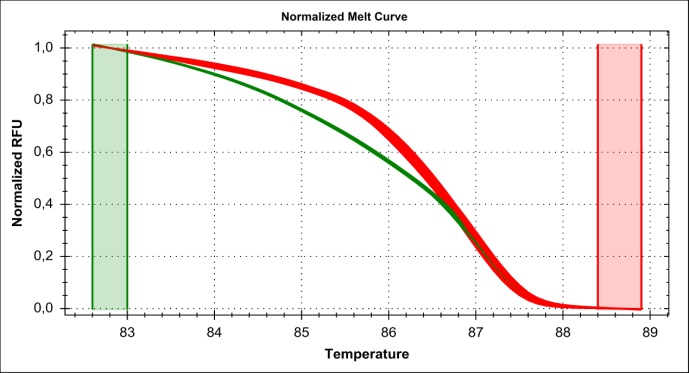

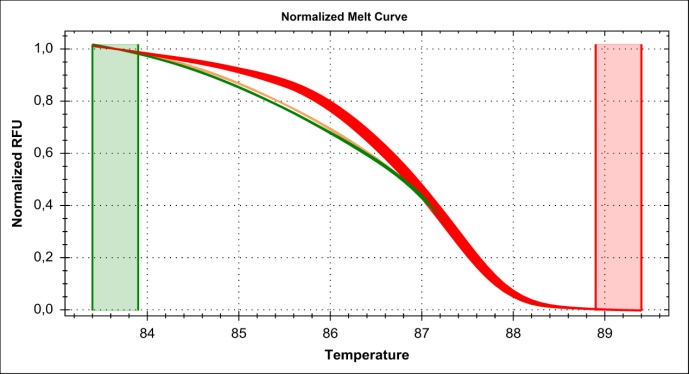


c)

**
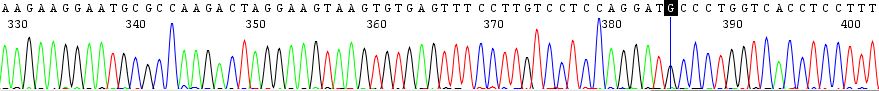
**

d)


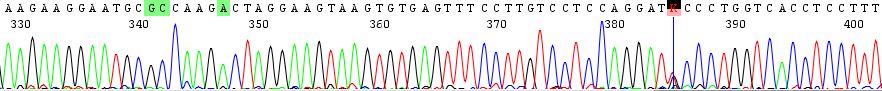


e)


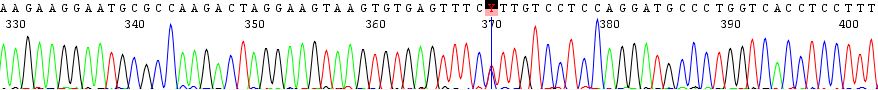


**Figure S9 Summary of *ABCC1* exon 7 scanning by HRM: a), b) melting plots for the first scanned area of the exon (NC_00016.10: 16036418 – 16036657, including primers). Examples of sequencing traces for the samples: c) from the red clusters (no variants), d) from the green clusters (heterozygous variant c.809+31G>T), e) from the orange cluster (heterozygous variant c.809+16C>T, the novel one) (polymorphic bases in black boxes).**

**Table S9 Summary of *ABCC1* exon 7 (the first scanned area, NC_00016.10: 16036418 – 16036657) scanning by HRM. Scan rate is the ratio between positive clustered and verified samples to all the scanned samples.**

|  | Plate 1 | Plate 2 | Overall |
| --- | --- | --- | --- |
| Samples scanned | 95 | 95 | 190 |
| Melting clusters | 2 | 3 | 5 |
| Samples in cluster 1 (red) | 92 | 91 | 183 |
| Samples in cluster 2 (green) | 3 | 2 | 5 |
| Samples in cluster 3 (orange) | - | 1 | 1 |
| Samples excluded | 0 | 1 | 1 |
| Samples verified by sequencing from cluster 1 | 3 | 2 | 5 |
| Samples verified by sequencing from cluster 2 | 3 | 2 | 5 |
| Samples verified by sequencing from cluster 3 | - | 1 | 1 |
| Scan rate | - | - | 0,9947 |

a) b)


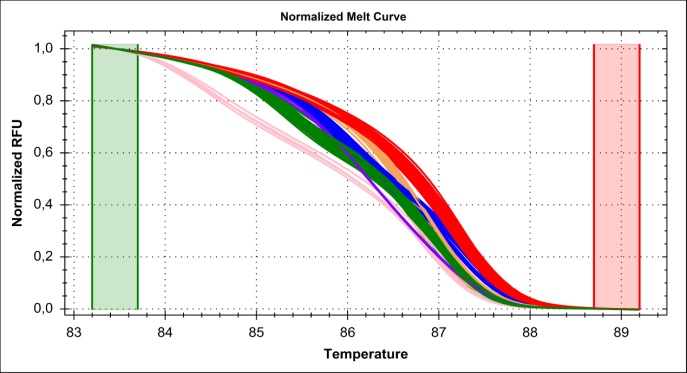

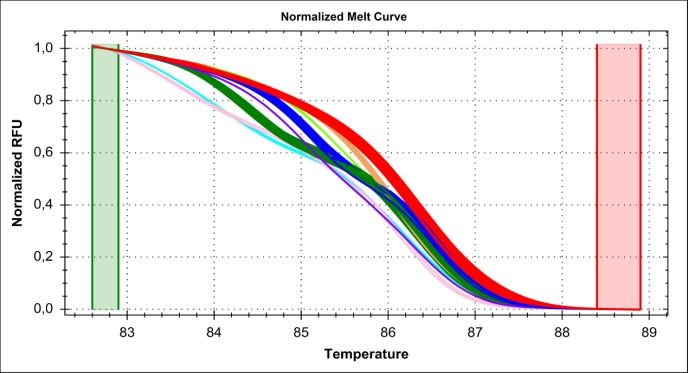


c)


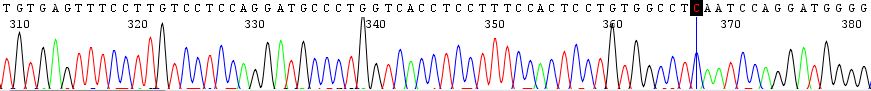


d)


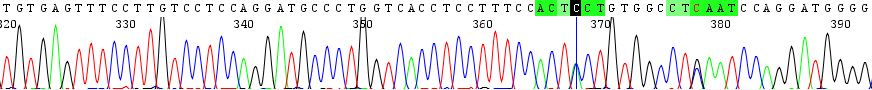


e)


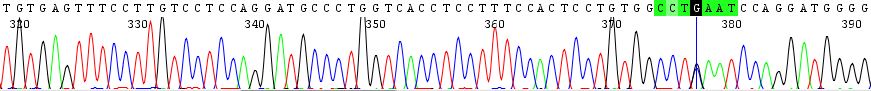


f)


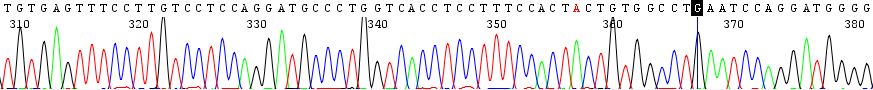


g)


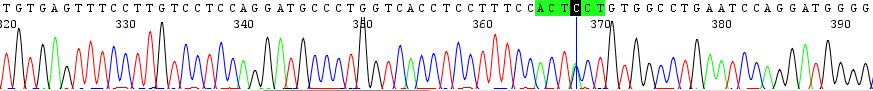


h)


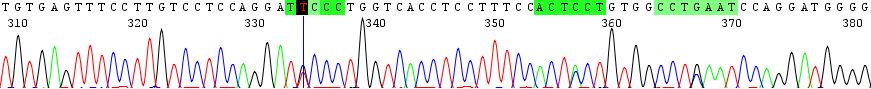


i)


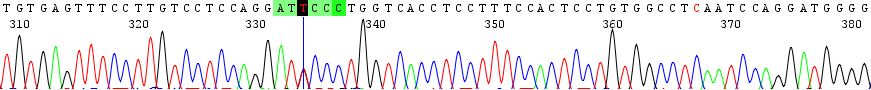


j)


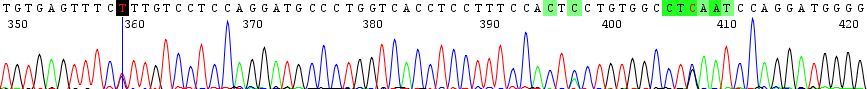


**Figure S10 Summary of *ABCC1* exon 7 scanning by HRM: a), b) melting plots for the second scanned area of the exon (NC_00016.10: 16036544 – 16036727, including primers). Examples of sequencing traces for the samples: c) from the red clusters (no variants), d) from the green clusters (heterozygous variants c.809+54C>A (in black box) and c.809+64C>G), e) from the blue clusters (heterozygous variant c.809+64C>G (in black box)), f) from the orange clusters (homozygous variants c.809+54C>A and c.809+64C>G (in black box)), g) from the lime cluster (heterozygous variant c.809+54C>A (in black box) and homozygous variant c.809+64C>G), h) from the pink clusters (heterozygous variants c.809+31G>T (in black box) and c.809+54C>A and c.809+64C>G), i) from the violet clusters (heterozygous variant c.809+31G>T (in black box)), j) from the cyan cluster (heterozygous variants c.809+16C>T (in black box, the novel one) and c.809+54C>A and c.809+64C>G).**

**Table S10 Summary of *ABCC1* exon 7 (the second scanned area, NC_00016.10: 16036544 – 16036727) scanning by HRM. Scan rate is the ratio between positive clustered and verified samples to all the scanned samples.**

|  | Plate 1 | Plate 2 | Overall |
| --- | --- | --- | --- |
| Samples scanned | 95 | 95 | 190 |
| Melting clusters | 6 | 8 | 14 |
| Samples in cluster 1 (red) | 46 | 36 | 82 |
| Samples in cluster 2 (green) | 24 | 34 | 58 |
| Samples in cluster 3 (blue) | 14 | 14 | 28 |
| Samples in cluster 4 (orange) | 7 | 4 | 11 |
| Samples in cluster 5 (lime) | - | 3 | 3 |
| Samples in cluster 6 (pink) | 2 | 1 | 3 |
| Samples in cluster 7 (violet) | 1 | 1 | 2 |
| Samples in cluster 8 (cyan) | - | 1 | 1 |
| Samples excluded | 1 | 1 | 2 |
| Samples verified by sequencing from cluster 1 | 2 | 2 | 4 |
| Samples verified by sequencing from cluster 2 | 2 | 2 | 4 |
| Samples verified by sequencing from cluster 3 | 2 | 2 | 4 |
| Samples verified by sequencing from cluster 4 | 2 | 2 | 4 |
| Samples verified by sequencing from cluster 5 | - | 3 | 3 |
| Samples verified by sequencing from cluster 6 | 2 | 1 | 3 |
| Samples verified by sequencing from cluster 7 | 1 | 1 | 2 |
| Samples verified by sequencing from cluster 8 | - | 1 | 1 |
| Scan rate | - | - | 0,9895 |

a) b)


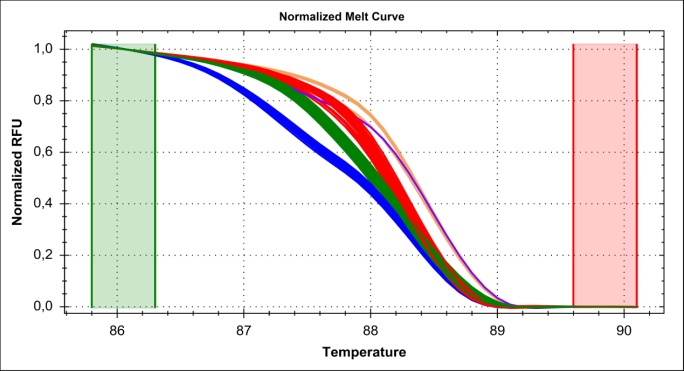

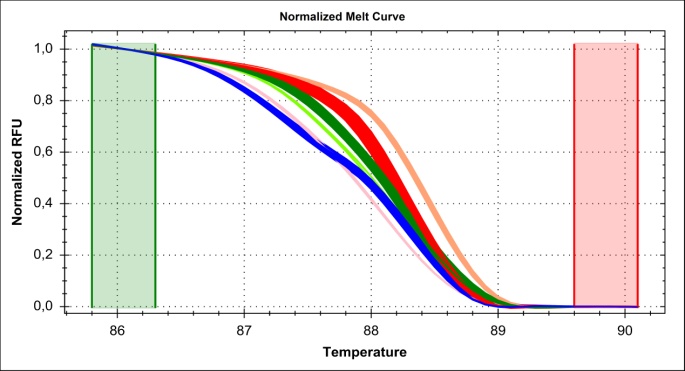


c)


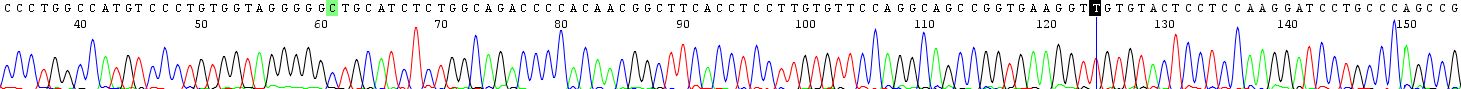


d)


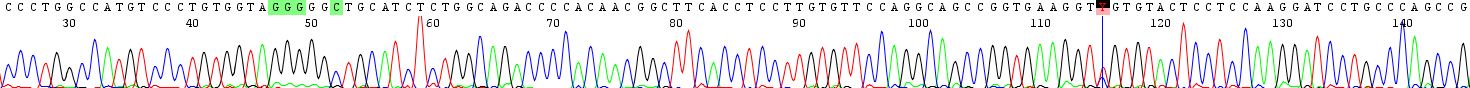


e)


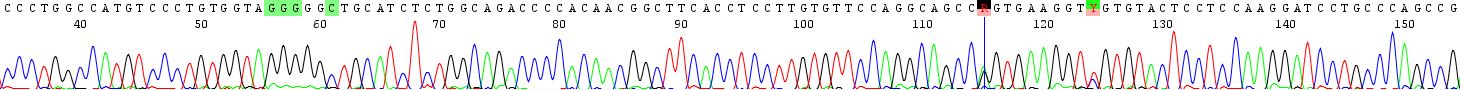


f)


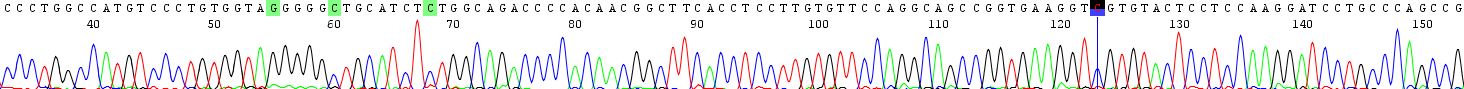


g)


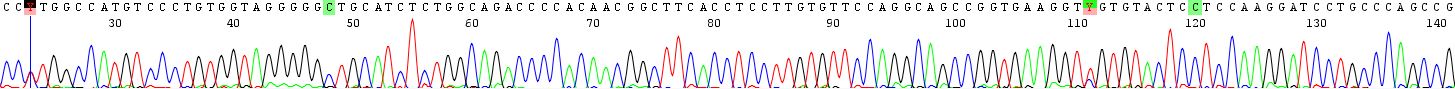


h)


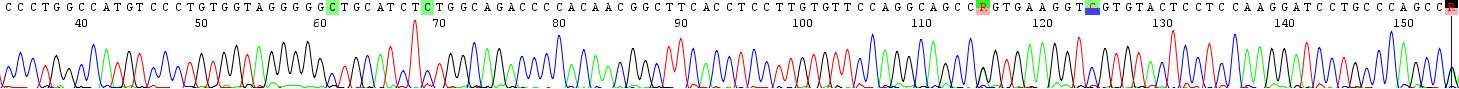


i)


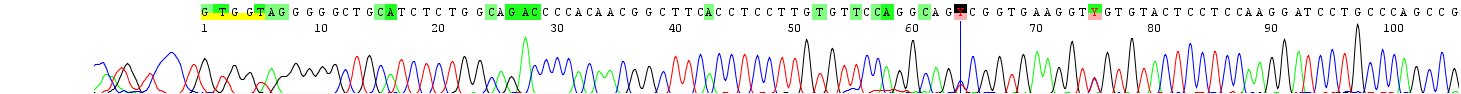


**Figure S11 Summary of *ABCC1* exon 8 scanning by HRM: a), b) melting plots for the first scanned area of the exon (NC_00016.10: 16044356 – 16044574, including primers). Examples of sequencing traces for the samples: c) from the red clusters (no variants), d) from the green clusters (heterozygous variant c.825T>C (in black box)), e) from the blue clusters (heterozygous variants c.816G>A (in black box) and c.825T>C), f) from the orange clusters (homozygous variant c.825T>C (in black box)), g) from the lime cluster (heterozygous variants c.810-73C>T (in black box) and c.825T>C), h) from the pink cluster (heterozygous variant c.816G>A and homozygous variant c.825T>C and heterozygous variant c.855G>A (the novel one, in black box)), i) from the violet cluster (heterozygous variants c.814C>T (in black box) and c.825T>C).**

**Table S11 Summary of *ABCC1* exon 8 (the first scanned area, NC_00016.10: 16044356 – 16044574) scanning by HRM. Scan rate is the ratio between positive clustered and verified samples to all the scanned samples.**

|  | Plate 1 | Plate 2 | Overall |
| --- | --- | --- | --- |
| Samples scanned | 95 | 95 | 190 |
| Melting clusters | 5 | 6 | 11 |
| Samples in cluster 1 (red) | 44 | 40 | 84 |
| Samples in cluster 2 (green) | 40 | 35 | 75 |
| Samples in cluster 3 (blue) | 7 | 8 | 15 |
| Samples in cluster 4 (orange) | 2 | 9 | 11 |
| Samples in cluster 5 (lime) | - | 1 | 1 |
| Samples in cluster 6 (pink) | - | 1 | 1 |
| Samples in cluster 7 (violet) | 1 | - | 1 |
| Samples excluded | 1 | 1 | 2 |
| Samples verified by sequencing from cluster 1 | 2 | 2 | 4 |
| Samples verified by sequencing from cluster 2 | 2 | 2 | 4 |
| Samples verified by sequencing from cluster 3 | 2 | 2 | 4 |
| Samples verified by sequencing from cluster 4 | 2 | 2 | 4 |
| Samples verified by sequencing from cluster 5 | - | 1 | 1 |
| Samples verified by sequencing from cluster 6 | - | 1 | 1 |
| Samples verified by sequencing from cluster 7 | 1 | - | 1 |
| Scan rate | - | - | 0,9895 |

a) b)


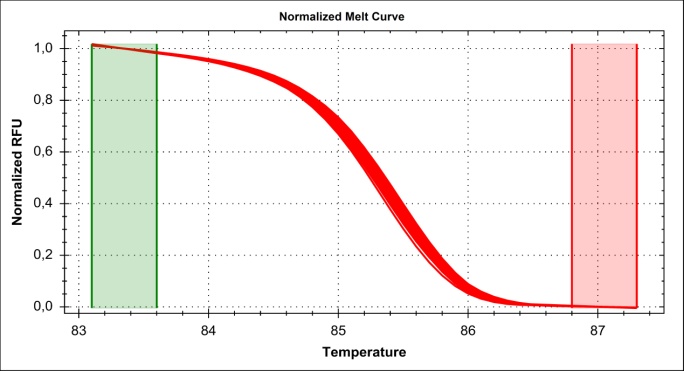

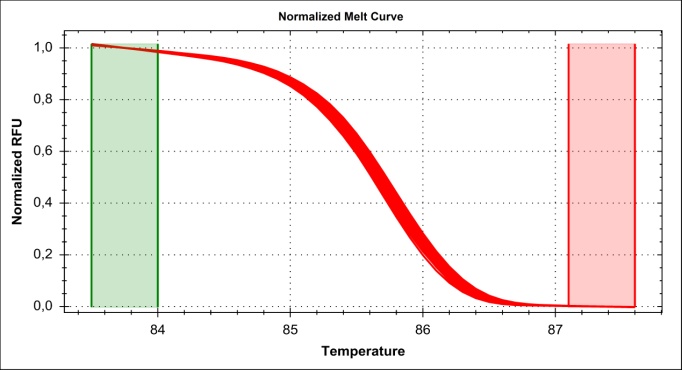


c)


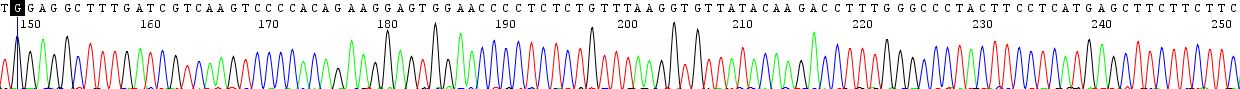


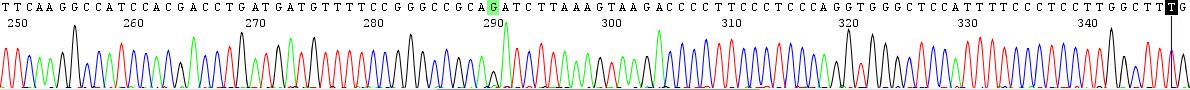


**Figure S12 Summary of *ABCC1* exon 8 scanning by HRM: a), b) melting plots for the second scanned area of the exon (NC_00016.10: 16044531 – 16044729, including primers). An example of sequencing trace for the samples: c) from the red clusters, no variants were detected (black boxes indicate the beginning and the end of the HRM product, including primers).**

**Table S12 Summary of *ABCC1* exon 8 (the second scanned area, NC_00016.10: 16044531 – 16044729) scanning by HRM. Scan rate is the ratio between positive clustered and verified samples to all the scanned samples.**

|  | Plate 1 | Plate 2 | Overall |
| --- | --- | --- | --- |
| Samples scanned | 95 | 95 | 190 |
| Melting clusters | 1 | 1 | 2 |
| Samples in cluster 1 (red) | 95 | 94 | 189 |
| Samples excluded | 0 | 1 | 1 |
| Samples verified by sequencing from cluster 1 | 3 | 3 | 6 |
| Scan rate | - | - | 0,9947 |

a) b)


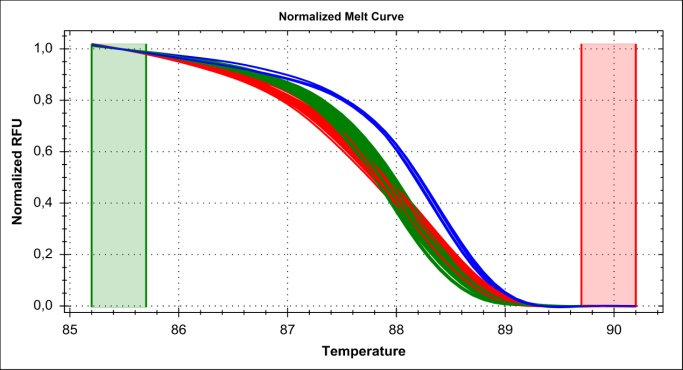

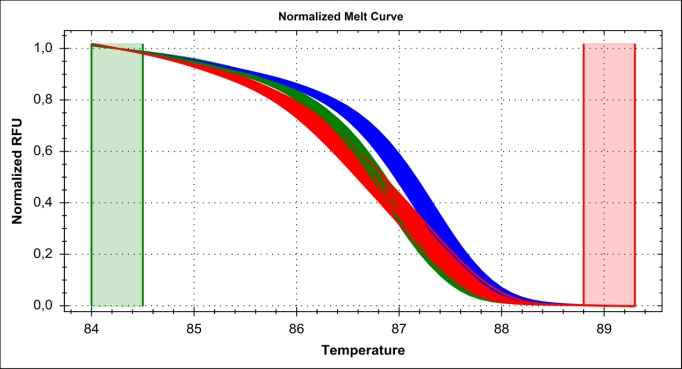


c)


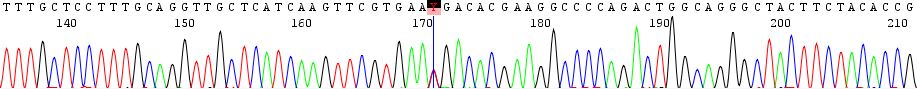


d)


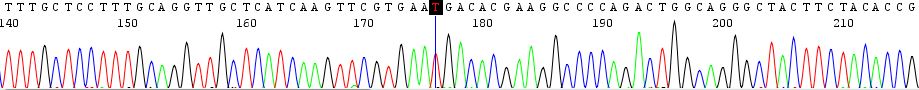


e)


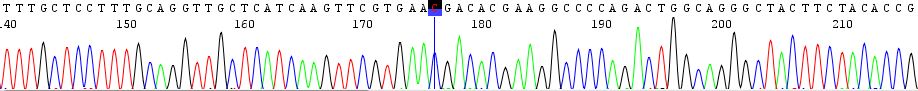


**Figure S13 Summary of *ABCC1* exon 9 scanning by HRM: a), b) melting plots for the first scanned area of the exon (NC_00016.10: 16045802 – 16045966, including primers). Examples of sequencing traces for the samples: c) from the red clusters (heterozygous variant c.1062T>C), d) from the green clusters (no variants), e) from the blue clusters (homozygous variant c.1062T>C) (polymorphic base in black boxes).**

**Table S13 Summary of *ABCC1* exon 9 (the first scanned area, NC_00016.10: 16045802 – 16045966) scanning by HRM. Scan rate is the ratio between positive clustered and verified samples to all the scanned samples.**

|  | Plate 1 | Plate 2 | Overall |
| --- | --- | --- | --- |
| Samples scanned | 95 | 95 | 190 |
| Melting clusters | 3 | 3 | 6 |
| Samples in cluster 1 (red) | 47 | 44 | 91 |
| Samples in cluster 2 (green) | 44 | 34 | 78 |
| Samples in cluster 3 (blue) | 2 | 14 | 16 |
| Samples excluded | 2 | 3 | 5 |
| Samples verified by sequencing from cluster 1 | 2 | 2 | 4 |
| Samples verified by sequencing from cluster 2 | 2 | 2 | 4 |
| Samples verified by sequencing from cluster 3 | 2 | 2 | 4 |
| Scan rate | - | - | 0,9737 |

a) b)


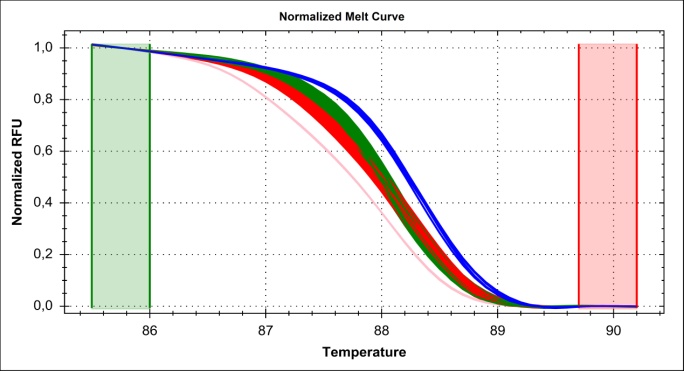

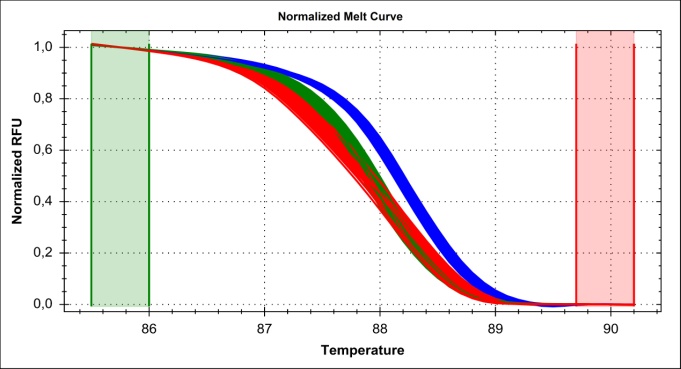


c)


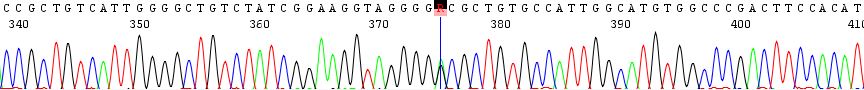


d)


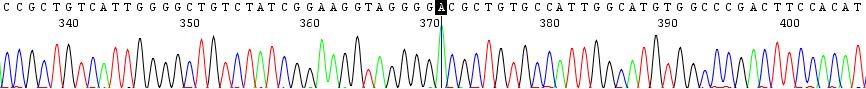


e)


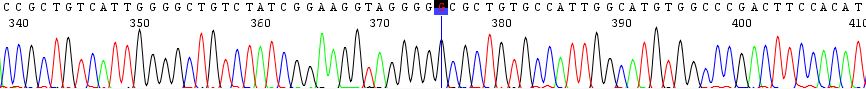


f)


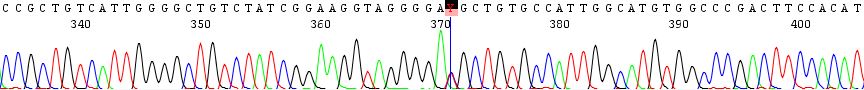


**Figure S14 Summary of *ABCC1* exon 9 scanning by HRM: a), b) melting plots for the second scanned area of the exon (NC_00016.10: 16045891 – 16046065, including primers). Examples of sequencing traces for the samples: c) from the red clusters (heterozygous variant c.1218+8A>G (in black box)), d) from the green clusters (no variants), e) from the blue clusters (homozygous variant c.1218+8A>G (in black box), f) from the pink cluster (heterozygous variant c.1218+9C>T, the novel one (in black box)).**

**Table S14 Summary of *ABCC1* exon 9 (the second scanned area, NC_00016.10: 16045891 – 16046065) scanning by HRM. Scan rate is the ratio between positive clustered and verified samples to all the scanned samples.**

|  | Plate 1 | Plate 2 | Overall |
| --- | --- | --- | --- |
| Samples scanned | 95 | 95 | 190 |
| Melting clusters | 4 | 3 | 7 |
| Samples in cluster 1 (red) | 47 | 44 | 91 |
| Samples in cluster 2 (green) | 43 | 38 | 81 |
| Samples in cluster 3 (blue) | 4 | 12 | 16 |
| Samples in cluster 4 (pink) | 1 | - | 1 |
| Samples excluded | 0 | 1 | 1 |
| Samples verified by sequencing from cluster 1 | 1 | 2 | 3 |
| Samples verified by sequencing from cluster 2 | 2 | 2 | 4 |
| Samples verified by sequencing from cluster 3 | 2 | 2 | 4 |
| Samples verified by sequencing from cluster 4 | 1 | - | 1 |
| Scan rate | - | - | 0,9947 |

a) b)


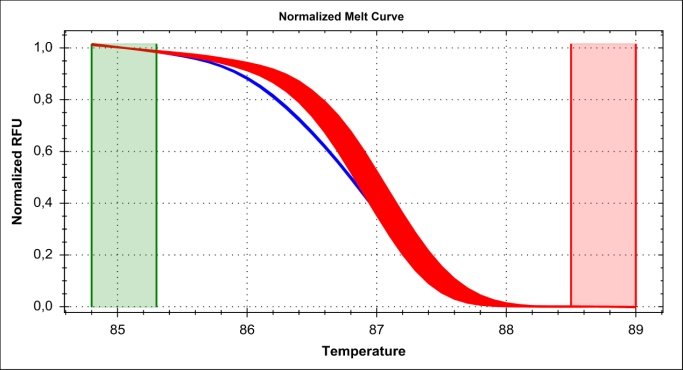

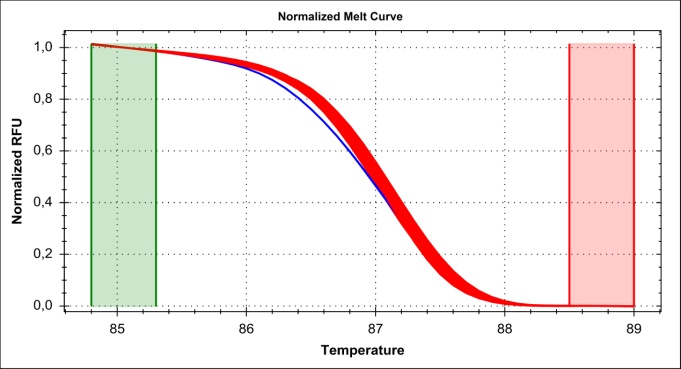


c)


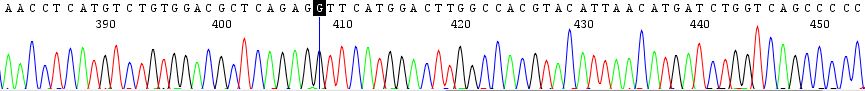


d)


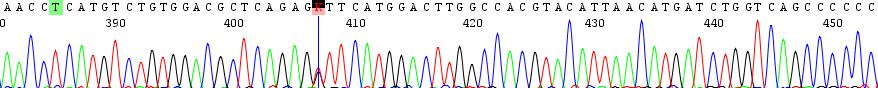


**Figure S15 Summary of *ABCC1* exon 10 scanning by HRM: a), b) melting plots for the first scanned area of the exon (NC_00016.10: 16048095 – 16048301, including primers). Examples of sequencing traces for the samples: c) from the red clusters (no variants), d) from the blue clusters (heterozygous variant c.1299G>T) (polymorphic base in black boxes).**

**Table S15 Summary of *ABCC1* exon 10 (the first scanned area, NC_00016.10: 16048095 – 16048301) scanning by HRM. Scan rate is the ratio between positive clustered and verified samples to all the scanned samples.**

|  | Plate 1 | Plate 2 | Overall |
| --- | --- | --- | --- |
| Samples scanned | 95 | 95 | 190 |
| Melting clusters | 2 | 2 | 4 |
| Samples in cluster 1 (red) | 93 | 93 | 186 |
| Samples in cluster 2 (blue) | 1 | 1 | 2 |
| Samples excluded | 1 | 1 | 2 |
| Samples verified by sequencing from cluster 1 | 2 | 4 | 6 |
| Samples verified by sequencing from cluster 2 | 1 | 1 | 2 |
| Scan rate | - | - | 0,9895 |

a) b)


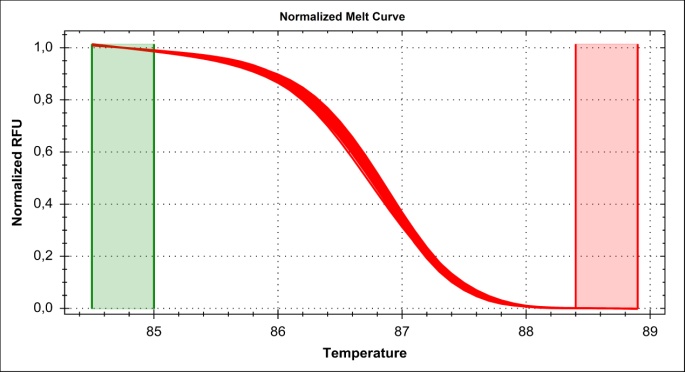

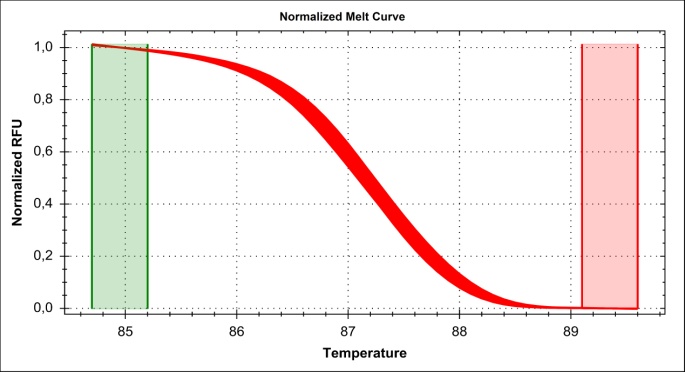


c)


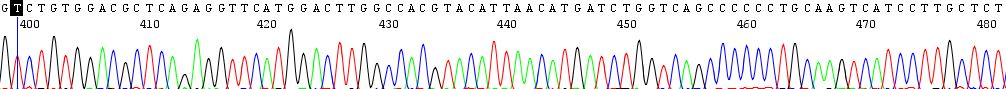


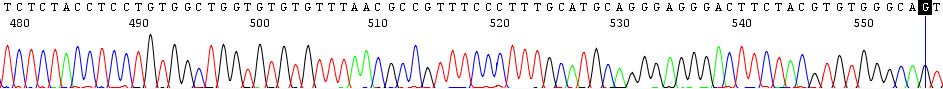


**Figure S16 Summary of *ABCC1* exon 10 scanning by HRM: a), b) melting plots for the second scanned area of the exon (NC_00016.10: 16048205 – 16048361, including primers). An example of sequencing trace for the samples: c) from the red clusters, no variants were detected (black boxes indicate the beginning and the end of the HRM product, including primers).**

**Table S16 Summary of *ABCC1* exon 10 (the second scanned area, NC_00016.10: 16048205 – 16048361) scanning by HRM. Scan rate is the ratio between positive clustered and verified samples to all the scanned samples.**

|  | Plate 1 | Plate 2 | Overall |
| --- | --- | --- | --- |
| Samples scanned | 95 | 95 | 190 |
| Melting clusters | 1 | 1 | 2 |
| Samples in cluster 1 (red) | 94 | 93 | 187 |
| Samples excluded | 1 | 2 | 3 |
| Samples verified by sequencing from cluster 1 | 2 | 3 | 5 |
| Scan rate | - | - | 0,9842 |

a) b)


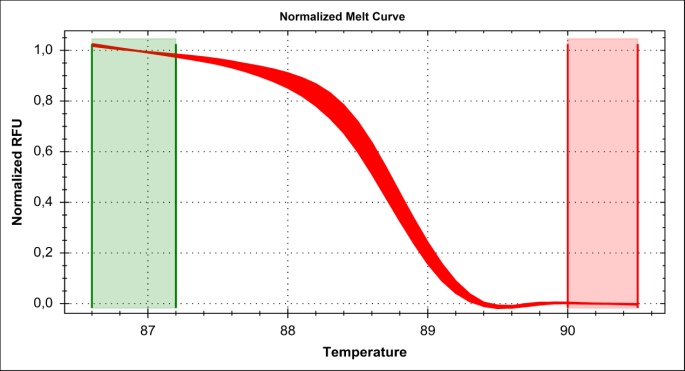

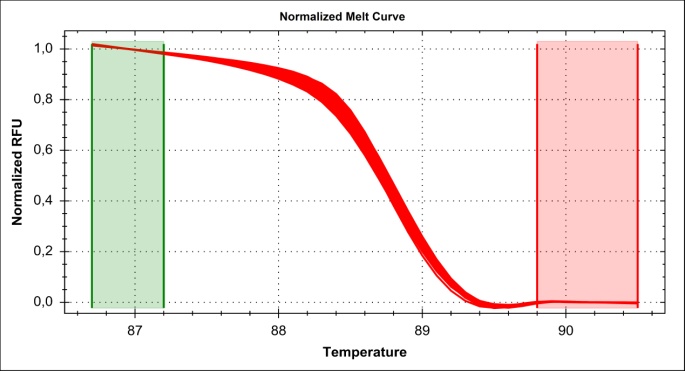


c)


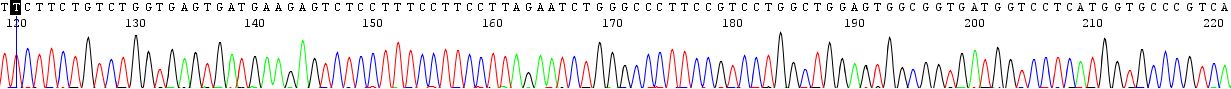


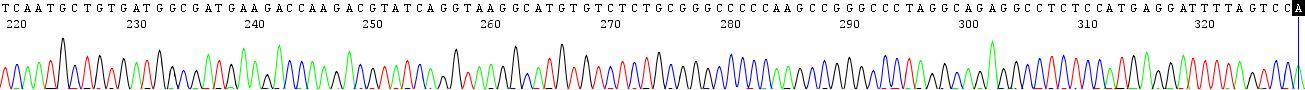


**Figure S17 Summary of *ABCC1* exon 11 scanning by HRM: a), b) melting plots for the whole exon scanned area (NC_00016.10: 16052680 – 16052888, including primers). An example of the sequencing trace for the samples: c) from the red clusters, no variants were detected (black boxes indicate the beginning and the end of the HRM product, including primers).**

**Table S17 Summary of *ABCC1* exon 11 (NC_00016.10: 16052680 – 16052888) scanning by HRM. Scan rate is the ratio between positive clustered and verified samples to all the scanned samples.**

|  | Plate 1 | Plate 2 | Overall |
| --- | --- | --- | --- |
| Samples scanned | 95 | 95 | 190 |
| Melting clusters | 1 | 1 | 2 |
| Samples in cluster 1 (red) | 93 | 95 | 188 |
| Samples excluded | 2 | 0 | 2 |
| Samples verified by sequencing from cluster 1 | 3 | 4 | 7 |
| Scan rate | - | - | 0,9895 |

a) b)


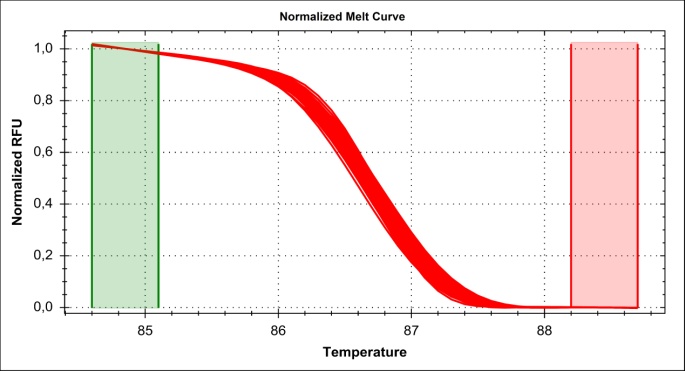

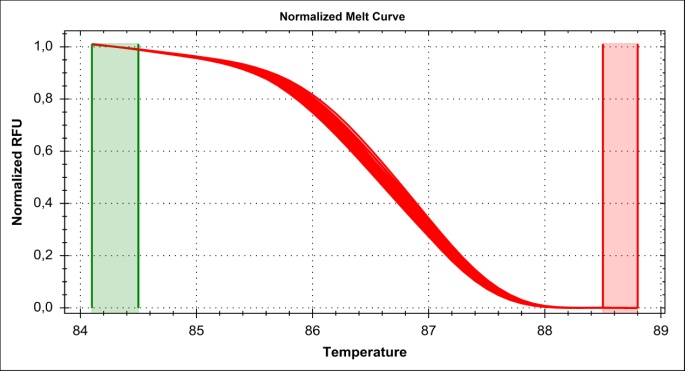


c)


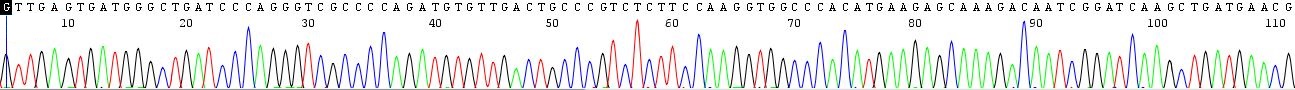


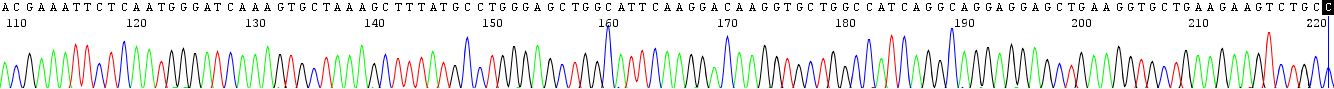


**Figure S18 Summary of *ABCC1* exon 12 scanning by HRM: a), b) melting plots for the first scanned area of the exon (NC_00016.10: 16056031 – 16056247, including primers). An example of sequencing trace for the samples: c) from the red clusters, no variants were detected (black boxes indicate the beginning and the end of the HRM product, including primers).**

**Table S18 Summary of *ABCC1* exon 12 (the first scanned area, NC_00016.10: 16056031 – 16056247) scanning by HRM. Scan rate is the ratio between positive clustered and verified samples to all the scanned samples.**

|  | Plate 1 | Plate 2 | Overall |
| --- | --- | --- | --- |
| Samples scanned | 95 | 95 | 190 |
| Melting clusters | 1 | 1 | 2 |
| Samples in cluster 1 (red) | 95 | 94 | 189 |
| Samples excluded | 0 | 1 | 1 |
| Samples verified by sequencing from cluster 1 | 3 | 3 | 6 |
| Scan rate | - | - | 0,9947 |

a) b)


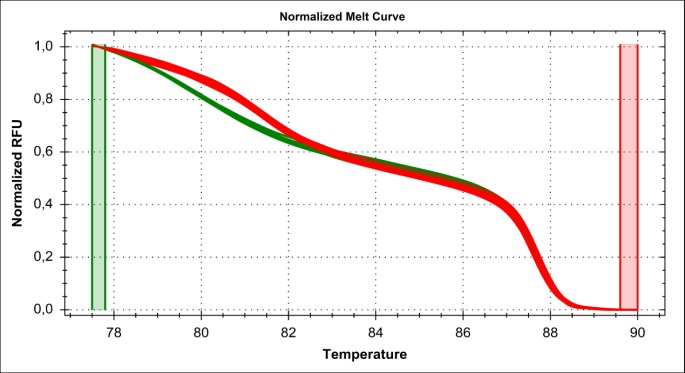

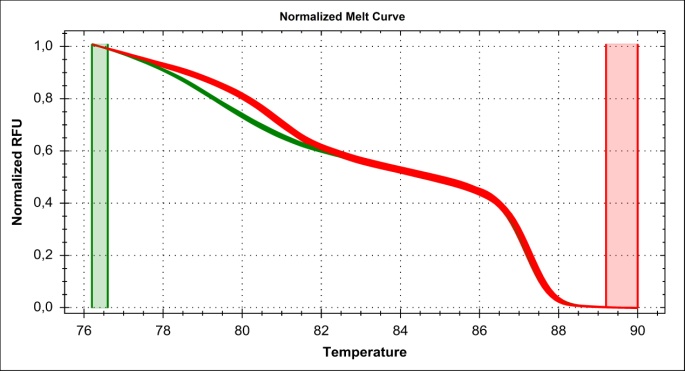


c)


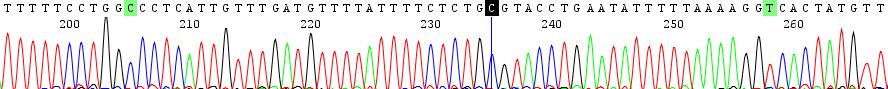


d)


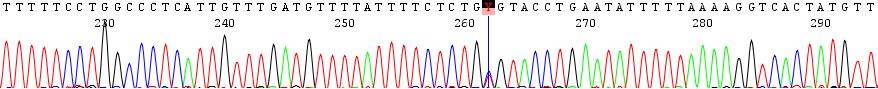


**Figure S19 Summary of *ABCC1* exon 12 scanning by HRM: a), b) melting plots for the second scanned area of the exon (NC_00016.10: 16056185 – 16056390, including primers). Examples of sequencing traces for the samples: c) from the red clusters (no variants), d) from the green clusters (heterozygous variant c.1677+56C>T) (polymorphic base in black boxes).**

**Table S19 Summary of *ABCC1* exon 12 (the second scanned area, NC_00016.10: 16056185 – 16056390) scanning by HRM. Scan rate is the ratio between positive clustered and verified samples to all the scanned samples.**

|  | Plate 1 | Plate 2 | Overall |
| --- | --- | --- | --- |
| Samples scanned | 95 | 95 | 190 |
| Melting clusters | 2 | 2 | 4 |
| Samples in cluster 1 (red) | 82 | 80 | 162 |
| Samples in cluster 2 (green) | 13 | 14 | 27 |
| Samples excluded | 0 | 1 | 1 |
| Samples verified by sequencing from cluster 1 | 2 | 2 | 4 |
| Samples verified by sequencing from cluster 2 | 2 | 2 | 4 |
| Scan rate | - | - | 0,9947 |

a) b)


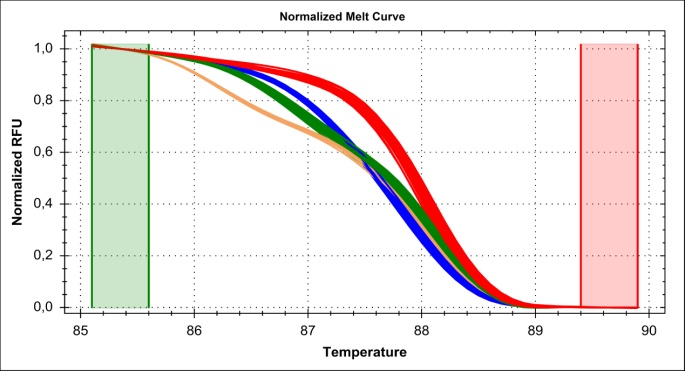

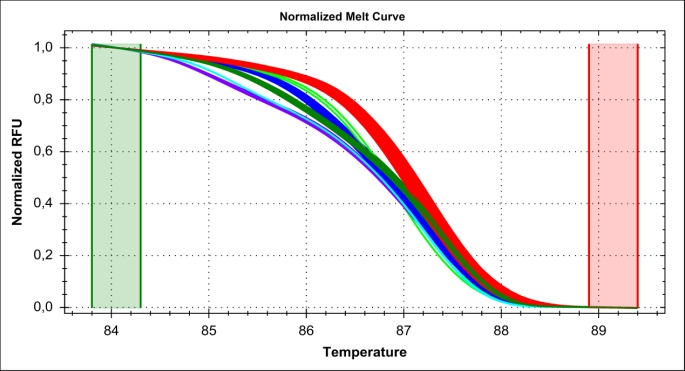


c)


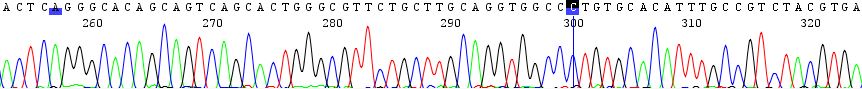


d)


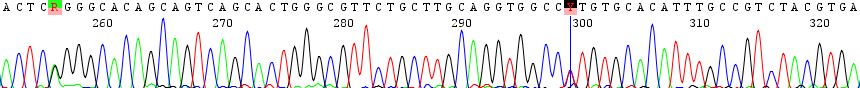


e)


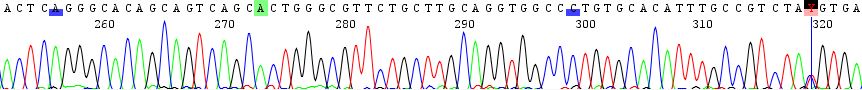


f)


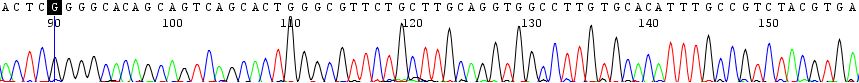


g)

h)

i)

**Figure S20 Summary of *ABCC1* exon 13 scanning by HRM: a), b) melting plots for the first scanned area of the exon (NC_00016.10: 16068075 – 16068258, including primers). Examples of sequencing traces for the samples: c) from the red clusters (homozygous variants c.1678-37G>A and c.1684T>C (in black box)), d) from the green clusters (heterozygous variants c.1678-37G>A and c.1684T>C (in black box)), e) from the blue clusters (homozygous variants c.1678-37G>A and c.1684T>C and heterozygous variant c.1704C>T (in black box)), f) from the orange cluster (no variants), g) from the violet cluster (heterozygous variants c.1678-37G>A and c.1684T>C and c.1704C>T (in black box)), h) from the cyan cluster (heterozygous variants c.1678-37G>A and 1678-34G>A (the novel one, in black box) and c.1684T>C), i) from the lime cluster (homozygous variants c.1678-37G>A and c.1684T>C and 1704C>T (in black box)).**

**Table S20 Summary of *ABCC1* exon 13 (the first scanned area, NC_00016.10: 16068075 – 16068258) scanning by HRM. Scan rate is the ratio between positive clustered and verified samples to all the scanned samples.**

|  | Plate 1 | Plate 2 | Overall |
| --- | --- | --- | --- |
| Samples scanned | 95 | 95 | 190 |
| Melting clusters | 4 | 6 | 10 |
| Samples in cluster 1 (red) | 57 | 55 | 112 |
| Samples in cluster 2 (green) | 22 | 20 | 42 |
| Samples in cluster 3 (blue) | 13 | 16 | 29 |
| Samples in cluster 4 (orange) | 2 | - | 2 |
| Samples in cluster 5 (violet) | - | 2 | 2 |
| Samples in cluster 6 (cyan) | - | 1 | 1 |
| Samples in cluster 7 (lime) | - | 1 | 1 |
| Samples excluded | 1 | 0 | 1 |
| Samples verified by sequencing from cluster 1 | 2 | 2 | 4 |
| Samples verified by sequencing from cluster 2 | 2 | 2 | 4 |
| Samples verified by sequencing from cluster 3 | 2 | 2 | 4 |
| Samples verified by sequencing from cluster 4 | 2 | - | 2 |
| Samples verified by sequencing from cluster 5 | - | 2 | 2 |
| Samples verified by sequencing from cluster 6 | - | 1 | 1 |
| Samples verified by sequencing from cluster 7 | - | 1 | 1 |
| Scan rate | - | - | 0,9947 |

a) b)

c)

**Figure S21 Summary of *ABCC1* exon 13 scanning by HRM: a), b) melting plots for the second scanned area of the exon (NC_00016.10: 16068215 – 16068363, including primers). An example of sequencing trace for the samples: c) from the red clusters, no variants were detected (black boxes indicate the beginning and the end of the HRM product, including primers).**

**Table S21 Summary of *ABCC1* exon 13 (the second scanned area, NC_00016.10: 16068215 – 16068363) scanning by HRM. Scan rate is the ratio between positive clustered and verified samples to all the scanned samples.**

|  | Plate 1 | Plate 2 | Overall |
| --- | --- | --- | --- |
| Samples scanned | 95 | 95 | 190 |
| Melting clusters | 1 | 1 | 2 |
| Samples in cluster 1 (red) | 93 | 92 | 185 |
| Samples excluded | 2 | 3 | 5 |
| Samples verified by sequencing from cluster 1 | 4 | 3 | 7 |
| Scan rate | - | - | 0,9737 |

a) b)

c)

d)

**Figure S22 Summary of *ABCC1* exon 14 scanning by HRM: a), b) melting plots for the whole exon scanned area (NC_00016.10: 16071573 – 16071800, including primers). Examples of sequencing traces for the samples: c) from the red clusters (no variants), d) from the green cluster (heterozygous variant c.1898G>A) (polymorphic base in black boxes).**

**Table S22 Summary of *ABCC1* exon 14 (NC_00016.10: 16071573 – 16071800) scanning by HRM. Scan rate is the ratio between positive clustered and verified samples to all the scanned samples.**

|  | Plate 1 | Plate 2 | Overall |
| --- | --- | --- | --- |
| Samples scanned | 95 | 95 | 190 |
| Melting clusters | 2 | 1 | 3 |
| Samples in cluster 1 (red) | 93 | 94 | 187 |
| Samples in cluster 2 (green) | 1 | - | 1 |
| Samples excluded | 1 | 1 | 2 |
| Samples verified by sequencing from cluster 1 | 2 | 3 | 5 |
| Samples verified by sequencing from cluster 2 | 1 | - | 1 |
| Scan rate | - | - | 0,9895 |

a) b)

c)

**Figure S23 Summary of *ABCC1* exon 15 scanning by HRM: a), b) melting plots for the whole exon scanned area (NC_00016.10: 16076265 – 16076443, including primers). An example of sequencing trace for the samples: c) from the red clusters, no variants were detected (black boxes indicate the beginning and the end of the HRM product, including primers).**

**Table S23 Summary of *ABCC1* exon 15 (NC_00016.10: 16076265 – 16076443) scanning by HRM. Scan rate is the ratio between positive clustered and verified samples to all the scanned samples.**

|  | Plate 1 | Plate 2 | Overall |
| --- | --- | --- | --- |
| Samples scanned | 95 | 95 | 190 |
| Melting clusters | 1 | 1 | 2 |
| Samples in cluster 1 (red) | 95 | 94 | 189 |
| Samples excluded | 0 | 1 | 1 |
| Samples verified by sequencing from cluster 1 | 3 | 3 | 6 |
| Scan rate | - | - | 0,9947 |

a) b)

c)

d)

e)

f)

**Figure S24 Summary of *ABCC1* exon 16 scanning by HRM: a), b) melting plots for the first scanned area of the exon (NC_00016.10: 16079255 – 16079431, including primers). Examples of sequencing traces for the samples: c) from the red clusters (no variants), d) from the green clusters (heterozygous variant c.2012G>T (in black box)), e) from the blue clusters (homozygous variant c.2012G>T (in black box)), f) from the orange cluster (heterozygous variant c.2001C>T (in black box) and heterozygous variant c.2012G>T).**

**Table S24 Summary of *ABCC1* exon 16 (the first scanned area, NC_00016.10: 16079255 – 16079431) scanning by HRM. Scan rate is the ratio between positive clustered and verified samples to all the scanned samples.**

|  | Plate 1 | Plate 2 | Overall |
| --- | --- | --- | --- |
| Samples scanned | 95 | 95 | 190 |
| Melting clusters | 3 | 4 | 7 |
| Samples in cluster 1 (red) | 82 | 79 | 161 |
| Samples in cluster 2 (green) | 11 | 13 | 24 |
| Samples in cluster 3 (blue) | 1 | 1 | 2 |
| Samples in cluster 4 (orange) | - | 1 | 1 |
| Samples excluded | 1 | 1 | 2 |
| Samples verified by sequencing from cluster 1 | 2 | 2 | 4 |
| Samples verified by sequencing from cluster 2 | 2 | 2 | 4 |
| Samples verified by sequencing from cluster 3 | 1 | 1 | 2 |
| Samples verified by sequencing from cluster 4 | - | 1 | 1 |
| Scan rate | - | - | 0,9895 |

a) b)

c)

d)

**Figure S25 Summary of *ABCC1* exon 16 scanning by HRM: a), b) melting plots for the second scanned area of the exon (NC_00016.10: 16079365 – 16079535, including primers). Examples of sequencing traces for the samples: c) from the red clusters, homozygous variant detected in the forward primer region (black boxes indicate the primer region and variant inside), d) from the red clusters, no variants – no additional variants were detected in this area. The region containing the forward primer sequence was scanned in the previous area (see above Figure S24 and Table S24).**

**Table S25 Summary of *ABCC1* exon 16 (the second scanned area, NC_00016.10: 16079365 – 16079535) scanning by HRM. Scan rate is the ratio between positive clustered and verified samples to all the scanned samples.**

|  | Plate 1 | Plate 2 | Overall |
| --- | --- | --- | --- |
| Samples scanned | 95 | 95 | 190 |
| Melting clusters | 1 | 1 | 2 |
| Samples in cluster 1 (red) | 95 | 94 | 189 |
| Samples excluded | 0 | 1 | 1 |
| Samples verified by sequencing from cluster 1 | 3 | 3 | 6 |
| Scan rate | - | - | 0,9947 |

a) b)

c)

d)

**Figure S26 Summary of *ABCC1* exon 17 scanning by HRM: a), b) melting plots for the first scanned area of the exon (NC_00016.10: 16083334 – 16083547, including primers). Examples of sequencing traces for the samples: c) from the red clusters (no variants), d) from the green clusters (heterozygous variant c.2168G>A) (polymorphic base in black boxes).**

**Table S26 Summary of *ABCC1* exon 17 (the first scanned area, NC_00016.10: 16083334 – 16083547) scanning by HRM. Scan rate is the ratio between positive clustered and verified samples to all the scanned samples.**

|  | Plate 1 | Plate 2 | Overall |
| --- | --- | --- | --- |
| Samples scanned | 95 | 95 | 190 |
| Melting clusters | 2 | 2 | 4 |
| Samples in cluster 1 (red) | 91 | 90 | 181 |
| Samples in cluster 2 (green) | 4 | 5 | 9 |
| Samples excluded | 0 | 0 | 0 |
| Samples verified by sequencing from cluster 1 | 3 | 3 | 6 |
| Samples verified by sequencing from cluster 2 | 2 | 2 | 4 |
| Scan rate | - | - | 1 |

a) b)

c)

**Figure S27 Summary of *ABCC1* exon 17 scanning by HRM: a), b) melting plots for the second scanned area of the exon (NC_00016.10: 16083489 – 16083622, including primers). An example of sequencing trace for the samples: c) from the red clusters, no variants were detected (black boxes indicate the beginning and the end of the HRM product, including primers).**

**Table S27 Summary of *ABCC1* exon 17 (the second scanned area, NC_00016.10: 16083489 – 16083622) scanning by HRM. Scan rate is the ratio between positive clustered and verified samples to all the scanned samples.**

|  | Plate 1 | Plate 2 | Overall |
| --- | --- | --- | --- |
| Samples scanned | 95 | 95 | 190 |
| Melting clusters | 1 | 1 | 2 |
| Samples in cluster 1 (red) | 95 | 94 | 189 |
| Samples excluded | 0 | 1 | 1 |
| Samples verified by sequencing from cluster 1 | 3 | 3 | 6 |
| Scan rate | - | - | 0,9947 |

a) b)

c)

**Figure S28 Summary of *ABCC1* exon 18 scanning by HRM: a), b) melting plots for the first scanned area of the exon (NC_00016.10: 16086784 – 16086968, including primers). A example of sequencing trace for the samples: c) from the red clusters, no variants were detected (black boxes indicate the beginning and the end of the HRM product, including primers).**

**Table S28 Summary of *ABCC1* exon 18 (the first scanned area, NC_00016.10: 16086784 – 16086968) scanning by HRM. Scan rate is the ratio between positive clustered and verified samples to all the scanned samples.**

|  | Plate 1 | Plate 2 | Overall |
| --- | --- | --- | --- |
| Samples scanned | 95 | 95 | 190 |
| Melting clusters | 1 | 1 | 2 |
| Samples in cluster 1 (red) | 94 | 94 | 188 |
| Samples excluded | 1 | 1 | 2 |
| Samples verified by sequencing from cluster 1 | 3 | 3 | 6 |
| Scan rate | - | - | 0,9895 |

a) b)

c)

**Figure S29 Summary of *ABCC1* exon 18 scanning by HRM: a), b) melting plots for the second scanned area of the exon (NC_00016.10: 16086901 – 16087066, including primers). An example of sequencing trace for the samples: c) from the red clusters, no variants were detected (black boxes indicate the beginning and the end of the HRM product, including primers).**

**Table S29 Summary of *ABCC1* exon 18 (the second scanned area, NC_00016.10: 16086901 – 16087066) scanning by HRM. Scan rate is the ratio between positive clustered and verified samples to all the scanned samples.**

|  | Plate 1 | Plate 2 | Overall |
| --- | --- | --- | --- |
| Samples scanned | 95 | 95 | 190 |
| Melting clusters | 1 | 1 | 2 |
| Samples in cluster 1 (red) | 94 | 94 | 188 |
| Samples excluded | 1 | 1 | 2 |
| Samples verified by sequencing from cluster 1 | 3 | 3 | 6 |
| Scan rate | - | - | 0,9895 |

a) b)

c)

d)

e)

f)

g)

h)

i)

**Figure S30 Summary of *ABCC1* exon 19 scanning by HRM: a), b) melting plots for the first scanned area of the exon (NC_00016.10: 16090344 – 16090442, including primers). Examples of sequencing traces for the samples: c) from the red clusters (homozygous variant c.2461-30C>G (in black box)), d) from the green clusters (heterozygous variant c.2461-30C>G (in black box)), e) from the blue clusters (homozygous variant c.2461-30C>G and heterozygous variant c.2461-27G>A (in black box)), f) from the orange clusters (deletion variant c.2461-39_2461-38delAT (deletion point highlighted in black) and homozygous variant c.2461-30C>G), g) from the cyan clusters (homozygous variant c.2461-30C>G and homozygous variant c.2461-27G>A (in black box)), h) from the lime cluster (deletion variant c.2461-39_2461-38delAT (deletion point highlighted in black) and homozygous variant c.2461-30C>G and heterozygous variant c.2461-27G>A), i) from the violet cluster (heterozygous variant c.2461-30C>G (in black box) and heterozygous variant c.2461-27G>A).**

**Table S30 Summary of *ABCC1* exon 19 (the first scanned area, NC_00016.10: 16090344 – 16090442) scanning by HRM. Scan rate is the ratio between positive clustered and verified samples to all the scanned samples.**

|  | Plate 1 | Plate 2 | Overall |
| --- | --- | --- | --- |
| Samples scanned | 95 | 95 | 190 |
| Melting clusters | 5 | 7 | 12 |
| Samples in cluster 1 (red) | 62 | 59 | 121 |
| Samples in cluster 2 (green) | 22 | 21 | 43 |
| Samples in cluster 3 (blue) | 5 | 7 | 12 |
| Samples in cluster 4 (orange) | 4 | 4 | 8 |
| Samples in cluster 5 (cyan) | 1 | 1 | 2 |
| Samples in cluster 6 (lime) | - | 1 | 1 |
| Samples in cluster 7 (violet) | - | 1 | 1 |
| Samples excluded | 1 | 1 | 2 |
| Samples verified by sequencing from cluster 1 | 2 | 2 | 4 |
| Samples verified by sequencing from cluster 2 | 2 | 2 | 4 |
| Samples verified by sequencing from cluster 3 | 2 | 2 | 4 |
| Samples verified by sequencing from cluster 4 | 2 | 2 | 4 |
| Samples verified by sequencing from cluster 5 | 1 | 1 | 2 |
| Samples verified by sequencing from cluster 6 | - | 1 | 1 |
| Samples verified by sequencing from cluster 7 | - | 1 | 1 |
| Scan rate | - | - | 0,9895 |

a) b)

c)

d)

**Figure S31 Summary of *ABCC1* exon 19 scanning by HRM: a), b) melting plots for the second scanned area of the exon (NC_00016.10: 16090344 – 16090562, including primers). Examples of sequencing traces for the samples: c) from the red clusters, heterozygous variants detected in the forward primer region (black boxes indicate the primer region and variant inside), d) from the red clusters, homozygous variant detected in the forward primer region – additional scanning area flanking this region was created (see above, Figure S30 and Table S30), no additional variants were detected in this area.**

**Table S31 Summary of *ABCC1* exon 19 (the second scanned area, NC_00016.10: 16090344 – 16090562) scanning by HRM. Scan rate is the ratio between positive clustered and verified samples to all the scanned samples.**

|  | Plate 1 | Plate 2 | Overall |
| --- | --- | --- | --- |
| Samples scanned | 95 | 95 | 190 |
| Melting clusters | 1 | 1 | 2 |
| Samples in cluster 1 (red) | 94 | 92 | 186 |
| Samples excluded | 1 | 3 | 4 |
| Samples verified by sequencing from cluster 1 | 3 | 3 | 6 |
| Scan rate | - | - | 0,9789 |

a) b)

c)

**Figure S32 Summary of *ABCC1* exon 19 scanning by HRM: a), b) melting plots for the third scanned area of the exon (NC_00016.10: 16090483 – 16090664, including primers). An example of sequencing trace for the samples: c) from the red clusters, no variants were detected (black boxes indicate the beginning and the end of the HRM product, including primers).**

**Table S32 Summary of *ABCC1* exon 19 (the third scanned area, NC_00016.10: 16090483 – 16090664) scanning by HRM. Scan rate is the ratio between positive clustered and verified samples to all the scanned samples.**

|  | Plate 1 | Plate 2 | Overall |
| --- | --- | --- | --- |
| Samples scanned | 95 | 95 | 190 |
| Melting clusters | 1 | 1 | 2 |
| Samples in cluster 1 (red) | 95 | 94 | 189 |
| Samples excluded | 0 | 1 | 1 |
| Samples verified by sequencing from cluster 1 | 3 | 3 | 6 |
| Scan rate | - | - | 0,9947 |

a) b)

c)

**Figure S33 Summary of *ABCC1* exon 20 scanning by HRM: a), b) melting plots for the whole exon scanned area (NC_00016.10: 16102559 – 16102781, including primers). An example of sequencing trace for the samples: c) from the red clusters, no variants were detected (black boxes indicate the beginning and the end of the HRM product, including primers).**

**Table S33 Summary of *ABCC1* exon 20 (NC_00016.10: 16102559 – 16102781) scanning by HRM. Scan rate is the ratio between positive clustered and verified samples to all the scanned samples.**

|  | Plate 1 | Plate 2 | Overall |
| --- | --- | --- | --- |
| Samples scanned | 95 | 95 | 190 |
| Melting clusters | 1 | 1 | 2 |
| Samples in cluster 1 (red) | 94 | 94 | 188 |
| Samples excluded | 1 | 1 | 2 |
| Samples verified by sequencing from cluster 1 | 3 | 3 | 6 |
| Scan rate | - | - | 0,9895 |

a) b)

c)

**Figure S34 Summary of *ABCC1* exon 21 scanning by HRM: a), b) melting plots for the whole exon scanned area (NC_00016.10: 16106698 – 16106925, including primers). An example of sequencing trace for the samples: c) from the red clusters, homozygous variant 2871+26C>T (highlighted in blue) detected for all the samples (black boxes indicate the beginning and the end of the HRM product, including primers).**

**Table S34 Summary of *ABCC1* exon 21 (NC_00016.10: 16106698 – 16106925) scanning by HRM. Scan rate is the ratio between positive clustered and verified samples to all the scanned samples.**

|  | Plate 1 | Plate 2 | Overall |
| --- | --- | --- | --- |
| Samples scanned | 95 | 95 | 190 |
| Melting clusters | 1 | 1 | 2 |
| Samples in cluster 1 (red) | 94 | 95 | 189 |
| Samples excluded | 1 | 0 | 1 |
| Samples verified by sequencing from cluster 1 | 3 | 3 | 6 |
| Scan rate | - | - | 0,9947 |

a) b)

c)

d)

**Figure S35 Summary of *ABCC1* exon 22 scanning by HRM: a), b) melting plots for the first scanned area of the exon (NC_00016.10: 16111331 – 16111514, including primers). Examples of sequencing traces for the samples: c) from the red clusters (no variants), d) from the blue cluster (heterozygous variant c.2876A>G) (polymorphic base in black boxes).**

**Table S35 Summary of *ABCC1* exon 22 (the first scanned area, NC_00016.10: 16111331 – 16111514) scanning by HRM. Scan rate is the ratio between positive clustered and verified samples to all the scanned samples.**

|  | Plate 1 | Plate 2 | Overall |
| --- | --- | --- | --- |
| Samples scanned | 95 | 95 | 190 |
| Melting clusters | 2 | 1 | 3 |
| Samples in cluster 1 (red) | 93 | 94 | 187 |
| Samples in cluster 2 (blue) | 1 | - | 1 |
| Samples excluded | 1 | 1 | 2 |
| Samples verified by sequencing from cluster 1 | 3 | 3 | 6 |
| Samples verified by sequencing from cluster 2 | 1 | - | 1 |
| Scan rate | - | - | 0,9895 |

a) b)

c)

d)

e)

f)

**Figure S36 Summary of *ABCC1* exon 22 scanning by HRM: a), b) melting plots for the second scanned area of the exon (NC_00016.10: 16111473 – 16111676, including primers). Examples of sequencing traces for the samples: c) from the red clusters (heterozygous variant c.3079+62T>C (in black box)), d) from the blue clusters (no variants), e) from the green clusters (homozygous variant c.3079+62T>C (in black box)), f) from the orange cluster (heterozygous variant c.3079+24C>T (in black box)), homozygous variant 3079+10G>A (highlighted in blue) detected for all the samples.**

**Table S36 Summary of *ABCC1* exon 22 (the second scanned area, NC_00016.10: 16111473 – 16111676) scanning by HRM. Scan rate is the ratio between positive clustered and verified samples to all the scanned samples.**

|  | Plate 1 | Plate 2 | Overall |
| --- | --- | --- | --- |
| Samples scanned | 95 | 95 | 190 |
| Melting clusters | 3 | 4 | 7 |
| Samples in cluster 1 (red) | 58 | 38 | 96 |
| Samples in cluster 2 (blue) | 24 | 42 | 66 |
| Samples in cluster 3 (green) | 12 | 13 | 25 |
| Samples in cluster 4 (orange) | - | 1 | 1 |
| Samples excluded | 1 | 1 | 2 |
| Samples verified by sequencing from cluster 1 | 2 | 2 | 4 |
| Samples verified by sequencing from cluster 2 | 2 | 2 | 4 |
| Samples verified by sequencing from cluster 3 | 2 | 2 | 4 |
| Samples verified by sequencing from cluster 4 | - | 1 | 1 |
| Scan rate | - | - | 0,9895 |

a) b)

c)

d)

e)

**Figure S37 Summary of *ABCC1* exon 23 scanning by HRM: a), b) melting plots for the first scanned area of the exon (NC_00016.10: 16114725 – 16114938, including primers). Examples of sequencing traces for the samples: c) from the red clusters (no variants), d) from the green cluster (heterozygous variant c.3171G>A (in black box)), e) from the blue cluster (heterozygous variant c.3196C>T (in black box)).**

**Table S37 Summary of *ABCC1* exon 23 (the first scanned area, NC_00016.10: 16114725 – 16114938) scanning by HRM. Scan rate is the ratio between positive clustered and verified samples to all the scanned samples.**

|  | Plate 1 | Plate 2 | Overall |
| --- | --- | --- | --- |
| Samples scanned | 95 | 95 | 190 |
| Melting clusters | 1 | 3 | 4 |
| Samples in cluster 1 (red) | 95 | 91 | 186 |
| Samples in cluster 2 (green) | - | 2 | 2 |
| Samples in cluster 3 (blue) | - | 1 | 1 |
| Samples excluded | 0 | 1 | 1 |
| Samples verified by sequencing from cluster 1 | 3 | 3 | 6 |
| Samples verified by sequencing from cluster 2 | - | 2 | 2 |
| Samples verified by sequencing from cluster 3 | - | 1 | 1 |
| Scan rate | - | - | 0,9947 |

a) b)

c)

**Figure S38 Summary of *ABCC1* exon 23 scanning by HRM: a), b) melting plots for the second scanned area of the exon (NC_00016.10: 16114867 – 16115110, including primers). An example of sequencing trace for the samples: c) from the red clusters, no variants were detected (black boxes indicate the beginning and the end of the HRM product, including primers).**

**Table S38 Summary of *ABCC1* exon 23 (the second scanned area, NC_00016.10: 16114867 – 16115110) scanning by HRM. Scan rate is the ratio between positive clustered and verified samples to all the scanned samples.**

|  | Plate 1 | Plate 2 | Overall |
| --- | --- | --- | --- |
| Samples scanned | 95 | 95 | 190 |
| Melting clusters | 1 | 1 | 2 |
| Samples in cluster 1 (red) | 93 | 93 | 186 |
| Samples excluded | 2 | 2 | 4 |
| Samples verified by sequencing from cluster 1 | 3 | 3 | 6 |
| Scan rate | - | - | 0,9789 |

a) b)

c)

**Figure S39 Summary of *ABCC1* exon 24 scanning by HRM: a), b) melting plots for the first scanned area of the exon (NC_00016.10: 16121932 – 16122143, including primers). An example of sequencing trace for the samples: c) from the red clusters, no variants were detected (black boxes indicate the beginning and the end of the HRM product, including primers).**

**Table S39 Summary of *ABCC1* exon 24 (the first scanned area, NC_00016.10: 16121932 – 16122143) scanning by HRM. Scan rate is the ratio between positive clustered and verified samples to all the scanned samples.**

|  | Plate 1 | Plate 2 | Overall |
| --- | --- | --- | --- |
| Samples scanned | 95 | 95 | 190 |
| Melting clusters | 1 | 1 | 2 |
| Samples in cluster 1 (red) | 95 | 94 | 189 |
| Samples excluded | 0 | 1 | 1 |
| Samples verified by sequencing from cluster 1 | 3 | 3 | 6 |
| Scan rate | - | - | 0,9947 |

a) b)

c)

**Figure S40 Summary of *ABCC1* exon 24 scanning by HRM: a), b) melting plots for the second scanned area of the exon (NC_00016.10: 16122042 – 16122249, including primers). An example of sequencing trace for the samples: c) from the red clusters, no variants were detected (black boxes indicate the beginning and the end of the HRM product, including primers).**

**Table S40 Summary of *ABCC1* exon 24 (the second scanned area, NC_00016.10: 16122042 – 16122249) scanning by HRM. Scan rate is the ratio between positive clustered and verified samples to all the scanned samples.**

|  | Plate 1 | Plate 2 | Overall |
| --- | --- | --- | --- |
| Samples scanned | 95 | 95 | 190 |
| Melting clusters | 1 | 1 | 2 |
| Samples in cluster 1 (red) | 94 | 94 | 188 |
| Samples excluded | 1 | 1 | 2 |
| Samples verified by sequencing from cluster 1 | 3 | 3 | 6 |
| Scan rate | - | - | 0,9895 |

a) b)

c)

d)

**Figure S41 Summary of *ABCC1* exon 25 scanning by HRM: a), b) melting plots for the whole exon scanned area (NC_00016.10: 16124743 – 16124967, including primers). Examples of sequencing traces for the samples: c) from the red clusters (no variants), d) from the green cluster (heterozygous variant c.3591-5C>T) (polymorphic base in black boxes).**

**Table S41 Summary of *ABCC1* exon 25 (NC_00016.10: 16124743 – 16124967) scanning by HRM. Scan rate is the ratio between positive clustered and verified samples to all the scanned samples.**

|  | Plate 1 | Plate 2 | Overall |
| --- | --- | --- | --- |
| Samples scanned | 95 | 95 | 190 |
| Melting clusters | 1 | 2 | 3 |
| Samples in cluster 1 (red) | 94 | 93 | 187 |
| Samples in cluster 2 (green) | - | 1 | 1 |
| Samples excluded | 1 | 1 | 2 |
| Samples verified by sequencing from cluster 1 | 3 | 2 | 5 |
| Samples verified by sequencing from cluster 2 | - | 1 | 1 |
| Scan rate | - | - | 0,9895 |

a) b)

c)

**Figure S42 Summary of *ABCC1* exon 26 scanning by HRM: a), b) melting plots for the whole exon scanned area (NC_00016.10: 16125766 – 16125949, including primers). An example of sequencing trace for the samples: c) from the red clusters, no variants were detected (black boxes indicate the beginning and the end of the HRM product, including primers).**

**Table S42 Summary of *ABCC1* exon 26 (NC_00016.10: 16125766 – 16125949) scanning by HRM. Scan rate is the ratio between positive clustered and verified samples to all the scanned samples.**

|  | Plate 1 | Plate 2 | Overall |
| --- | --- | --- | --- |
| Samples scanned | 95 | 95 | 190 |
| Melting clusters | 1 | 1 | 2 |
| Samples in cluster 1 (red) | 95 | 93 | 188 |
| Samples excluded | 0 | 2 | 2 |
| Samples verified by sequencing from cluster 1 | 3 | 3 | 6 |
| Scan rate | - | - | 0,9895 |

a) b)

c)

d)

e)

**Figure S43 Summary of *ABCC1* exon 27 scanning by HRM: a), b) melting plots for the whole exon scanned area (NC_00016.10: 16131724 – 16131971, including primers). Examples of sequencing traces for the samples: c) from the red clusters (no variants), d) from the green cluster, one sample (heterozygous variant c.3886C>T), e) from the green cluster, one sample (heterozygous variant c.3901C>T) (polymorphic bases in black boxes).**

**Table S43 Summary of *ABCC1* exon 27 (NC_00016.10: 16131724 – 16131971) scanning by HRM. Scan rate is the ratio between positive clustered and verified samples to all the scanned samples.**

|  | Plate 1 | Plate 2 | Overall |
| --- | --- | --- | --- |
| Samples scanned | 95 | 95 | 190 |
| Melting clusters | 2 | 1 | 3 |
| Samples in cluster 1 (red) | 92 | 94 | 186 |
| Samples in cluster 2 (green) | 2 | - | 2 |
| Samples excluded | 1 | 1 | 2 |
| Samples verified by sequencing from cluster 1 | 2 | 3 | 5 |
| Samples verified by sequencing from cluster 2 | 2 | - | 2 |
| Scan rate | - | - | 0,9895 |

a) b)

c)

d)

e)

**Figure S44 Summary of *ABCC1* exon 28 scanning by HRM: a), b) melting plots for the first scanned area of the exon (NC_00016.10: 16134325 – 16134437, including primers). Examples of sequencing traces for the samples: c) from the red clusters (no variants), d) from the green cluster (heterozygous variant c.4002G>A), e) from the blue cluster (homozygous variant c.4002G>A) (polymorphic base in black boxes).**

**Table S44 Summary of *ABCC1* exon 28 (the first scanned area, NC_00016.10: 16134325 – 16134437) scanning by HRM. Scan rate is the ratio between positive clustered and verified samples to all the scanned samples.**

|  | Plate 1 | Plate 2 | Overall |
| --- | --- | --- | --- |
| Samples scanned | 95 | 95 | 190 |
| Melting clusters | 2 | 1 | 3 |
| Samples in cluster 1 (red) | 53 | 49 | 102 |
| Samples in cluster 2 (green) | 32 | 36 | 68 |
| Samples in cluster 3 (blue) | 9 | 9 | 18 |
| Samples excluded | 1 | 1 | 2 |
| Samples verified by sequencing from cluster 1 | 2 | 2 | 4 |
| Samples verified by sequencing from cluster 2 | 2 | 2 | 4 |
| Samples verified by sequencing from cluster 3 | 2 | 2 | 4 |
| Scan rate | - | - | 0,9895 |

a) b)

c)

d)

**Figure S45 Summary of *ABCC1* exon 28 scanning by HRM: a), b) melting plots for the second scanned area of the exon (NC_00016.10: 16134395 – 16134568, including primers). Examples of sequencing traces for the samples: c) from the red clusters (no variants), d) from the green cluster (heterozygous variant c.4093G>A) (polymorphic bases in black boxes).**

**Table S45 Summary of *ABCC1* exon 28 (the second scanned area, NC_00016.10: 16134395 – 16134568) scanning by HRM. Scan rate is the ratio between positive clustered and verified samples to all the scanned samples.**

|  | Plate 1 | Plate 2 | Overall |
| --- | --- | --- | --- |
| Samples scanned | 95 | 95 | 190 |
| Melting clusters | 2 | 1 | 3 |
| Samples in cluster 1 (red) | 93 | 94 | 187 |
| Samples in cluster 2 (green) | 1 | - | 1 |
| Samples excluded | 1 | 1 | 2 |
| Samples verified by sequencing from cluster 1 | 2 | 3 | 5 |
| Samples verified by sequencing from cluster 2 | 1 | - | 1 |
| Scan rate | - | - | 0,9895 |

a) b)

c)

d)

e)

**Figure S46 Summary of *ABCC1* exon 29 scanning by HRM: a), b) melting plots for the first scanned area of the exon (NC_00016.10: 16136403 – 16136629, including primers). Examples of sequencing traces for the samples: c) from the red clusters (heterozygous variant c.4126-45G>A), d) from the green cluster (no variants), e) from the blue cluster (homozygous variant c.4126-45G>A) (polymorphic bases in black boxes).**

**Table S46 Summary of *ABCC1* exon 29 (the first scanned area, NC_00016.10: 16136403 – 16136629) scanning by HRM. Scan rate is the ratio between positive clustered and verified samples to all the scanned samples.**

|  | Plate 1 | Plate 2 | Overall |
| --- | --- | --- | --- |
| Samples scanned | 95 | 95 | 190 |
| Melting clusters | 3 | 3 | 6 |
| Samples in cluster 1 (red) | 40 | 45 | 85 |
| Samples in cluster 2 (green) | 35 | 27 | 62 |
| Samples in cluster 3 (blue) | 20 | 22 | 42 |
| Samples excluded | 0 | 1 | 1 |
| Samples verified by sequencing from cluster 1 | 2 | 2 | 4 |
| Samples verified by sequencing from cluster 2 | 2 | 2 | 4 |
| Samples verified by sequencing from cluster 3 | 2 | 2 | 4 |
| Scan rate | - | - | 0,9947 |

a) b)

c)

**Figure S47 Summary of *ABCC1* exon 29 scanning by HRM: a), b) melting plots for the second scanned area of the exon (NC_00016.10: 16136569 – 16136711, including primers). An example of sequencing trace for the samples: c) from the red clusters, no variants were detected (black boxes indicate the beginning and the end of the HRM product, including primers).**

**Table S47 Summary of *ABCC1* exon 29 (the second scanned part, NC_00016.10: 16136569 – 16136711) scanning by HRM. Scan rate is the ratio between positive clustered and verified samples to all the scanned samples.**

|  | Plate 1 | Plate 2 | Overall |
| --- | --- | --- | --- |
| Samples scanned | 95 | 95 | 190 |
| Melting clusters | 1 | 1 | 2 |
| Samples in cluster 1 (red) | 94 | 94 | 188 |
| Samples excluded | 1 | 1 | 2 |
| Samples verified by sequencing from cluster 1 | 3 | 3 | 6 |
| Scan rate | - | - | 0,9895 |

a) b)

c)

**Figure S48 Summary of *ABCC1* exon 30 scanning by HRM: a), b) melting plots for the first scanned area of the exon (NC_00016.10: 16138270 – 16138486, including primers). An example of sequencing trace for the samples: c) from the red clusters, no variants were detected (black boxes indicate the beginning and the end of the HRM product, including primers).**

**Table S48 Summary of *ABCC1* exon 30 (the first scanned part, NC_00016.10: 16138270 – 16138486) scanning by HRM. Scan rate is the ratio between positive clustered and verified samples to all the scanned samples.**

|  | Plate 1 | Plate 2 | Overall |
| --- | --- | --- | --- |
| Samples scanned | 95 | 95 | 190 |
| Melting clusters | 1 | 1 | 2 |
| Samples in cluster 1 (red) | 95 | 94 | 189 |
| Samples excluded | 0 | 1 | 1 |
| Samples verified by sequencing from cluster 1 | 3 | 3 | 6 |
| Scan rate | - | - | 0,9947 |

a) b)

c)

d)

e)

**Figure S49 Summary of *ABCC1* exon 30 scanning by HRM: a), b) melting plots for the second scanned area of the exon (NC_00016.10: 16138400 – 16138603, including primers). Examples of sequencing traces for the samples: c) from the red clusters (no variants), d) from the green clusters (heterozygous variant c.4487+18G>A), e) from the orange clusters (homozygous variant c.4487+18G>A) (polymorphic bases in black boxes).**

**Table S49 Summary of *ABCC1* exon 30 (the second scanned area, NC_00016.10: 16138400 – 16138603) scanning by HRM. Scan rate is the ratio between positive clustered and verified samples to all the scanned samples.**

|  | Plate 1 | Plate 2 | Overall |
| --- | --- | --- | --- |
| Samples scanned | 95 | 95 | 190 |
| Melting clusters | 3 | 3 | 6 |
| Samples in cluster 1 (red) | 68 | 68 | 136 |
| Samples in cluster 2 (green) | 24 | 23 | 47 |
| Samples in cluster 3 (orange) | 1 | 4 | 5 |
| Samples excluded | 2 | 0 | 2 |
| Samples verified by sequencing from cluster 1 | 2 | 2 | 4 |
| Samples verified by sequencing from cluster 2 | 2 | 2 | 4 |
| Samples verified by sequencing from cluster 3 | 1 | 2 | 3 |
| Scan rate | - | - | 0,9895 |

a) b)

c)

d)

e)

**Figure S50 Summary of *ABCC1* exon 31 scanning by HRM: a), b) melting plots for the whole exon scanned area (NC_00016.10: 16141138 – 16141322, including primers). Examples of sequencing traces for the samples: c) from the red clusters (no variants), d) from the green cluster (heterozygous variant c.4551G>A, the novel one), e) from the blue cluster (homozygous variant c.*3C>G) (polymorphic bases in black boxes).**

**Table S50 Summary of *ABCC1* exon 31 (NC_00016.10: 16141138 – 16141322) scanning by HRM. Scan rate is the ratio between positive clustered and verified samples to all the scanned samples.**

|  | Plate 1 | Plate 2 | Overall |
| --- | --- | --- | --- |
| Samples scanned | 95 | 95 | 190 |
| Melting clusters | 2 | 2 | 4 |
| Samples in cluster 1 (red) | 93 | 92 | 185 |
| Samples in cluster 2 (green) | 1 | - | 1 |
| Samples in cluster 3 (blue) | - | 1 | 1 |
| Samples excluded | 1 | 2 | 3 |
| Samples verified by sequencing from cluster 1 | 3 | 3 | 6 |
| Samples verified by sequencing from cluster 2 | 1 | - | 1 |
| Samples verified by sequencing from cluster 3 | - | 1 | 1 |
| Scan rate | - | - | 0,9842 |

**Table S51 Summary of *ABCC1* scanning by HRM method. The average scan rate for the all the *ABCC1* scanned areas and the number of total sequenced samples are on the bottom of the table.**

| Scanned exon | Area of the exon | Clustered samples | Excluded samples | Sequenced samples | Scan rate |
| --- | --- | --- | --- | --- | --- |
| 2 | 1 | 189 | 1 | 6 | 0,9947 |
| 2 | 2 | 189 | 1 | 9 | 0,9947 |
| 3 | whole | 188 | 2 | 6 | 0,9895 |
| 4 | 1 | 186 | 4 | 14 | 0,9789 |
| 4 | 2 | 189 | 1 | 11 | 0,9947 |
| 4 | 3 | 188 | 2 | 6 | 0,9895 |
| 5 | whole | 187 | 3 | 6 | 0,9842 |
| 6 | whole | 188 | 2 | 6 | 0,9895 |
| 7 | 1 | 189 | 1 | 11 | 0,9947 |
| 7 | 2 | 188 | 2 | 25 | 0,9895 |
| 8 | 1 | 188 | 2 | 19 | 0,9895 |
| 8 | 2 | 189 | 1 | 6 | 0,9947 |
| 9 | 1 | 185 | 5 | 12 | 0,9737 |
| 9 | 2 | 189 | 1 | 12 | 0,9947 |
| 10 | 1 | 188 | 2 | 8 | 0,9895 |
| 10 | 2 | 187 | 3 | 5 | 0,9842 |
| 11 | whole | 188 | 2 | 7 | 0,9895 |
| 12 | 1 | 189 | 1 | 6 | 0,9947 |
| 12 | 2 | 189 | 1 | 8 | 0,9947 |
| 13 | 1 | 189 | 1 | 18 | 0,9947 |
| 13 | 2 | 185 | 5 | 7 | 0,9737 |
| 14 | whole | 188 | 2 | 6 | 0,9895 |
| 15 | whole | 189 | 1 | 6 | 0,9947 |
| 16 | 1 | 188 | 2 | 11 | 0,9895 |
| 16 | 2 | 189 | 1 | 6 | 0,9947 |
| 17 | 1 | 190 | 0 | 10 | 1 |
| 17 | 2 | 189 | 1 | 6 | 0,9947 |
| 18 | 1 | 188 | 2 | 6 | 0,9895 |
| 18 | 2 | 188 | 2 | 6 | 0,9895 |
| 19 | 1 | 188 | 2 | 20 | 0,9895 |
| 19 | 2 | 186 | 4 | 6 | 0,9789 |
| 19 | 3 | 189 | 1 | 6 | 0,9947 |
| 20 | whole | 188 | 2 | 6 | 0,9895 |
| 21 | whole | 189 | 1 | 6 | 0,9947 |
| 22 | 1 | 188 | 2 | 7 | 0,9895 |
| 22 | 2 | 188 | 2 | 13 | 0,9895 |
| 23 | 1 | 189 | 1 | 9 | 0,9947 |
| 23 | 2 | 186 | 4 | 6 | 0,9789 |
| 24 | 1 | 189 | 1 | 6 | 0,9947 |
| 24 | 2 | 188 | 2 | 6 | 0,9895 |
| 25 | whole | 188 | 2 | 6 | 0,9895 |
| 26 | whole | 188 | 2 | 6 | 0,9895 |
| 27 | whole | 188 | 2 | 7 | 0,9895 |
| 28 | 1 | 188 | 2 | 12 | 0,9895 |
| 28 | 2 | 188 | 2 | 6 | 0,9895 |
| 29 | 1 | 189 | 1 | 12 | 0,9947 |
| 29 | 2 | 188 | 2 | 6 | 0,9895 |
| 30 | 1 | 189 | 1 | 6 | 0,9947 |
| 30 | 2 | 188 | 2 | 11 | 0,9895 |
| 31 | whole | 187 | 3 | 8 | 0,9842 |
| Overall |  | 9405 | 95 | 437 | 0,9900 |
