# Supplementary material for: Genetic variation of the ABC transporter gene ABCC1 (Multidrug resistance protein 1 – MRP1) in the Polish population
Source: BMC Genet. 2015 Sep 23;16:114. doi: 10.1186/s12863-015-0271-3 (PMC4579605; doi:10.1186/s12863-015-0271-3)
Supplement: Additional file 2: — Supplemental materials for targeted genotyping study (include HRM plots and sequencing traces). (DOCX 5582 kb) [file 12863_2015_271_MOESM2_ESM.docx]

**Table S52 Summary of *ABCC1* c.816G>A (p.Pro272=, rs2230669) and c.825T>C (p.Val275=, rs60782127) genotyping by HRM. Call rate is the ratio between positive clustered to the all genotyped samples.**

|  | Plate 1 | Plate 2 | Plate 3 | Plate 4 | Overall |
| --- | --- | --- | --- | --- | --- |
| Samples genotyped | 95 | 95 | 95 | 95 | 380 |
| Melting clusters | 4 | 4 | 4 | 4 | - |
| Samples in cluster 1  (blue – GG/CC variant) | 9 | 4 | 5 | 5 | 23 |
| Samples in cluster 2  (green – GG/CT variant) | 27 | 40 | 38 | 37 | 142 |
| Samples in cluster 3  (red – GG/TT variant) | 54 | 47 | 48 | 48 | 197 |
| Samples in cluster 4  (violet – GA/CT variant) | 5 | 4 | 4 | 5 | 18 |
| Samples excluded | 0 | 0 | 0 | 0 | 0 |
| Samples veryfied by sequencing from cluster 1 | 1 | 1 | 1 | 1 | 4 |
| Samples veryfied by sequencing from cluster 2 | 1 | 1 | 1 | 1 | 4 |
| Samples veryfied by sequencing from cluster 3 | 1 | 1 | 1 | 1 | 4 |
| Samples veryfied by sequencing from cluster 4 | 1 | 1 | 1 | 1 | 4 |
| Call rate | - | - | - | - | 1 |

a)**
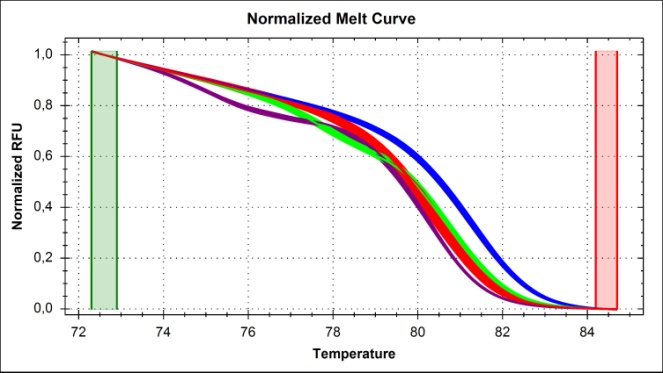

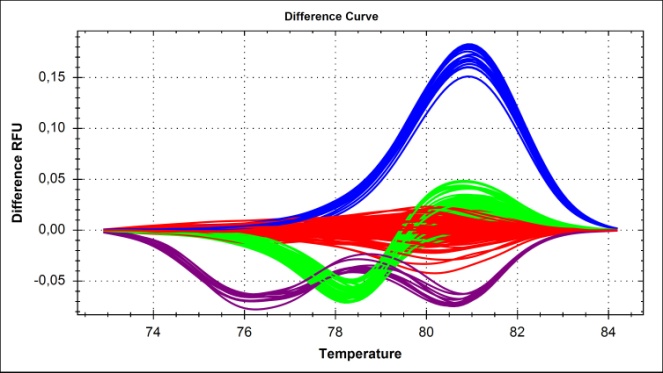
**b)**
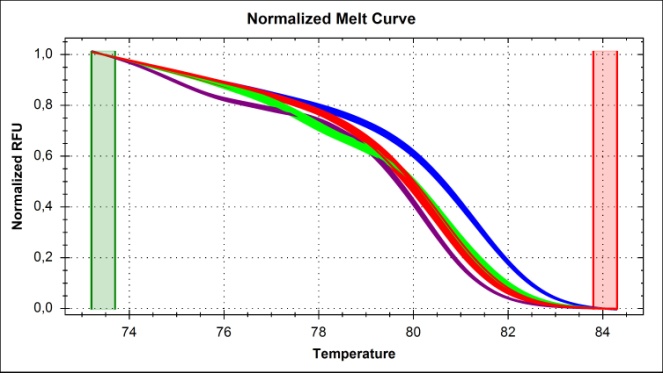

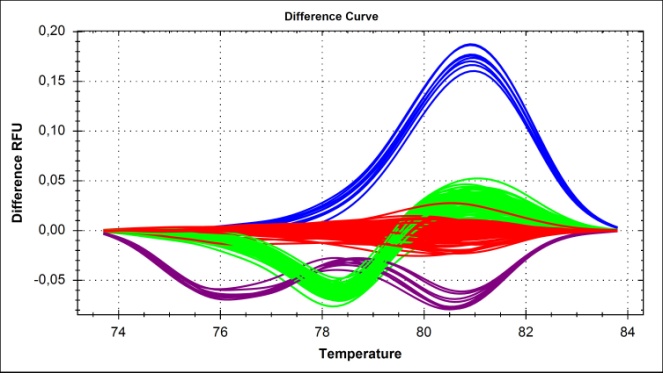
**c)**
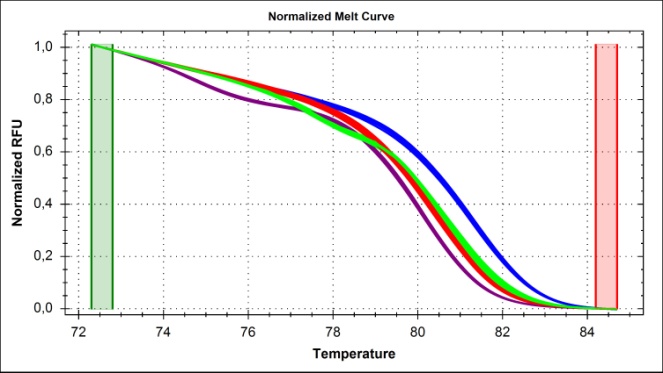

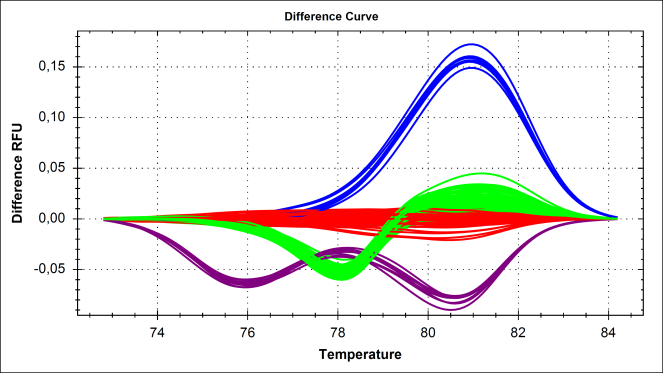
**d)**
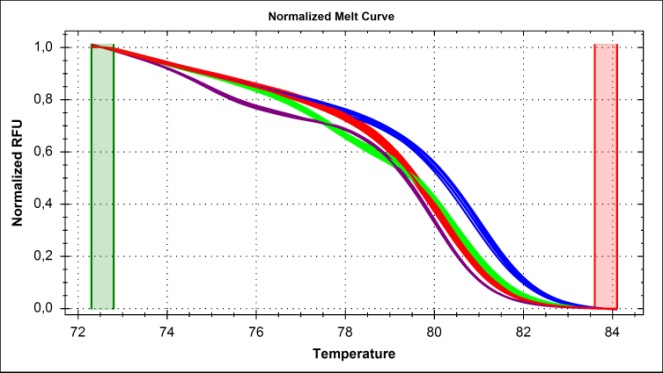

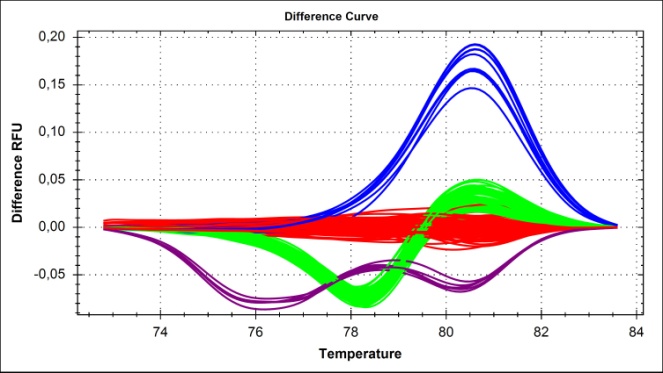
**

e)


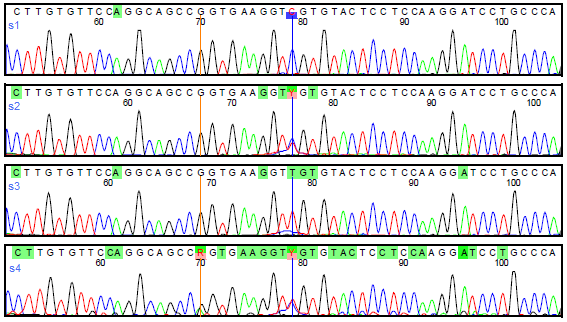


**Figure S51 Summary of *ABCC1* c.816G>A (p.Pro272=, rs2230669) and c.825T>C (p.Val275=, rs60782127) genotyping by HRM: a,b,c,d – High resolution melting profiles of four different DNA plates (analysed PCR product length: 51 bp including primers, 14 bp without primers), blue – cluster 1 (GG/CC), green – cluster 2 (GG/CT), red – cluster 3 (GG/TT), violet – cluster 4 (GA/CT); e – Example of the sequencing traces for the samples from four different clusters: s1 – cluster 1 (GG/CC), s2 – cluster 2 (GG/CT), s3 – cluster 3 (GG/TT); s4 – cluster 4 (GA/CT), red and blue lines indicate c.816G>A and c.825T>C genotype, respectively.**

**Table S53 Summary of *ABCC1* c.1062T>C (p.Asn354=, rs35587) genotyping by HRM. Call rate is the ratio between positive clustered to the all genotyped samples.**

|  | Plate 1 | Plate 2 | Plate 3 | Plate 4 | Overall |
| --- | --- | --- | --- | --- | --- |
| Samples genotyped | 95 | 95 | 95 | 95 | 380 |
| Melting clusters | 3 | 3 | 3 | 3 | - |
| Samples in cluster 1  (blue – CC variant) | 8 | 5 | 12 | 8 | 33 |
| Samples in cluster 2  (pink – CT variant) | 35 | 36 | 37 | 34 | 142 |
| Samples in cluster 3  (green – TT variant) | 51 | 54 | 46 | 53 | 204 |
| Samples excluded | 1 | 0 | 0 | 0 | 1 |
| Samples veryfied by sequencing from cluster 1 | 1 | 1 | 1 | 1 | 4 |
| Samples veryfied by sequencing from cluster 2 | 1 | 1 | 1 | 1 | 4 |
| Samples veryfied by sequencing from cluster 3 | 1 | 1 | 1 | 1 | 4 |
| Call rate | - | - | - | - | 0,9974 |

a)**
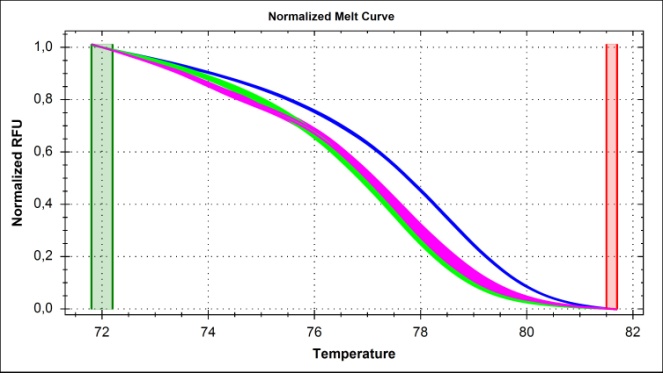

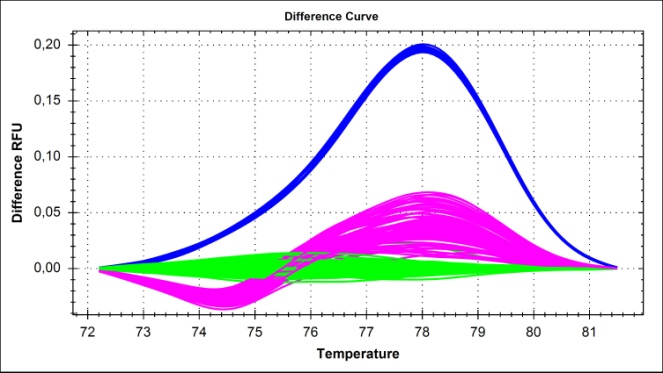
**b)**
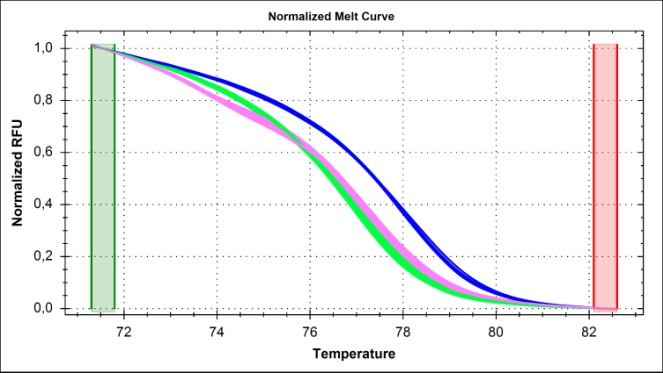

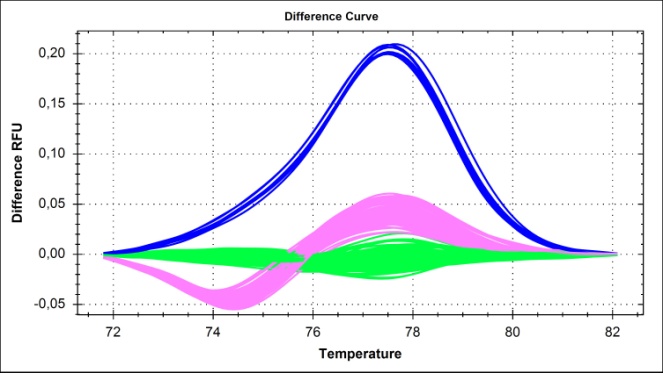
**c)**
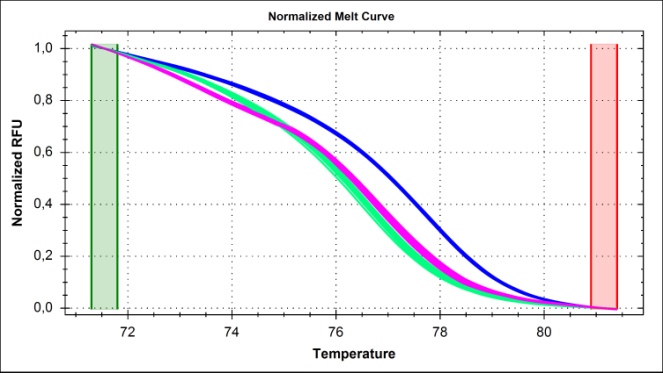

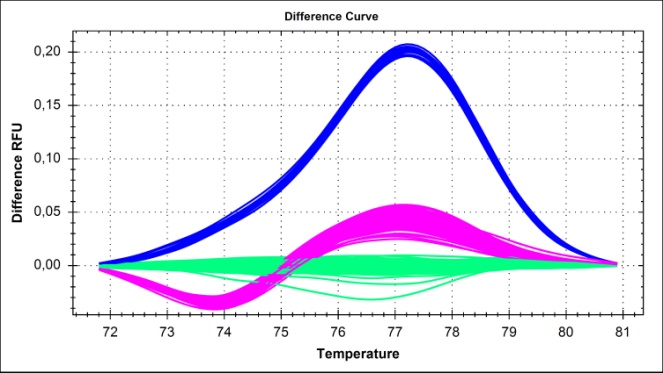
**

d)**
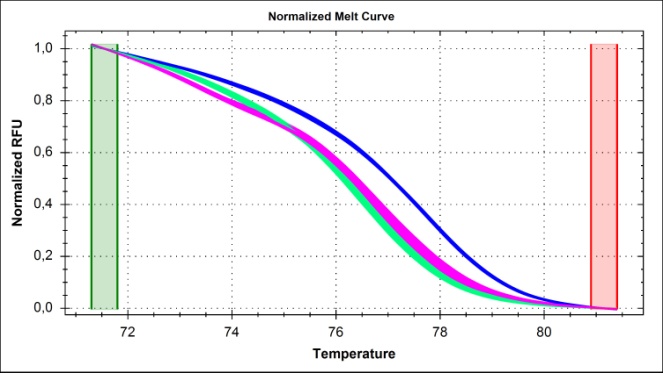

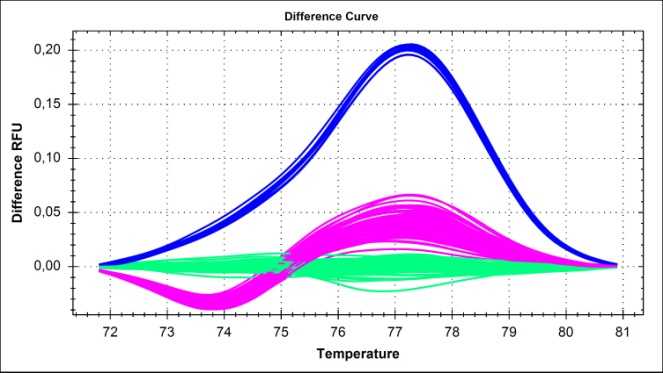
**

e)**
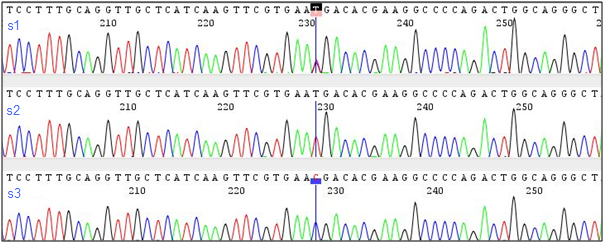
**

**Figure S52 Summary of *ABCC1* c.1062T>C (p.Asn354=, rs35587) genotyping by HRM: a,b,c,d – High resolution melting profiles of four different DNA plates (analysed PCR product length: 41 bp including primers, 2 bp without primers), blue – cluster 1 (CC), pink – cluster 2 (CT), green – cluster 3 (TT); e – Example of the sequencing traces for the samples from three different clusters: s1 – cluster 2 (CT), s2 – cluster 3 (TT), s3 – cluster 1 (CC), blue lines indicate c.1062T>C genotype.**

**Table S54 Summary of *ABCC1* c.1218+8A>G (intron, rs35588) genotyping by HRM. Call rate is the ratio between positive clustered to the all genotyped samples.**

|  | Plate 1 | Plate 2 | Plate 3 | Plate 4 | Overall |
| --- | --- | --- | --- | --- | --- |
| Samples genotyped | 95 | 95 | 95 | 95 | 380 |
| Melting clusters | 3 | 3 | 3 | 3 | - |
| Samples in cluster 1  (blue – GG variant) | 8 | 10 | 6 | 6 | 30 |
| Samples in cluster 2  (pink – AG variant) | 39 | 41 | 40 | 40 | 160 |
| Samples in cluster 3  (green – AA variant) | 48 | 44 | 49 | 49 | 190 |
| Samples excluded | 0 | 0 | 0 | 0 | 0 |
| Samples veryfied by sequencing from cluster 1 | 1 | 1 | 1 | 1 | 4 |
| Samples veryfied by sequencing from cluster 2 | 1 | 1 | 1 | 1 | 4 |
| Samples veryfied by sequencing from cluster 3 | 1 | 1 | 1 | 1 | 4 |
| Call rate | - | - | - | - | 1 |

a)**
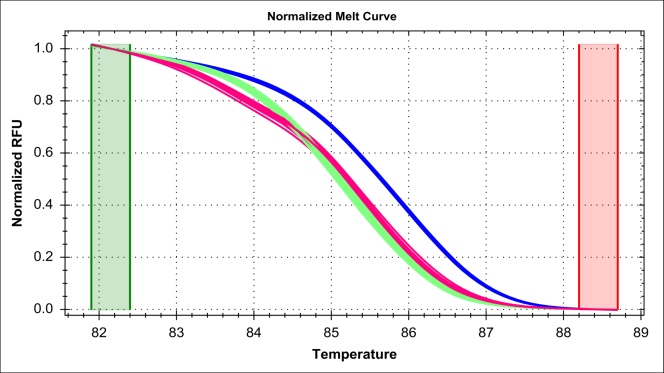

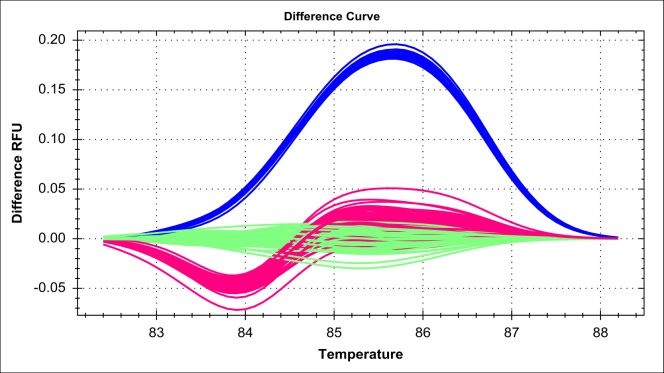
**b)**
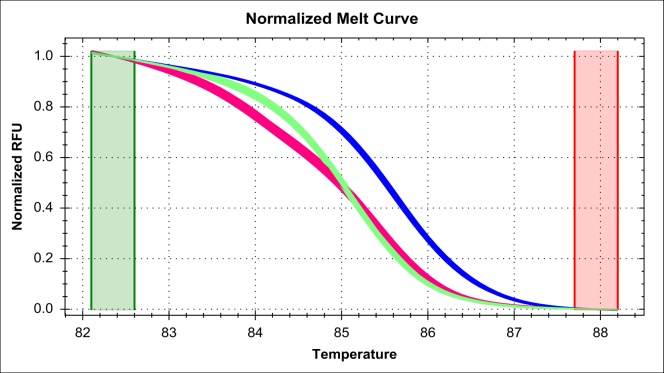

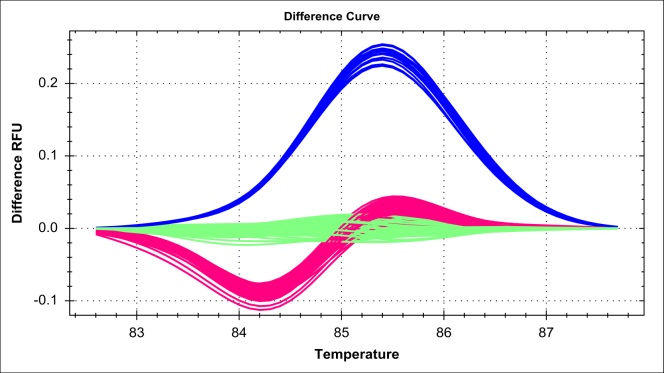
**c)**
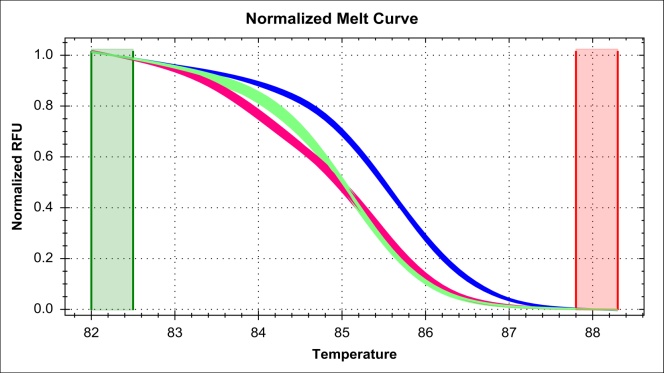

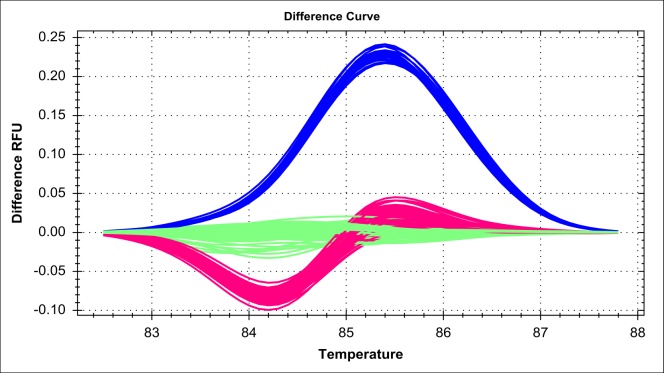
**

d)**
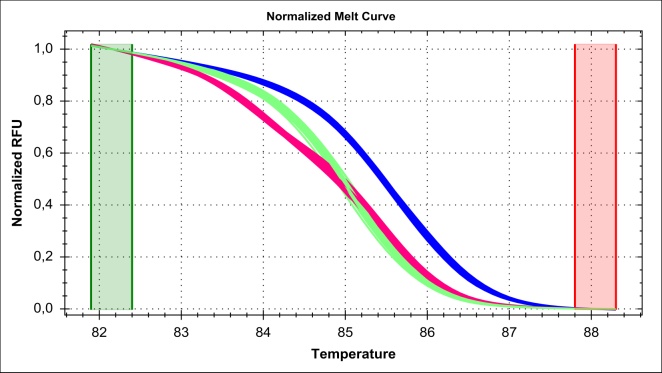

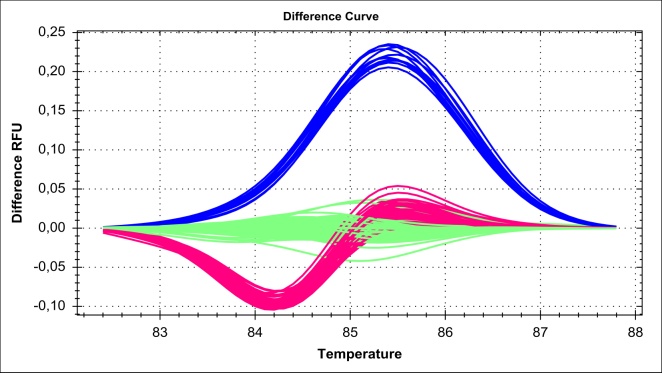
**

e)****

**Figure S53 Summary of *ABCC1* c.1218+8A>G (intron, rs35588) genotyping by HRM: a,b,c,d – High resolution melting profiles of four different DNA plates (analysed PCR product length: 84 bp including primers, 46 bp without primers), blue – cluster 1 (GG), pink – cluster 2 (AG), green – cluster 3 (AA); e – Example of the sequencing traces for the samples from three different clusters: s1 – cluster 1 (GG), s2 – cluster 2 (AG), s3 – cluster 3 (AA), blue lines indicate c.1218+8A>G genotype.**

**Table S55 Summary of *ABCC1* c.1299G>T (p.Arg433Ser, rs60782127) genotyping by HRM. Call rate is the ratio between positive clustered to the all genotyped samples.**

|  | Plate 1 | Plate 2 | Plate 3 | Plate 4 | Overall |
| --- | --- | --- | --- | --- | --- |
| Samples genotyped | 95 | 95 | 95 | 95 | 380 |
| Melting clusters | 2 | 2 | 2 | 2 | - |
| Samples in cluster 1  (red – GG variant) | 94 | 92 | 94 | 93 | 373 |
| Samples in cluster 2  (blue – GT variant) | 1 | 3 | 1 | 1 | 6 |
| Samples excluded | 0 | 0 | 0 | 1 | 0 |
| Samples veryfied by sequencing from cluster 1 | 1 | 1 | 1 | 1 | 4 |
| Samples veryfied by sequencing from cluster 2 | 1 | 3 | 1 | 1 | 6 |
| Call rate | - | - | - | - | 0,9974 |

a)**
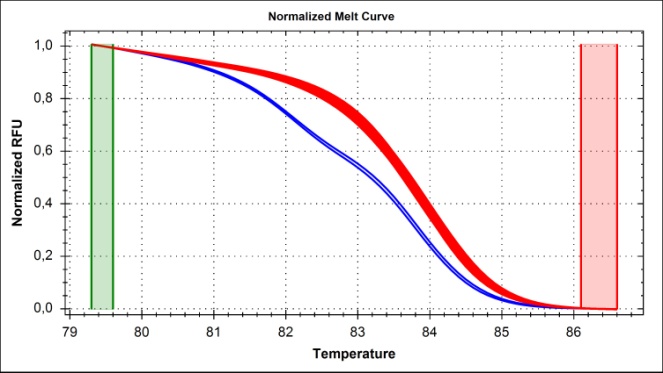

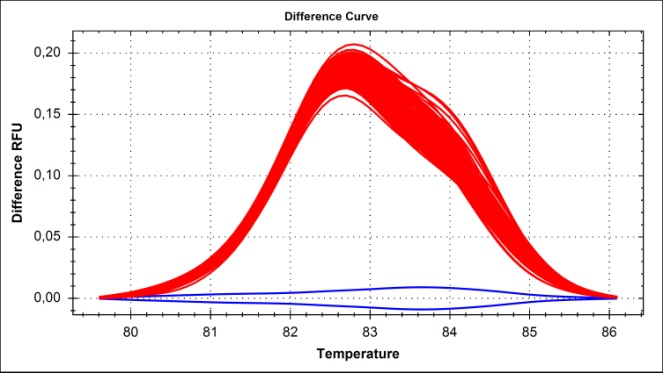
**b)**
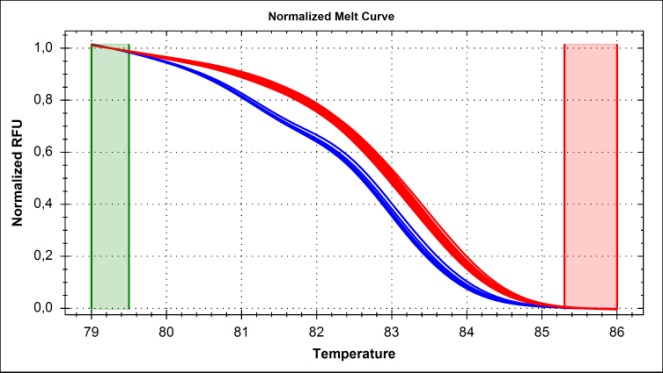

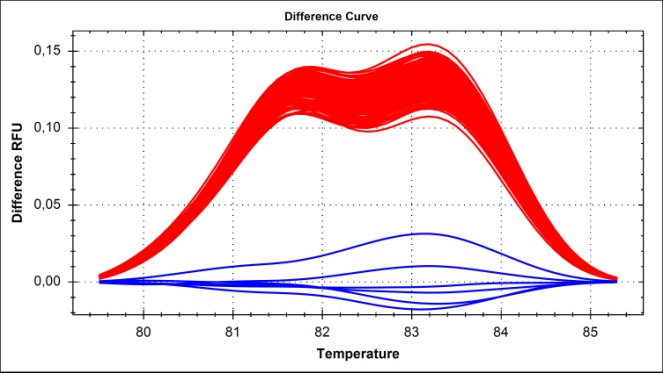
**c)**
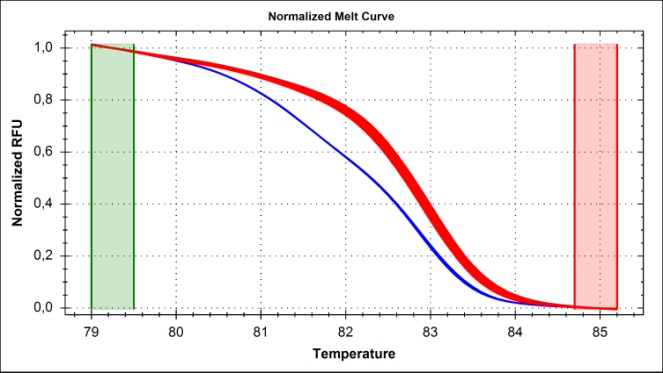

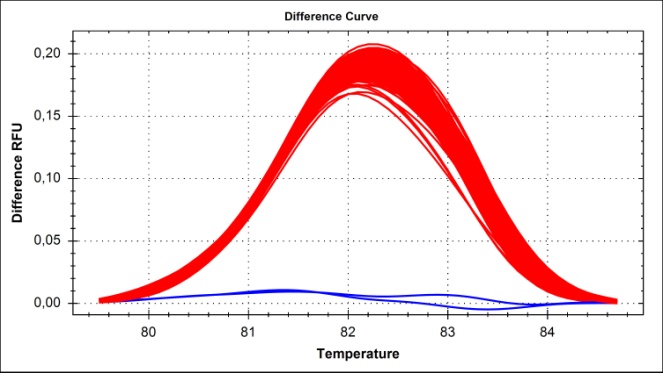
**

d)**
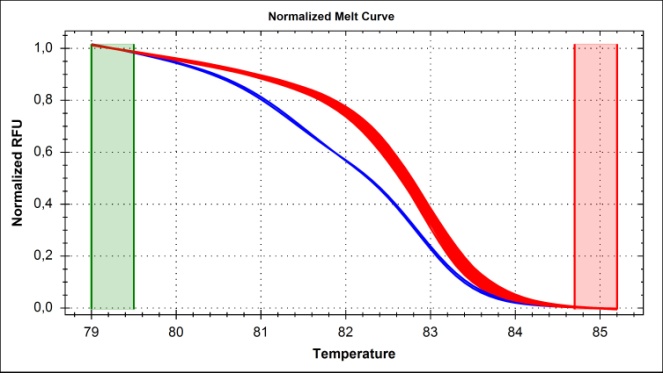

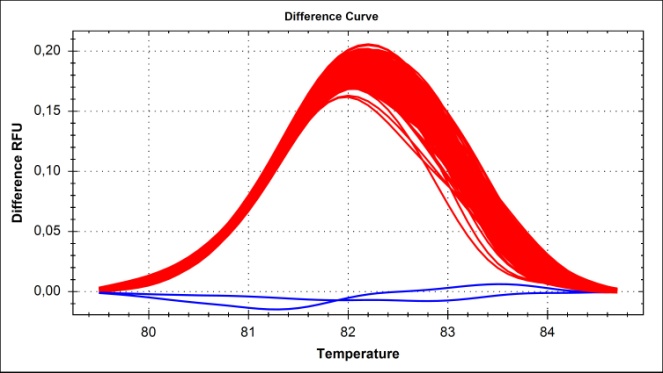
**

e)
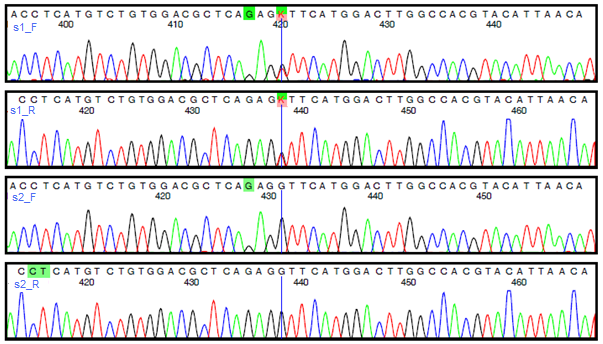


**Figure S54 Summary of *ABCC1* c.1299G>T (p.Arg433Ser, rs60782127) genotyping by HRM: a,b,c,d – High resolution melting profiles of four different DNA plates (analysed PCR product length: 80 bp including primers, 41 bp without primers), red – cluster 1 (GG), blue – cluster 2 (GT) ; e – Example of the sequencing traces for the samples from three different clusters: s1_F&R – cluster 2 (GT), s2_F&R – cluster 1 (GG), blue lines indicate c.1299G>T genotype.**

**Table S56 Summary of *ABCC1* c.1684T>C (p.Leu562=, rs35605) and c.1704C>T (p.Tyr568=, rs8187858) genotyping by HRM. Call rate is the ratio between positive clustered to the all genotyped samples.**

|  | Plate 1 | Plate 2 | Plate 3 | Plate 4 | Overall |
| --- | --- | --- | --- | --- | --- |
| Samples genotyped | 95 | 95 | 95 | 95 | 380 |
| Melting clusters | 5 | 4 | 5 | 5 | - |
| Samples in cluster 1  (pink – CC/CC variant) | 55 | 54 | 51 | 53 | 213 |
| Samples in cluster 2  (violet – CC/CT variant) | 5 | 12 | 16 | 16 | 49 |
| Samples in cluster 3  (blue – CT/CC variant) | 27 | 28 | 19 | 25 | 99 |
| Samples in cluster 4  (green– CT/CT variant) | 2 | 1 | 2 | 1 | 6 |
| Samples in cluster 5  (red– TT/CC variant) | 5 | 0 | 7 | 1 | 13 |
| Samples excluded | 0 | 0 | 0 | 0 | 0 |
| Samples veryfied by sequencing from cluster 1 | 1 | 1 | 1 | 1 | 4 |
| Samples veryfied by sequencing from cluster 2 | 1 | 1 | 1 | 1 | 4 |
| Samples veryfied by sequencing from cluster 3 | 1 | 1 | 1 | 1 | 4 |
| Samples veryfied by sequencing from cluster 4 | 2 | 1 | 2 | 1 | 6 |
| Samples veryfied by sequencing from cluster 5 | 2 | 0 | 2 | 1 | 5 |
| Call rate | - | - | - | - | 1 |

a)**
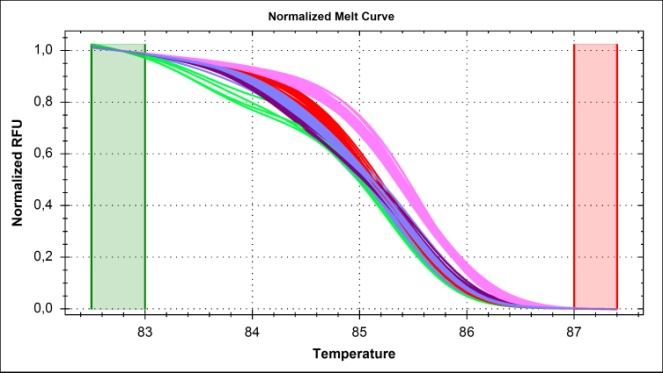

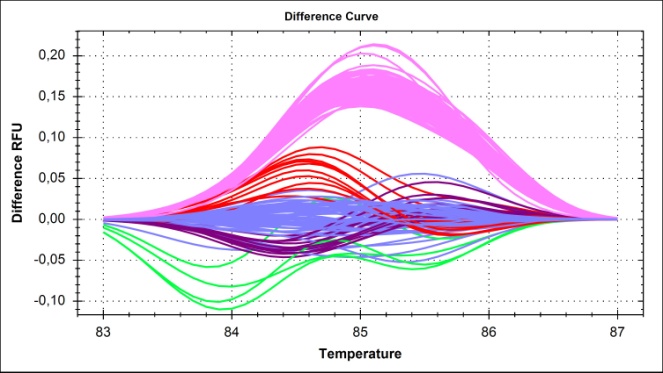
**b)**
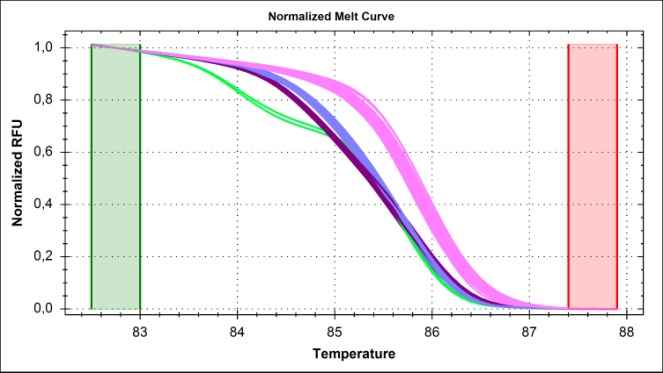

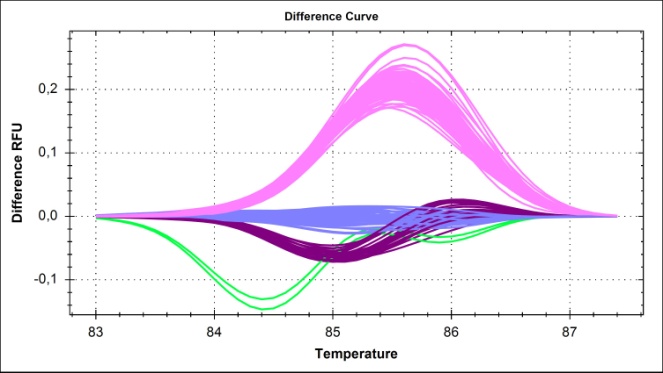
**c)**
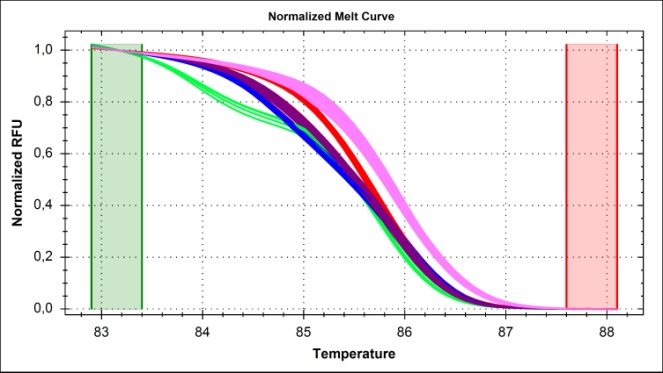

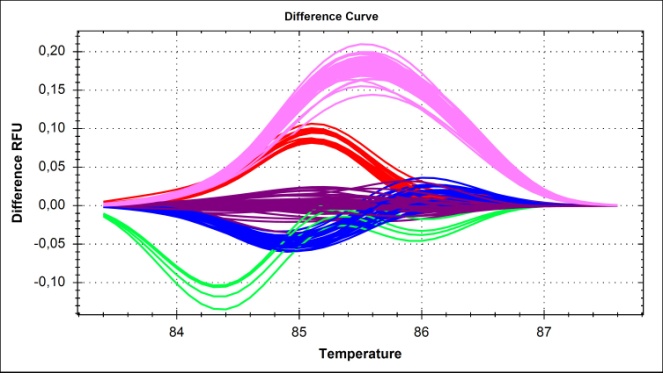
**d)**
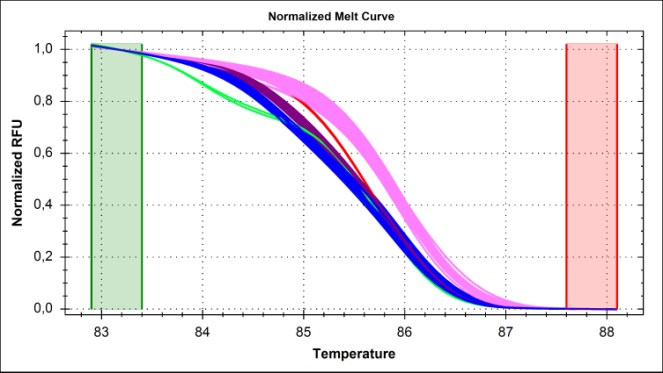

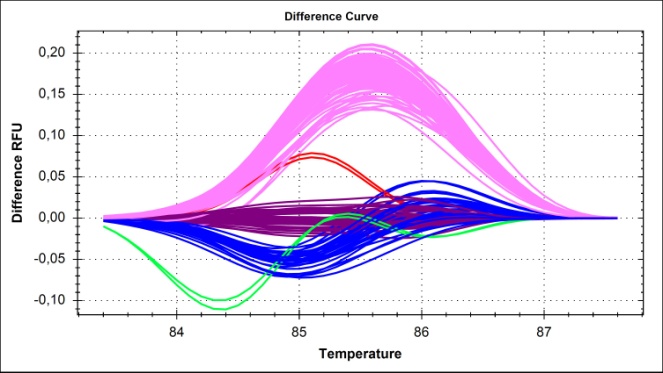
**e) **
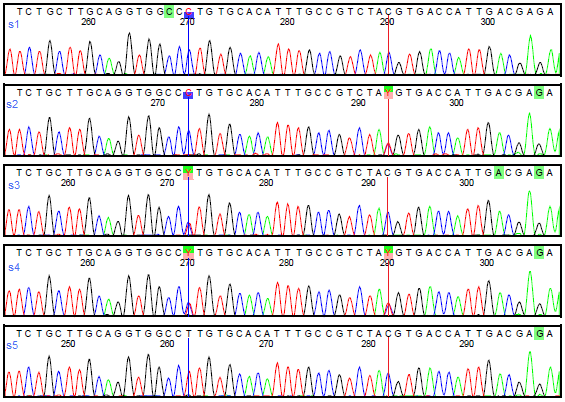
**

**Figure S55 Summary of *ABCC1* c.1684T>C (p.Leu562=, rs35605) and c.1704C>T (p.Tyr568=, rs8187858) genotyping by HRM: a,b,c,d – High resolution melting profiles of four different DNA plates (analysed PCR product length: 125 bp including primers, 89 bp without primers), pink – cluster 1 (CC/CC), violet – cluster 2 (CC/CT), blue – cluster 3 (CT/CC), green – cluster 4 (CT/CT), red – cluster 5 (TT/CC); e – Example of the sequencing traces for the samples from five different clusters: s1 – cluster 1 (CC/CC), s2 – cluster 2 (CC/CT), s3 – cluster 3 (CT/CC), s4 – cluster 4 (CT/CT), s5 – cluster 5 (TT/CC), blue and red lines indicate c.1684T>C and c.1704C>T genotype, respectively.**

**Table S57 Summary of *ABCC1* c.2012G>T (p.Gly671Val, rs45511401) genotyping by HRM. Call rate is the ratio between positive clustered to the all genotyped samples.**

|  | Plate 1 | Plate 2 | Plate 3 | Plate 4 | Overall |
| --- | --- | --- | --- | --- | --- |
| Samples genotyped | 95 | 95 | 95 | 95 | 380 |
| Melting clusters | 3 | 3 | 3 | 3 | - |
| Samples in cluster 1  (red – GG variant) | 88 | 85 | 90 | 83 | 346 |
| Samples in cluster 2  (green – GT variant) | 5 | 8 | 5 | 10 | 28 |
| Samples in cluster 3  (blue – TT variant) | 1 | 2 | 0 | 0 | 3 |
| Samples excluded | 1 | 0 | 0 | 2 | 3 |
| Samples veryfied by sequencing from cluster 1 | 1 | 1 | 1 | 1 | 4 |
| Samples veryfied by sequencing from cluster 2 | 1 | 1 | 1 | 1 | 4 |
| Samples veryfied by sequencing from cluster 3 | 1 | 2 | 0 | 0 | 3 |
| Call rate | - | - | - | - | 0,9921 |

a)**
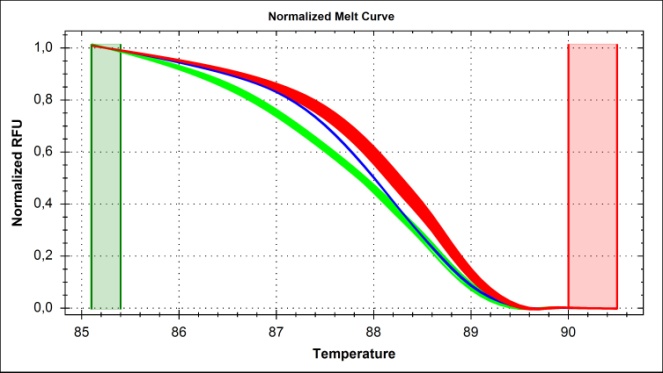

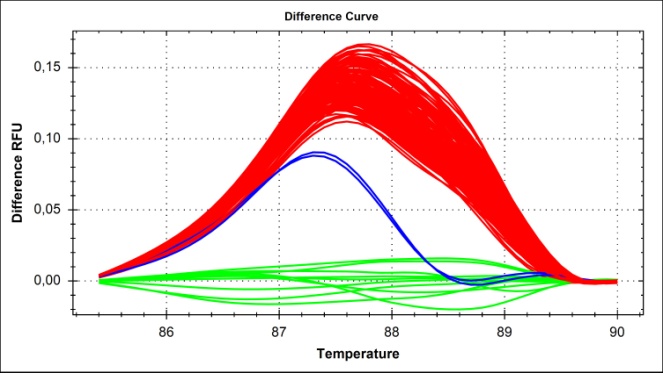
**b)**
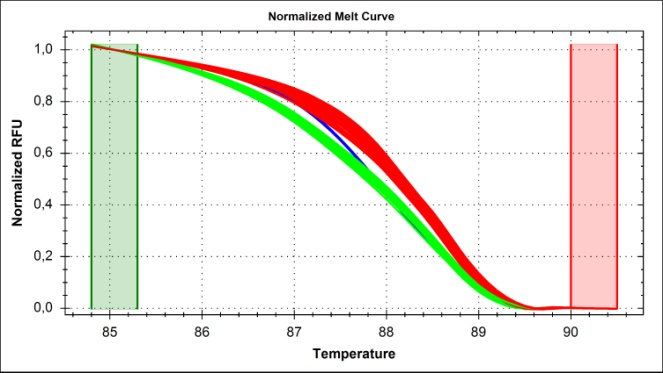

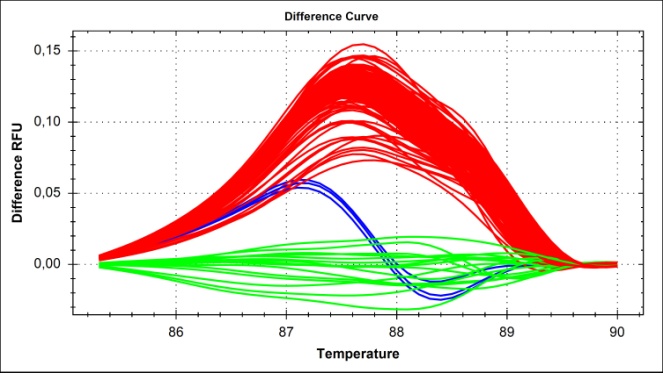
**

c)**
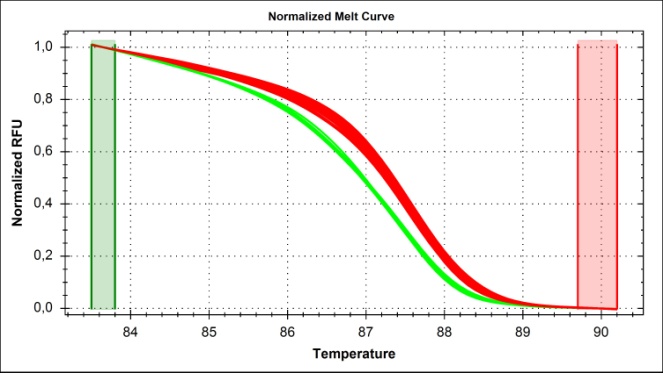

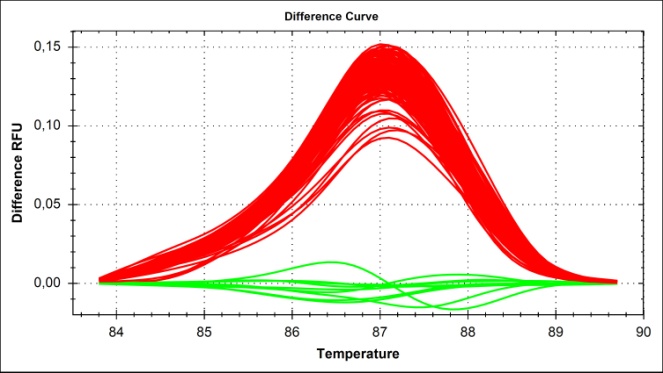
**d)
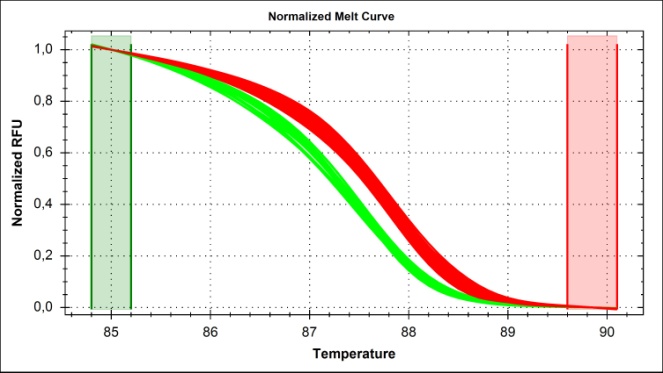

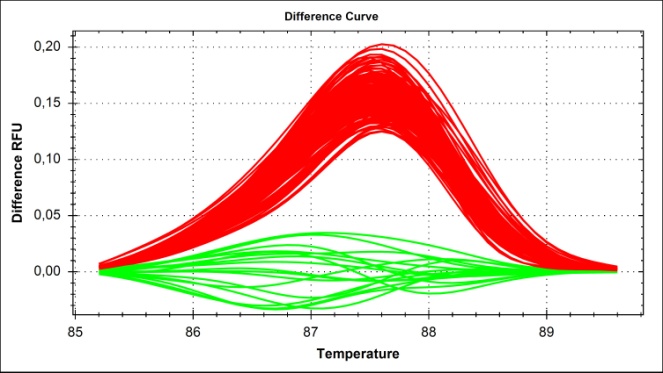


e)


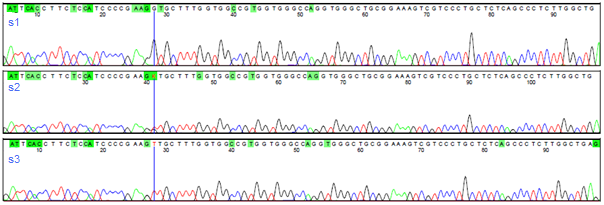


**Figure S56 Summary of *ABCC1* c.2012G>T (p.Gly671Val, rs45511401) genotyping by HRM: a,b,c,d – High resolution melting profiles of four different DNA plates (analysed PCR product length: 104 bp including primers, 66 bp without primers); e – Example of the sequencing traces for the samples from the three different clusters: s1 – cluster 1 (GG), s2 – cluster 2 (GT); s3 – cluster3 (TT), blue lines indicate c.2012G>T genotype.**

**Table S58 Summary of *ABCC1* c.2168G>A (p.Arg723Gln, rs4148356) genotyping by HRM. Call rate is the ratio between positive clustered to the all genotyped samples.**

|  | Plate 1 | Plate 2 | Plate 3 | Plate 4 | Overall |
| --- | --- | --- | --- | --- | --- |
| Samples genotyped | 95 | 95 | 95 | 95 | 380 |
| Melting clusters | 2 | 2 | 2 | 2 | - |
| Samples in cluster 1  (red – GG variant) | 91 | 89 | 90 | 90 | 360 |
| Samples in cluster 2  (green – GA variant) | 4 | 6 | 4 | 5 | 19 |
| Samples excluded | 0 | 0 | 1 | 0 | 1 |
| Samples veryfied by sequencing from cluster 1 | 1 | 1 | 1 | 1 | 4 |
| Samples veryfied by sequencing from cluster 2 | 1 | 1 | 1 | 1 | 4 |
| Call rate | - | - | - | - | 0,9974 |

a)
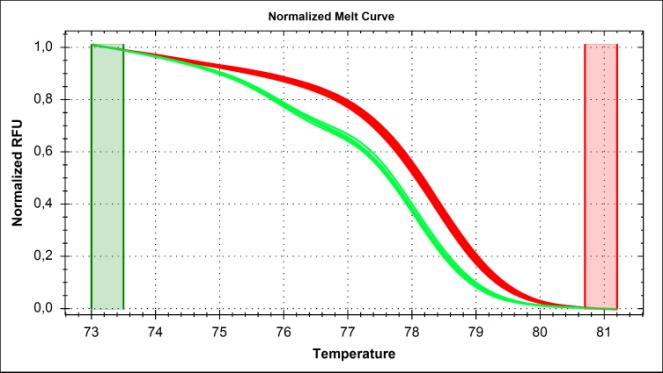

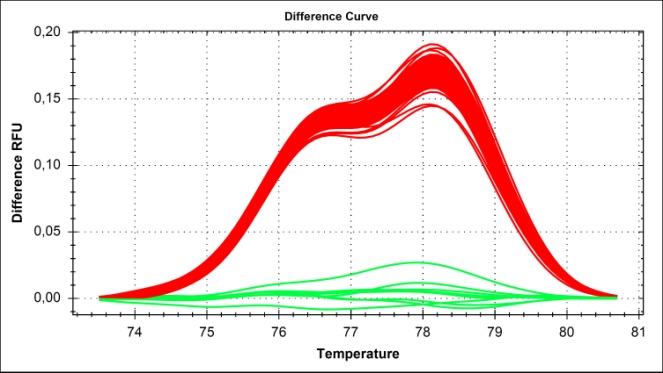
b)
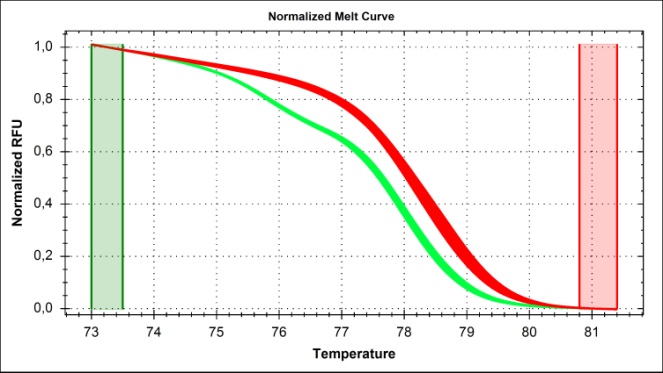

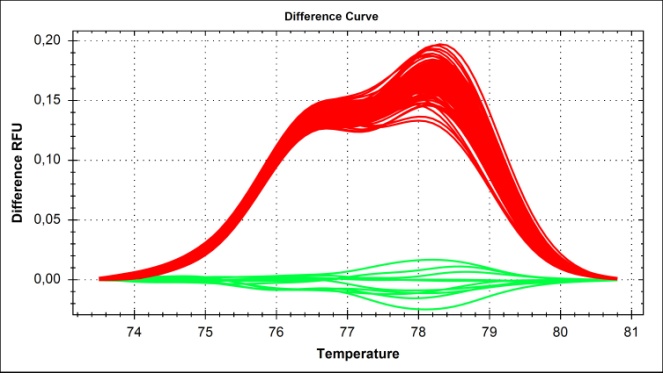
c)
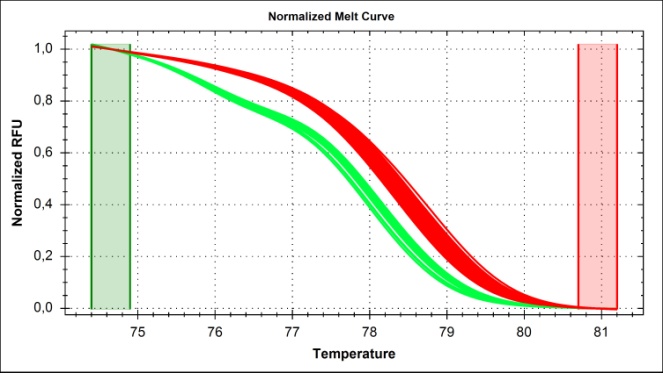

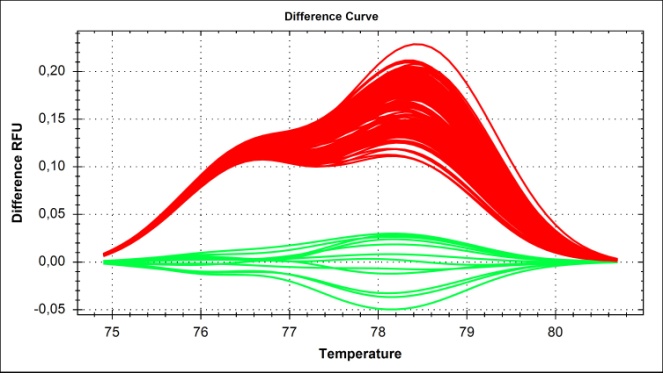


d)
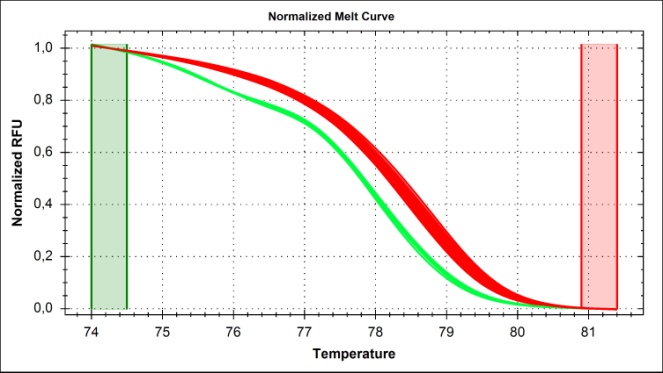

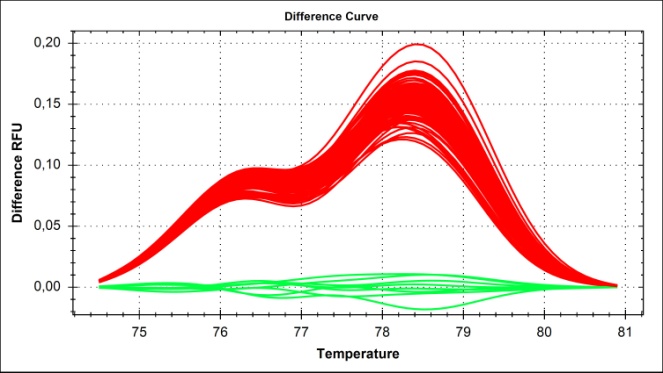


e)
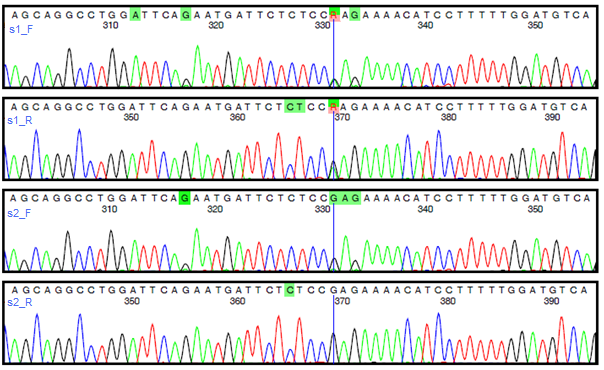


**Figure S57 Summary of *ABCC1* c.2168G>A (p.Arg723Gln, rs4148356) genotyping by HRM.: a,b,c,d – High resolution melting profiles of four different DNA plates (analysed PCR product length: 67 bp including primers, 27 bp without primers, red – cluster 1 (GG), green – cluster 2 (GA); e – Example of the sequencing traces for the samples from the two different clusters: s1_F&R – cluster 2 (GA), s2_F&R – cluster 1 (GG), blue lines indicate c.2168G>A genotype.**

**Table S59 Summary of *ABCC1* c.4002G>A (p.Ser1334=, rs2230671) genotyping by HRM. Call rate is the ratio between positive clustered to the all genotyped samples.**

|  | Plate 1 | Plate 2 | Plate 3 | Plate 4 | Overall |
| --- | --- | --- | --- | --- | --- |
| Samples genotyped | 95 | 95 | 95 | 95 | 380 |
| Melting clusters | 3 | 3 | 3 | 3 | - |
| Samples in cluster 1  (pink – GG variant) | 60 | 48 | 52 | 44 | 204 |
| Samples in cluster 2  (green – GA variant) | 26 | 37 | 39 | 38 | 140 |
| Samples in cluster 3  (blue – AA variant) | 9 | 8 | 4 | 11 | 32 |
| Samples excluded | 0 | 2 | 0 | 2 | 4 |
| Samples veryfied by sequencing from cluster 1 | 1 | 1 | 1 | 1 | 4 |
| Samples veryfied by sequencing from cluster 2 | 1 | 1 | 1 | 1 | 4 |
| Samples veryfied by sequencing from cluster 3 | 1 | 1 | 1 | 1 | 4 |
| Call rate | - | - | - | - | 0,9895 |

a)**
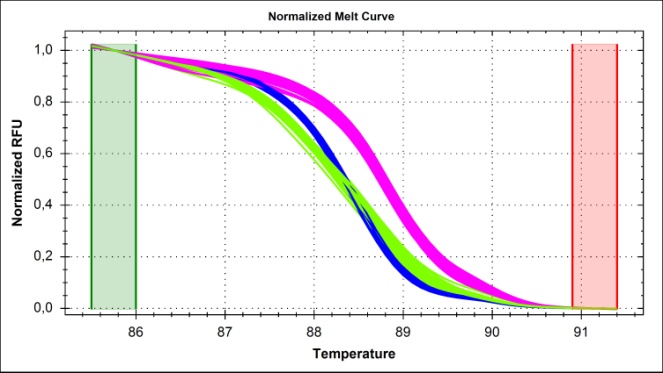

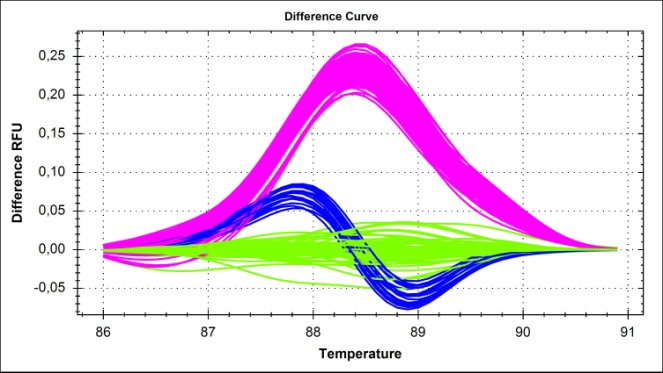
**b)**
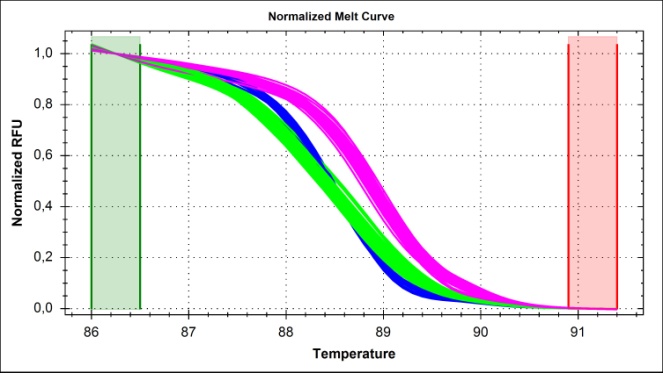

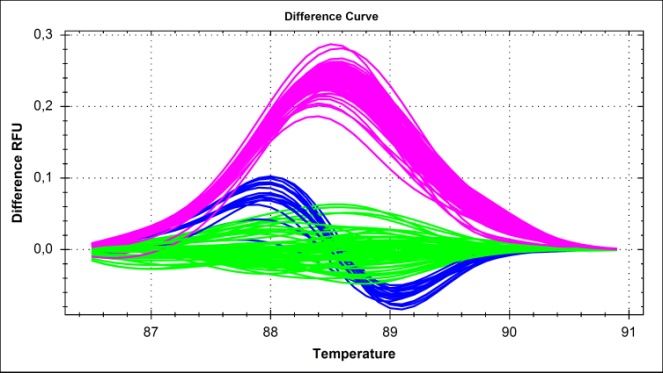
**c)**
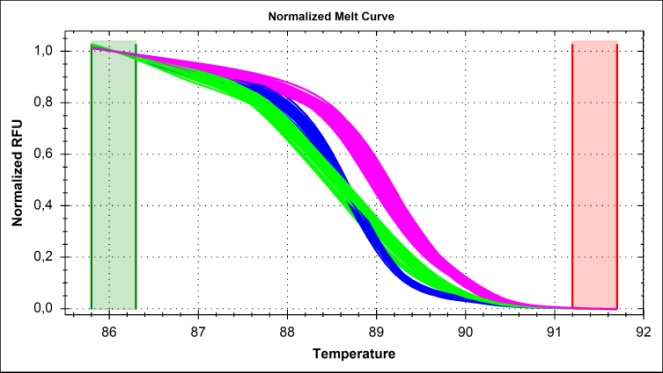

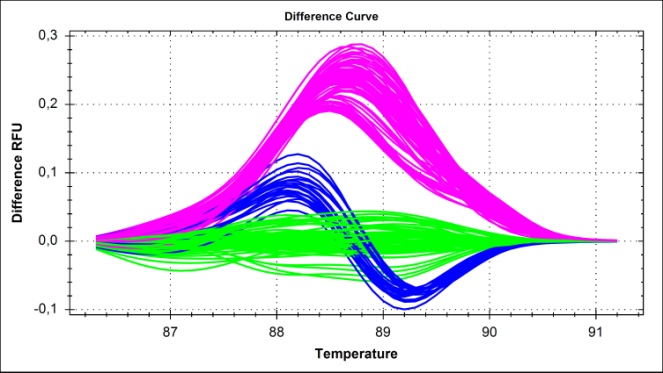
**

d)**
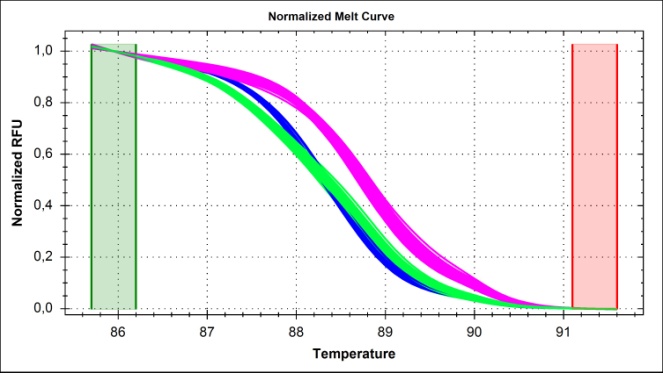

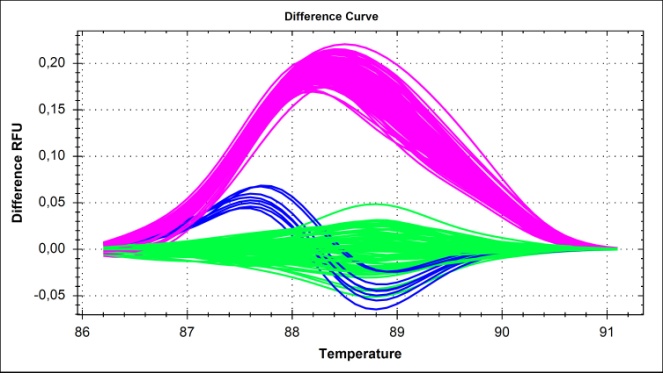
**

e)
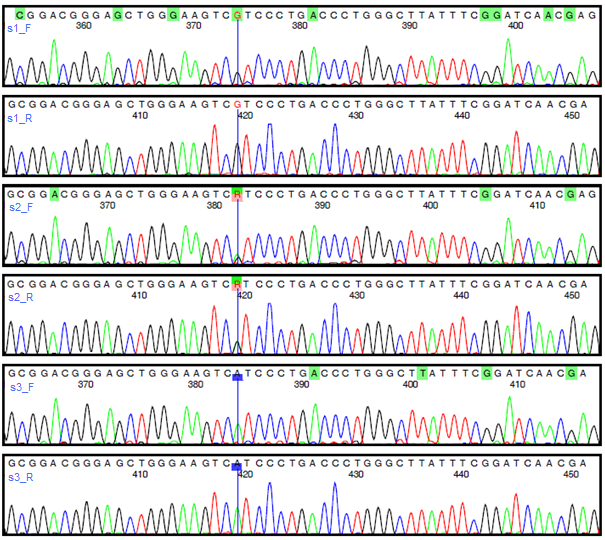


**Figure S58 Summary of *ABCC1* c.4002G>A (p.Ser1334=, rs2230671) genotyping by HRM.: a,b,c,d – High resolution melting profiles of four different DNA plates (analysed PCR product length: 112 bp including primers, 76 bp without primers), pink - cluster 1 (GG), green – cluster 2 (GA), blue – cluster 3 (AA); e – Example of the sequencing traces for the samples from the three different clusters: s1_F&R – cluster 1 (GG), s2_F&R – cluster 2 (GA), s3_F&R – cluster 3 (AA), blue lines indicate c.4002G>A genotype.**

**Table S60 Overall summary of HRM genotyping method**

| dbSNP ID | Variant residue NM_004996.3: | Intron/amino acid residue NP_004987.2: | Clustered samples | Sequenced samples | Excluded samples | All samples | Genotyping rate |
| --- | --- | --- | --- | --- | --- | --- | --- |
| rs2230669 | c.816G>A | p.Pro272= | 380 | 16 | 0 | 380 | 1 |
| rs246221 | c.825T>C | p.Val275 |  |  |  |  |  |
| rs35587 | c.1062T>C | p.Asn354= | 379 | 12 | 1 | 380 | 0,9974 |
| rs35588 | c.1218+8A>G | Intron | 380 | 12 | 0 | 380 | 1 |
| rs60782127 | c.1299G>T | p.Arg433Ser | 379 | 10 | 1 | 380 | 0,9974 |
| rs35605 | c.1684T>C | p.Leu562= | 380 | 23 | 0 | 380 | 1 |
| rs8187858 | c.1704C>T | p.Tyr568= |  |  |  |  |  |
| rs45511401 | c.2012G>T | p.Gly671Val | 377 | 11 | 3 | 380 | 0,9921 |
| rs4148356 | c.2168G>A | p.Arg723Gln | 379 | 8 | 1 | 380 | 0,9974 |
| rs2230671 | c.4002G>A | p.Ser1334= | 376 | 12 | 4 | 380 | 0,9895 |
| **Overall summary** | | | **3030** | **104** | **10** | **3040** | --- |
| **Overall genotyping rate** | | | --- | --- | --- | --- | **0,9967** |
