# Supplementary material for: Genetic variation of the ABC transporter gene ABCC1 (Multidrug resistance protein 1 – MRP1) in the Polish population
Source: BMC Genet. 2015 Sep 23;16:114. doi: 10.1186/s12863-015-0271-3 (PMC4579605; doi:10.1186/s12863-015-0271-3)
Supplement: Additional file 3: — Supplemental materials for structural and functional disruption - in silico analysis using PolyPhen-2 software. (DOCX 13 kb) [file 12863_2015_271_MOESM3_ESM.docx]

**Table S61 Nonsynonymous variants detected in this study and their predicted effect on protein functioning classified by PolyPhen-2 tool as: benign influence, possibly or probably damaging**

| Nonsynonymous variant | HumDiv value | HumDiv result | HumVar value | HumVar result |
| --- | --- | --- | --- | --- |
| c.596C>T (p.Ser199Leu) | 0.982 | probably damaging | 0.520 | possibly damaging |
| c.814C>T (p.Pro272Ser) | 0.000 | benign | 0.001 | benign |
| c.1299G>T (p.Arg433Ser) | 1.000 | probably damaging | 0.999 | probably damaging |
| c.1898G>A (p.Arg633Gln) | 0.001 | benign | 0.002 | benign |
| c.2012G>T (p.Gly671Val) | 1.000 | probably damaging | 0.992 | probably damaging |
| c.2168G>A (p.Arg723Gln) | 0.014 | benign | 0.013 | benign |
| c.2876A>G (p.Lys959Arg) | 0.001 | benign | 0.005 | benign |
| c.3196C>T (p.Arg1066Trp) | 0.786 | possibly damaging | 0.343 | benign |
| c.3886C>T (p.Arg1296Trp) | 0.995 | probably damaging | 0.816 | possibly damaging |
| c.3901C>T (p.Arg1301Cys) | 1.000 | probably damaging | 0.999 | probably damaging |
| c.4093G>A (p.Asp1365Asn) | 0.003 | benign | 0.013 | benign |
|  |  |  |  |  |
