# Supplementary material for: Genetic variation of the ABC transporter gene ABCC1 (Multidrug resistance protein 1 – MRP1) in the Polish population
Source: BMC Genet. 2015 Sep 23;16:114. doi: 10.1186/s12863-015-0271-3 (PMC4579605; doi:10.1186/s12863-015-0271-3)
Supplement: Additional file 4: — Supplemental materials for linkage disequilibrium analysis (include pairwise plots with r 2 and |D'| parameters). (DOCX 327 kb) [file 12863_2015_271_MOESM4_ESM.docx]

**
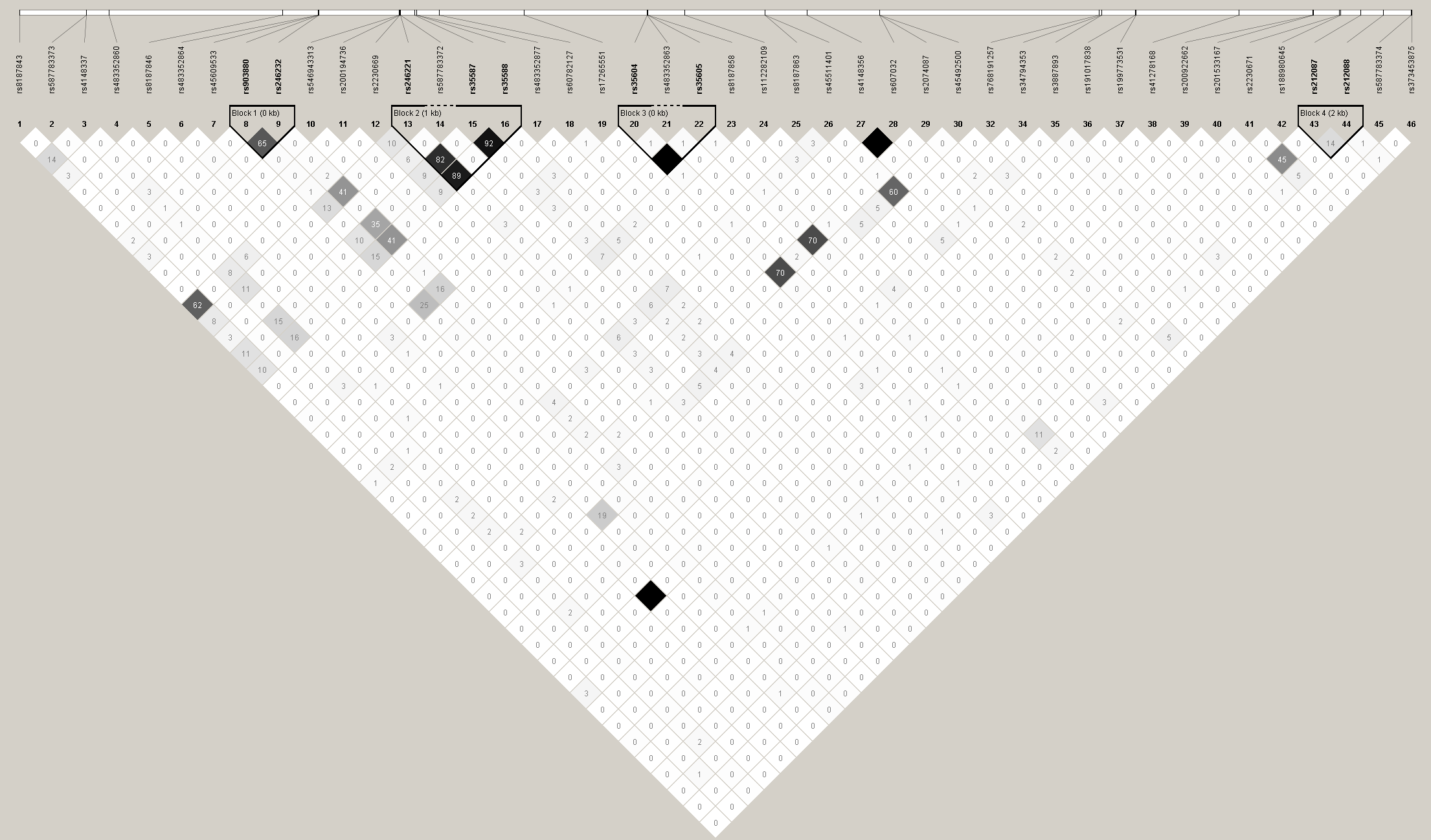
**

**Figure S59 Linkage disequilibrium analysis of ABCC1 SNPs detected in this study using the r^2^ parameter.** Pairwise linkage shown as r^2^ values (x100) by graded grey colors; darker color means closer linkage and lack of value means r^2^=1. Triangles with bold margins show haplotype blocks.


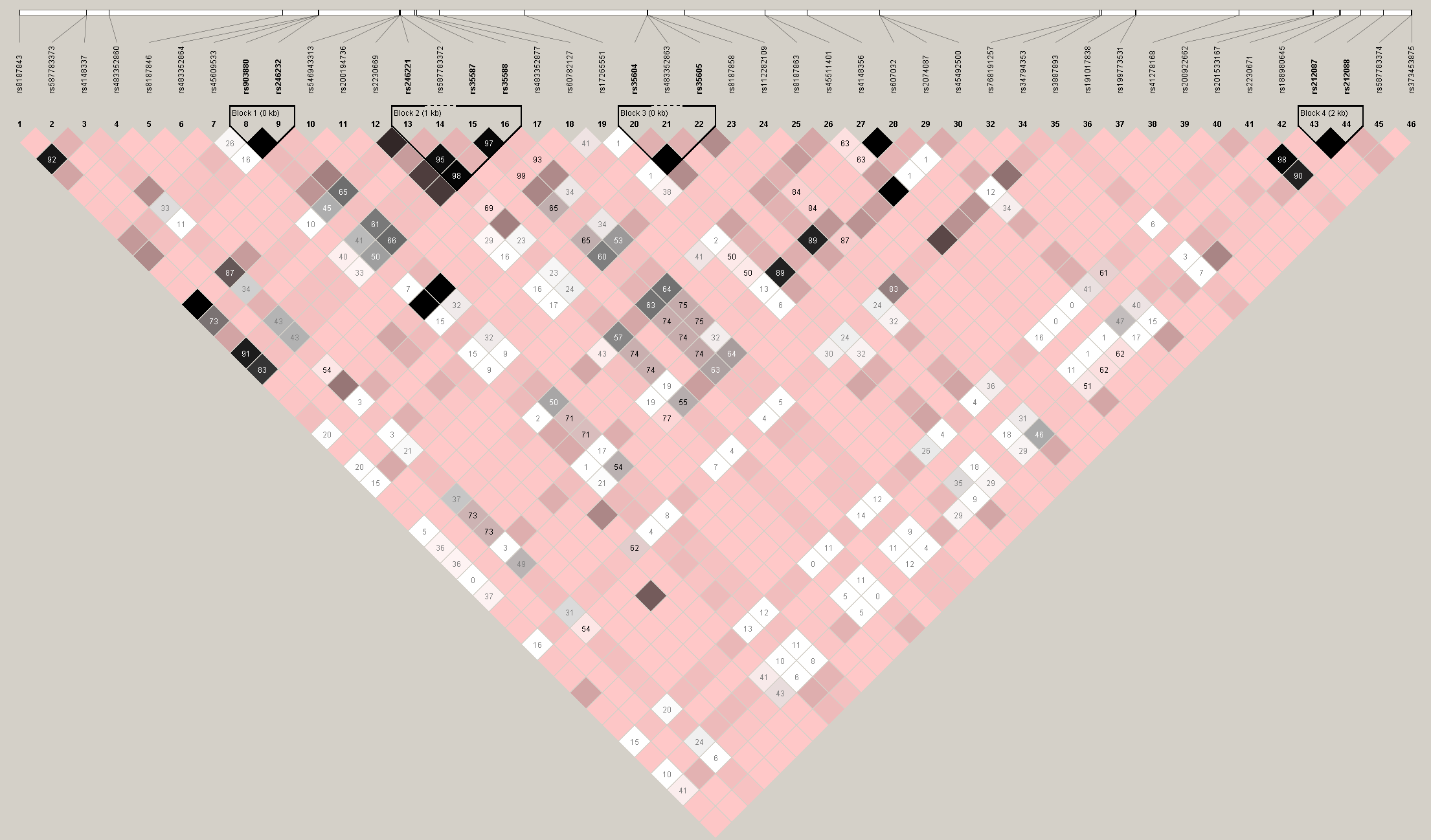


**Figure S60 Linkage disequilibrium analysis of ABCC1 SNPs detected in this study using |D’| parameter.** Pairwise linkage shown as |D’| values (x100) by graded pink and black colors, darker color means closer linkage and lack of value means |D’|=1x100. Triangles with bold margins show haplotype blocks.
